# Ambient particulate matter attenuates Sirtuin1 and augments SREBP1-PIR axis to induce human pulmonary fibroblast inflammation: molecular mechanism of microenvironment associated with COPD

**DOI:** 10.18632/aging.102077

**Published:** 2019-07-12

**Authors:** Chia-Ping Tien, Chia-Hung Chen, Wen-Yuan Lin, Chiu-Shong Liu, Ko-Jiunn Liu, Michael Hsiao, Yu-Chan Chang, Shih-Chieh Hung

**Affiliations:** 1 Ph.D. Program for Aging, College of Medicine, China Medical University, Taichung, Taiwan; 2 Division of Community and Family Medicine, China Medical University Hospital, Taichung, Taiwan; 3 Genomics Research Center, Academia Sinica, Taipei, Taiwan; 4 Pulmonary Medicine, China Medical University Hospital, Taichung, Taiwan; 5 Department of Social Medicine, College of Medicine, China Medical University, Taichung, Taiwan; 6 Department of Family Medicine, College of Medicine, China Medical University, Taichung, Taiwan; 7 National Institute of Cancer Research, National Health Research Institutes, Tainan, Taiwan; 8 Department of Biochemistry, College of Medicine, Kaohsiung Medical University, Kaohsiung, Taiwan; 9 Institute of New Drug Development, China Medical University, Taichung, Taiwan; 10 Integrative Stem Cell Center, China Medical University Hospital, Taichung, Taiwan; 11 Institute of Biomedical Sciences, Academia Sinica, Taipei, Taiwan

**Keywords:** particulate matter, Sirtuin1, SREBP1, Pirin, inflammasomes

## Abstract

Evidences have shown a strong link between particulate matter (PM) and increased risk in human mortality and morbidity, including asthma, chronic obstructive pulmonary disease (COPD), respiratory infection, and lung cancer. However, the underlying toxicologic mechanisms remain largely unknown. Utilizing PM-treated human pulmonary fibroblasts (HPF) models, we analyzed gene expression microarray data and Ingenuity Pathway Analysis (IPA) to identify that the transcription factor *sterol regulatory element-binding protein 1 (SREBP1)* was the main downstream regulator of *Sirtuin1* (*SIRT1*)*.* Quantitative PCR and western blot results showed that SIRT1 inhibited SREBP1 and further downregulated Pirin (PIR) and Nod-like receptor protein 3 (NLRP3) inflammasome after PM exposure. Inhibitors of SIRT1, SREBP1, and PIR could reverse PM-induced inflammation. An *in silico* analysis revealed that PIR correlated with smoke exposure and early COPD. Immunohistochemical analysis of tissue microarrays from PM-fed mouse models was used to determine the association of PIR with PM. These data demonstrate that the SIRT1-SREBP1-PIR/ NLRP3 inflammasome axis may be associated with PM-induced adverse health issues. SIRT1 functions as a protector from PM exposure, whereas PIR acts as a predictor of PM-induced pulmonary disease. The SIRT1-SREBP1-PIR/ NLRP3 inflammasome axis may present several potential therapeutic targets for PM-related adverse health events.

## INTRODUCTION

Epidemiologic studies have suggested a strong link between air pollution and increased risk in human mortality and morbidity, including asthma, chronic obstructive pulmonary disease (COPD), pulmonary infection, and lung cancer. Meanwhile, air pollutants can lead to serious health consequences including stroke, Alzheimer’s disease, heart disease, and diabetes. Children, pregnant women, and the elderly are especially vulnerable [1–3]. World Health Organization (WHO) recognizes that fine particles in polluted air is an invisible killer responsible for around 7 million global deaths per year, including 43% from chronic obstructive pulmonary disease, 29% from lung cancer, 25% from heart diseases, 24% from stroke, and 24% from acute lower respiratory infection [1, 4].

The sources of air pollution include industry and energy supply, transport, waste management, dust, agricultural practices, and household energy [3, 5]. Ambient particulate matter (PM) is defined as airborne particles, a mixture of mixtures solid and liquid droplets, composed of alkanes, carbons, aromatic acids, sulfate, nitrate, and ions [6]. Although the underlying toxicologic mechanisms by which PM induces adverse health events remain to be elucidated, it is believed that it is due to its unique characteristics, small size, large surface area, and ability to absorb a large amount of harmful molecules [3]. PM is also considered as a potent oxidants and capable of directly generating reactive oxygen species (ROS), followed by cellular oxidative stress. Oxidative stress can activate redox-sensitive signaling pathways that lead to different biological cascades, including inflammation, DNA damage, and cell death [7]. Additionally, inflammasomes, consist of caspase-1, apoptosis-associated speck-like protein containing a CARD (ASC) and Nod-like receptor protein 3 (NLRP3), imply a wide variety of chronic or acute inflammations, including lung disease [8, 9]. NLRP3 inflammasome activates caspase-1, cleaving the precursor IL-1β and IL-18 into bioactive cytokines, which trigger further inflammatory cascade [8]. Several studies indicate that inflammasomes play a key role in PM-induced lung disease, central nervous system toxicity, cardiovascular disease, reproductive disorders, and skin injury [8, 10–13]. PM, when inhaled, easily bypasses the complete respiratory airway, penetrates alveoli and crosses the air-blood barrier, leading to increased risk in human mortality, morbidity, and hospitalizations, especially in COPD [1–3, 14]. In the current study, we sought to define the role of the inflammasome complex and its subsets in the pathogenesis of PM-related health problems.

It was estimated that COPD, a progressive life-threatening lung disease, caused more than 3 million global deaths in 2012 [15]. Major risk factors of COPD include tobacco smoke, air pollution, occupational dust, chemicals, and respiratory infections [15, 16]. COPD should be suspected in any patient with symptoms of chronic cough, sputum, shortness of breath, and/or history of exposure to noxious stimuli. Global Initiative for Chronic Obstructive Lung Disease (GOLD) guidelines for COPD state diagnosis are established by spirometry with a post-bronchodilator forced expiratory volume in one second (FEV_1_) / forced vital capacity (FVC) < 0.70, consistent with the presence of irreversible and progressive airflow limitation [15]. COPD can develop adverse sequelae, including pneumonia, lung cancer, polycythemia, hypertension, cor pulmonale, osteoporosis, skeletal muscle dysfunction, weight issues, sleep apnea, diabetes, depression, and anxiety [15].

In the current study, we hypothesized that the inflammation and tissue remodeling mediated by PM may arise from an alternation of gene expression or signaling pathways via inflammasomes, developing respiratory disease. We established transcriptomics data by microarray chips in human pulmonary fibroblasts (HPF), a central player in the fibrosis and airway remodeling of COPD [9], after exposure to PM. Our results confirmed that sterol regulatory element-binding protein 1 (SREBP1) and its downstream target Pirin (PIR) were activated by PM, which could be counteracted by Sirtuin1 (SIRT1). Moreover, we provided evidence that SREBP1 is directly inhibited by SIRT1, and increases expression of inflammasome-related markers in PM-treated HPF cell models [17]. In addition, *in silico* analysis predicted that PIR played a key role in early COPD events. Taking all results into account, therapeutic targeting of SIRT1-SREBP1-PIR/ NLRP3-inflammasome axis may be a novel approach against PM-induced fibrosis and inflammation in the human respiratory system.

## RESULTS

### *SREBP1* acts as the activated transcription factor upon PM treatment in HPF

**Figure 1:**
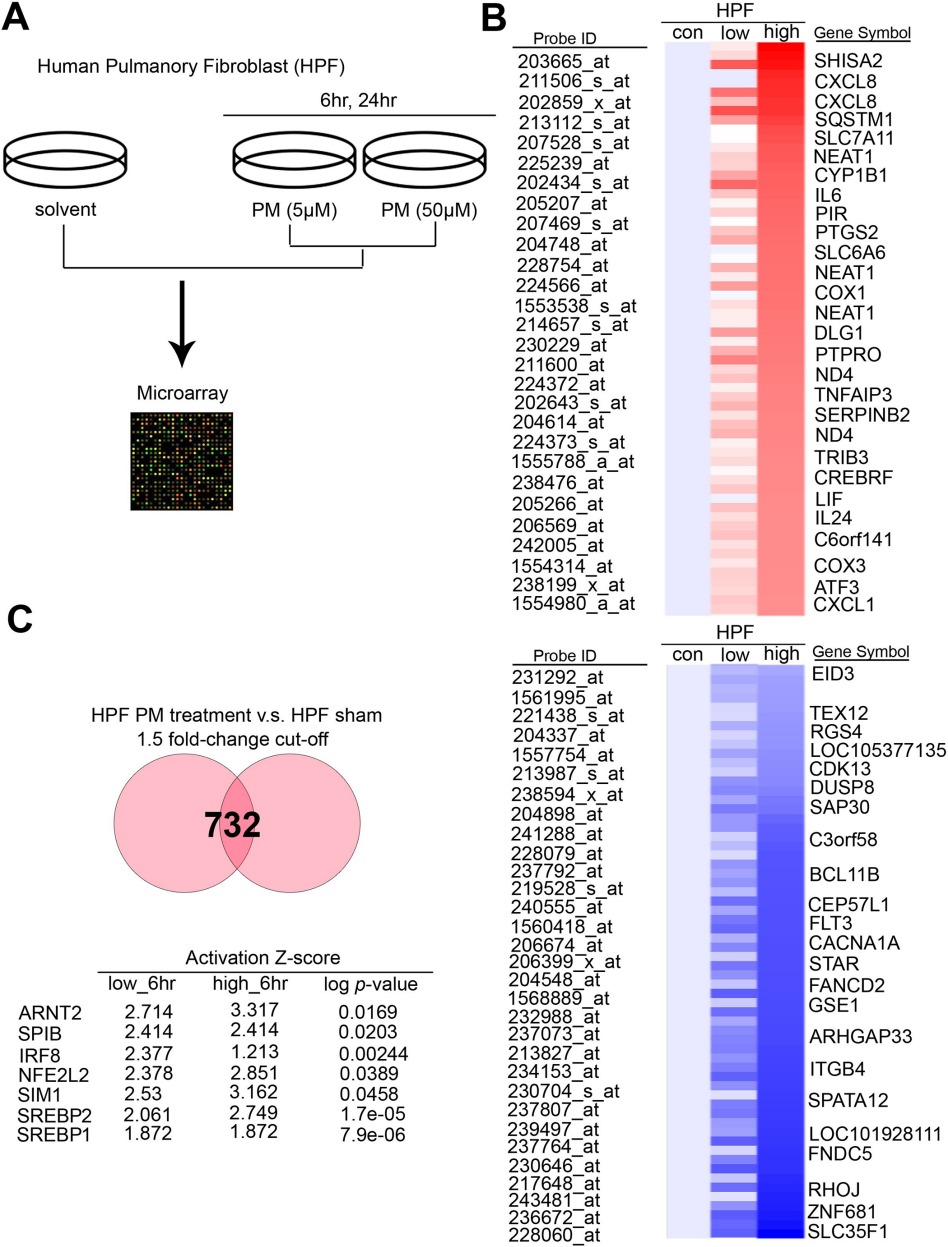
**PM induced several potential transcription factors in a dose-dependent manner in normal lung cells.** (**A**) The flowchart represents the procedure of microarray chips established in human pulmonary fibroblasts. (**B**) Detailed heatmaps highlight the significantly up- and down-regulated genes after PM treatment, that were normalized gene expression from the microarray database analysis. (**C**) The ranking of candidate transcription factors by IPA database of microarray from PM treatment compared with the sham group in human pulmonary fibroblasts. The cut-off was a 1.5-fold change.

**Figure 2:**
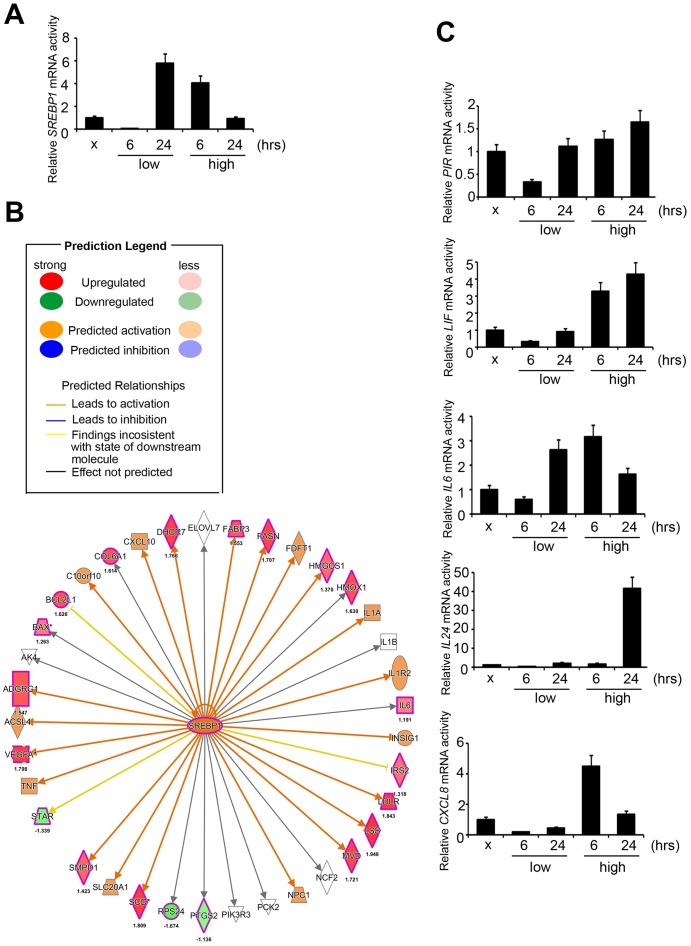
**Signatures regulated by PM in HPF models.** (**A**) The mRNA level of *SREBP1* after PM treatment. (**B**) The prediction of *SREBP1*-network derived from the common signature, by comparison of IPA database with microarray data from HPF cells with a 1.5-fold-change cut-off. The intensity of the node color indicates the degree of activating (orange) or inhibiting (blue) regulation following PM interaction. (**C**) Several mRNA levels of *SREBP1* downstream factors, including *PIR, LIF, IL-6, IL-24,* and *CXCL8,* after PM treatment in HPF.

To determine the exact canonical pathways or networking induced by PM in the human respiratory system, we evaluated transcriptomics datasets by microarray chips in HPF (Human Pulmonary Fibroblast) under several PM groups (sham, low dose for 6 hours, low dose for 24 hours, high dose for 6 hours, high dose for 24 hours, low dose = 5 μM, high dose = 50 μM) (Figure 1A). Through Genespring software normalization, 732 genes were found to have > 1.5-fold differential expression in low for 6 hours, low for 24 hours, high for 6 hours, or high for 24 hours relative to sham in HPF (Supplementary Table 1). The top ranking of candidate genes is shown in Figure 1B. The normalized microarray data was analyzed using IPA to identify significant molecular that are activated in response to PM exposure in HPF. The results showed that the transcription factor *SREBP1* was the No. 1 putative candidates to be activated in response to PM exposure, with the transcriptional activity of its downstream genes of *P* = 7.9E-06 in HPF. The top 7 transcription factors in HPF are shown in Figure 1C. There are 2 *SREBP* genes in mammals, *SREBP1* and *SREBP2*, encoding 3 sterol regulatory element-binding proteins (SREBPs), SREBP-1a, -1c and -2 [18]. *SREBP-1a* acts as a potent activator of all SREBP-responsive genes, stimulating cholesterol, fatty acids, and triglycerides synthesis. SREBP-1c is the main regulator of fatty acid, whereas SREBP-2 is the main regulator of cholesterol [18]. We sought to study if *SREBP1* acts as transcription factors in response to PM, and our quantitative RT-PCR analysis result showed that *SREBP1* was consistent with microarray in HPF (Figure 2A). *SREBP1* was significantly increased in a dose-dependent manner after PM for 6 hours and in a time-dependent manner after PM at low dose. We uploaded our microarray datasets into the SREBP1-related signature from IPA tool, and our analysis showed that *C-X-C motif chemokine ligand 8 (CXCL8), interleukin 6 (IL-6), Pirin (PIR), Interleukin 6 Family Cytokine (LIF), and Interleukin 24 (IL-24)* were the most significantly upregulated transcriptional targets of *SREBP1* (Figure 2B).

### SREBP1 activated PIR in response to PM exposure

We further examined the levels of *SREBP1* and its downstream targets, *CXCL8, IL-6, PIR, LIF,* and *IL-24* in the PM treatment groups (sham, low for 6 hours, low for 24 hours, high for 6 hours, high for 24 hours) of HPF. Our quantitative RT-PCR analysis results first showed that *IL-6* and *PIR* were consistent with microarray in HPF, whereas *CXCL8, LIF,* and *IL-24* were not consistent (Figure 2C). Second, response of *PIR, IL-24,* and *LIF* to PM was in a time- and dose-dependent manner. Response of *CXCL8* to PM was found to be in a dose-dependent manner and only in a time-dependent manner at low dose. Response of *IL-6* to PM was only in a dose-dependent manner for 6 hours and only in a time-dependent manner at low dose. Therefore, we assessed the expression of PIR at the protein level in response to PM treatment.

**Figure 3:**
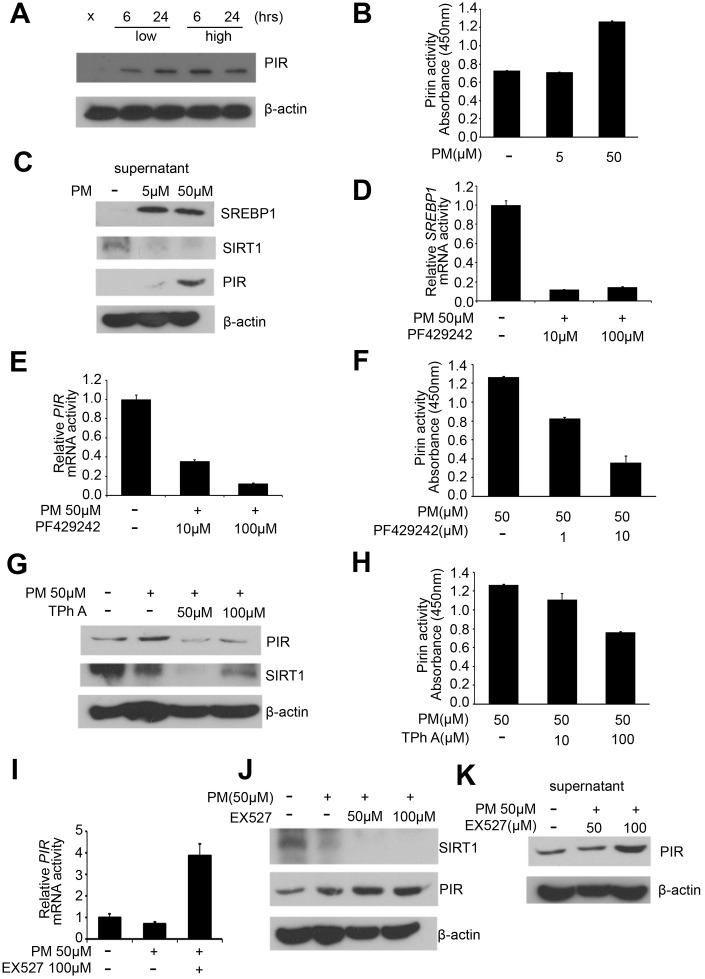
**PM exposure is positively correlated with activation of the SREBP1-PIR signaling pathway through SIRT1 downregulation.** (**A**) Western blot analysis of PIR after PM treatment in HPF. (**B**) PIR activity from cell culture supernatant in PM-induced models. (**C**) Levels of SREBP1, SIRT1, and PIR were measured from cell culture supernatant of PM-induced models. (**D**) mRNA levels of *SREBP1* in PF429242-PM co-treated HPF models. (**E**) mRNA levels of *PIR* in PF429242-PM co-treated HPF models. (**F**) PIR activity from cell culture supernatant in PF429242-PM co-treated HPF models. (**G**) Western blot analysis of PIR and SIRT1 in TPh A-PM co-treated HPF models. (**H**) PIR activity from cell culture supernatant in TPh A-PM co-treated HPF models. (**I**) mRNA level of *PIR* in Ex527-PM co-treated HPF models. (**J**) Western blot analysis of SIRT1 and PIR in Ex527-PM co-treated HPF models. (**K**) Level of PIR was measured from cell culture supernatant in Ex527-PM co-treated HPF models.

Our western blot results in the PM treatment groups (sham, low for 6 hours, low for 24 hours, high for 6 hours, high for 24 hours) of HPF showed that PIR were significantly overexpressed after exposure to PM. PIR was significantly increased in a dose-dependent manner for 6 hours and only in a time-dependent manner at low dose (Figure 3A). At present, SREBP1 is a well-established negative target of SIRT1 [17, 19]. We further hypothesize that PIR is negatively associated with SIRT1.

### Increased PIR level and activity in cell culture supernatant are significantly correlated with PM exposure

PIR has been identified to possess enzymatic activity [20]. Moreover, delocalization of PIR was identified in melanomas [20]. To address the role of delocalization of PIR, PIR levels and PIR activity in cell culture supernatant of PM-induced models were determined. Our analysis showed that PIR enzymatic activity was significantly overexpressed after high-dose PM exposure (Figure 3B). Furthermore, we examined levels of SREBP1, SIRT1, and PIR in cell culture supernatant. Our result showed that SREBP1 and PIR were significantly increased in a dose-dependent manner, while SIRT1 was significantly suppressed in a dose-dependent manner after PM exposure, which was consistent with our hypothesis that PM exposure can activate the SREBP1-PIR axis via SIRT1 suppression (Figure 3C).

### PM exposure can activate the SREBP1-PIR axis, which could be suppressed by SIRT1

In order to validate that PM exposure can activate the SREBP1-PIR axis, which could be suppressed by SIRT1, we administrate the SIRT1 inhibitors Ex527 [21], SREBP1 inhibitors PF429242 [22], and PIR inhibitors TPhA [23] in PM-treated HPF models. First, we administered PF429242 (10 and 100 μM) to PM-treated HPF models, and mRNA level of *SREBP1* was significantly decreased as expected (Figure 3D). The mRNA level of *PIR* was significantly decreased in a dose-dependent manner in response to PF429242 in PM-treated HPF models (Figure 3E). We further examined cell culture supernatant in PF429242-PM co-treated models, and our analysis showed that PIR activity was significantly inhibited by PF429242 in a dose-dependent manner (Figure 3F). These results support the hypothesis that the SREBP1-PIR axis was activated by PM. Second, we administered TPh A (50 and 100 μM) to PM-treated HPF models, and protein levels of PIR were significantly decreased as expected (Figure 3G). Strikingly, the expression of SIRT1 was correlated with the expression of PIR on the protein levels in TPh A-PM co-treated HPF models (Figure 3G). We further examined the cell culture supernatant in TPh A-PM co-treated models, and our analysis showed that PIR activity was significantly inhibited in a dose-dependent manner as expected (Figure 3H). Third, we administered Ex527 (100 μM) to PM-treated HPF models, and our quantitative RT-PCR analysis showed that *PIR* was significantly overexpressed (Figure 3I). Afterwards, we further performed western blot analysis. We found Ex527 (50 and 100 μM) suppressed the expression of SIRT1 as expected, while Ex527 increased the expression of PIR in a dose-dependent manner (Figure 3J). Our analysis further showed that PIR in cell culture supernatant was significantly increased in a time- and dose-dependent manner in Ex527-PM co-treated models, which was consistent with expression on PIR protein level (Figure 3J, 3K).

### NLRP3 inflammasome may be activated by PM exposure via SREBP1-PIR axis

**Figure 4:**
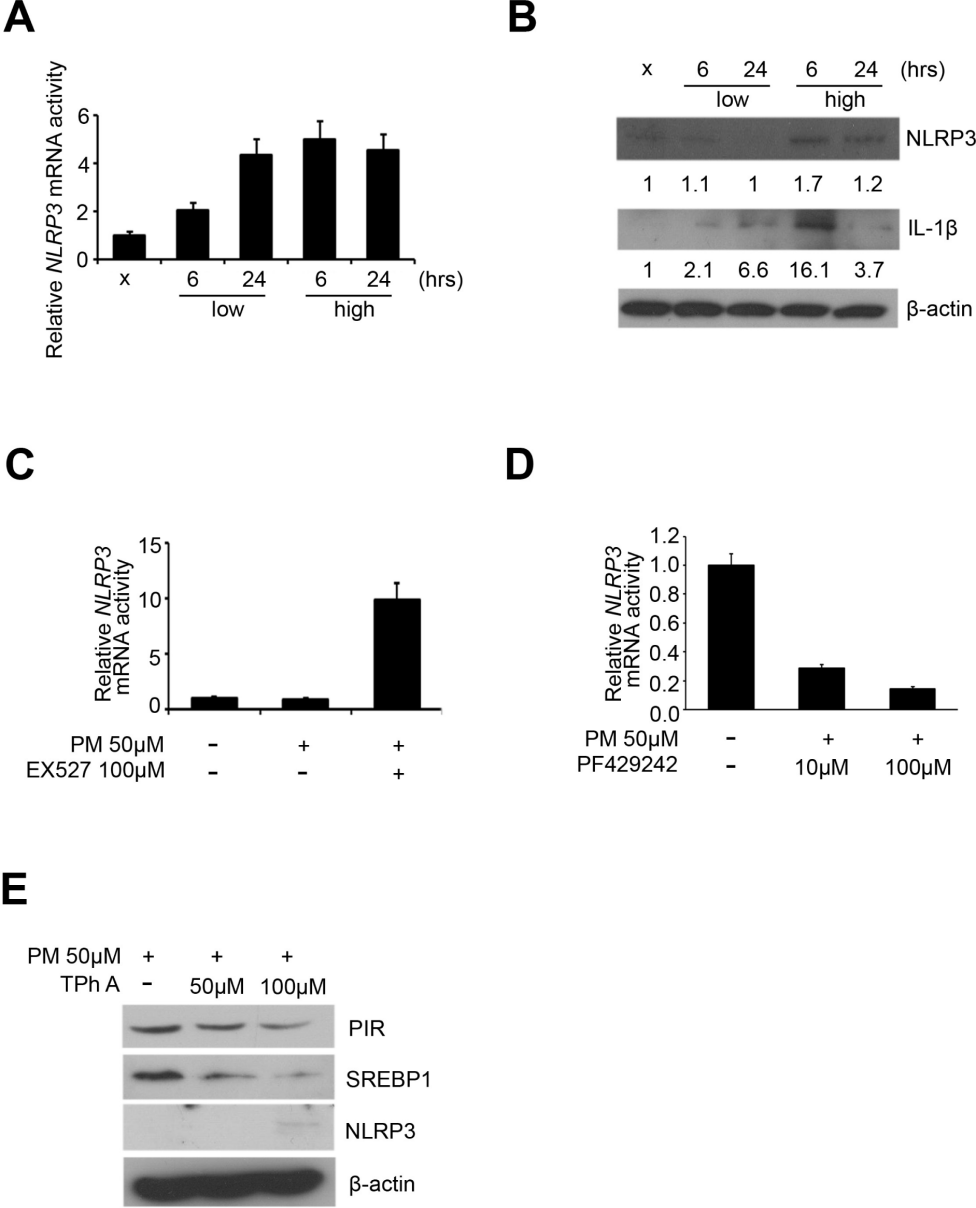
**NLRP3 inflammasome was upregulated by PM exposure.** (**A**) mRNA level of *NLRP3* after PM in HPF. (**B**) Western blot analysis of NLRP3 and IL-1β after PM in HPF. (**C**) mRNA level of *NLRP3* in Ex527-PM co-treated HPF models. (**D**) mRNA levels of *NLRP3* in PF429242-PM co-treated HPF models. (**E**) Western blot analysis of PIR, SREBP1, and NLRP3 in TPh A-PM co-treated HPF models.

Previous studies have identified that the protein complex called inflammasomes, play a central role in the acute lung injury and chronic pulmonary diseases [8]. NLRP3 and IL-1β were considered as characteristic markers of inflammasomes [8]. We first carried out quantitative RT-PCR analysis to detect the expression of *NLRP3* in the 5 PM treatment groups (sham, low for 6 hours, low for 24 hours, high for 6 hours, high for 24 hours) of HPF, and our results showed that *NLRP3* was overexpressed in dose-dependent manner in response to PM and only in a time-dependent manner at low dose (Figure 4A). We further carried out western blot to detect the expression of IL-1β and NLRP3 in the 5 PM treatment groups of HPF. After normalized against β-actin and quantified via ImageJ, NLRP3 was overexpressed in a dose-dependent manner for both 6 and 24 hours, while IL-1β was overexpressed in a dose-dependent manner for 6 hours and in a time-dependent manner at low dose (Figure 4B). Previous studies indicate that SREBP1, directly deacetylated and inhibited by SIRT1, increases expression of inflammasomes [17]. Thus, we hypothesized that exposure to PM suggests induction of the SREBP1-NLRP3 inflammasome pathway, via SIRT1 inhibition. We further validated the mRNA expression of *NLRP3* in Ex527-PM and PF429242-PM co-treated HPF models. By quantitative RT-PCR analysis, Ex527 significantly upregulated *NLPR3*, while PF429242 significantly inhibited *NLRP3* in a dose-dependent manner (Figure 4C, 4D). In Tph A-PM co-treated HPF models, the expression of PIR and SREBP1 were both downregulated in a dose-dependent manner, while NLRP3 was upregulated in a dose-dependent manner (Figure 4E).

### PIR acts as a biomarker for detecting early COPD

**Figure 5:**
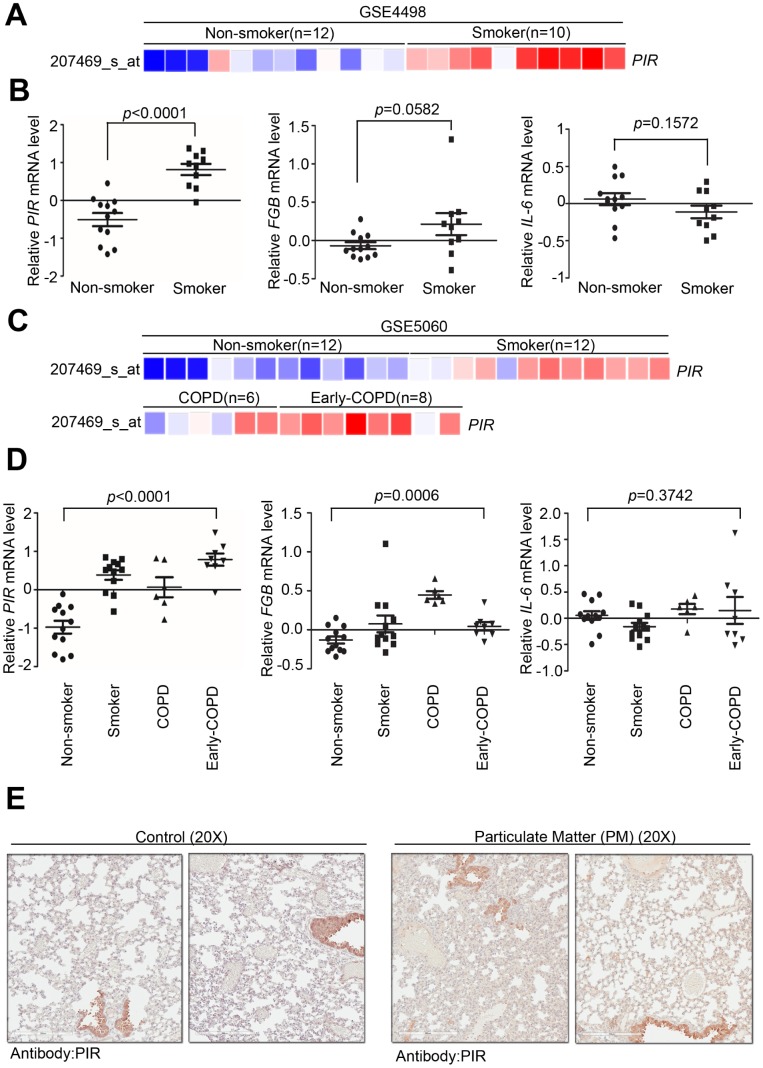
**Overexpression of PIR correlates with smoke and early COPD.** (**A**) Detailed heatmap highlights the correlation between the mRNA expression level of *PIR* and smoke in the GSE4498 cohort (n = 22) using Oncomine online analysis tool. (**B**) Differential mRNA levels of *PIR*, *FBG*, and *IL-6* between non-smoker and smoker in the GSE4498 cohort. (**C**) Detailed heatmaps highlight the correlation between the mRNA expression levels of *PIR* and non-smoker, smoker, COPD, and early COPD in the GSE5060 cohort (n = 38) using Oncomine online analysis tool. (**D**) Differential mRNA levels of *PIR*, *FBG*, and *IL-6* in non-smoker, smoker, COPD, and early COPD in the GSE5060 cohort (n = 38) in the analysis by the Oncomine online analysis tool. (**E**) The immunohistochemical staining results showed PM-fed mice group had higher concentration of the PIR protein in the lung tissue.

To determine the clinical association between PIR and PM, we verified several previously developed microarray databases to identify and compare the correlation between *PIR* and nonsmokers, smokers, early COPD, and COPD using the Gene Expression Omnibus (GEO) databases. By analyzing a microarray cohort collecting small airway epithelium from phenotypically normal smokers and non-smokers, relative to nonsmokers, *PIR* was found to be the most activated gene to have a significant correlation with smoking (Figure 5A-5B, *P* < 0.0001) (GSE4498, n = 22). An *in vitro* study, assessing human airway epithelial cells, identified that air pollution stimulates a time- and dose- dependent increase in mRNA level of *IL-6* via nuclear factor kappa B (NF-κB) mediated ROS [24]. Moreover, a multicenter longitudinal study that assessed 955 myocardial infarction survivors from six European cities identified a strong correlation between *fibrinogen beta chain (FGB)* and *IL-6* and worse outcome after exposure to air pollutions [25]. Herein, we studied whether smoke exposure could induce genetic expression of *FGB* and *IL-6.* Our analysis showed that *FGB* and *IL-6* had no significant association with smoking (Figure 5B, *P* = 0.0582 and *P* = 0.1572, respectively) (GSE4498, n = 22). In another microarray database comprised of large airway epithelium from phenotypically normal smokers, non-smokers, early COPD, and COPD, *PIR* was found to have a strong correlation with smoking (Figure 5C, GSE5060, n = 38). We further studied whether severity of COPD was correlated with genetic expression of *PIR*, *FGB,* and *IL-6.* Notably, relative to COPD, *PIR* was found to be the most activated gene to have a significant correlation with early COPD (Figure 5C-5D, *P* < 0.0001), followed by *FGB* (Figure 5D, *P* = 0.0006), while *IL-6* had no significant association (Figure 5D, *P* = 0.3742) (GSE5060, n = 38). In order to validate if PIR is far superior in detecting early COPD, we next identified PIR protein expression using our own clinical cohort. By intra-tracheal instillation of PM into lung using mice models, we collected tissue sections from lung to undergo pathologic exam and immunohistochemical examination. The immunohistochemical staining results showed that the PM-treated mice group had higher concentration of the PIR protein in the lung tissue than in the control mice group (Figure 5E).

**Figure 6:**
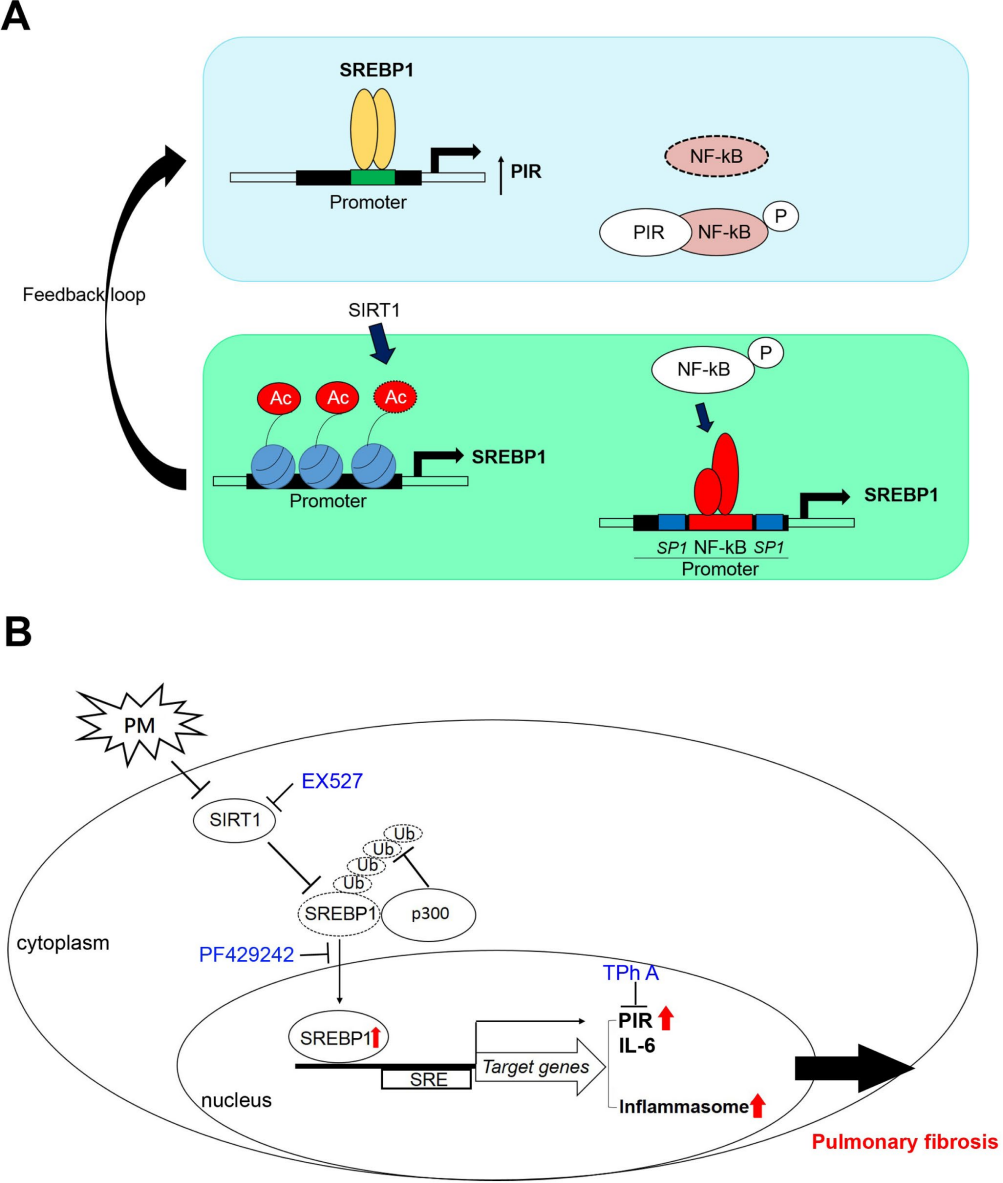
**A model illustrates that PM activates the SREBP1-PIR/inflammasomes signaling axis through SIRT1 -mediated modulation.** (**A**) A schematic model to describe the positive feedback loop and detailed mechanisms of the SIRT1-SREBP1-PIR axis and crosstalk with NF-κB signaling. (**B**) SIRT1 modulates the SREBP1-PIR/NLRP3 inflammasome axis in response to PM. SIRT1 functions as a protector from PM exposure. The SIRT1-SREBP1-PIR/NLRP3 inflammasome axis may present an attractive therapeutic target for PM-related adverse health events.

These data suggested that high PIR protein expression was significantly correlated with PM exposure, which means PIR acts as a predictor of PM-induced cardiopulmonary disease. Together, we identified that PM exposure can activate the SREBP1-PIR/NLRP3 inflammasome signaling pathway, through SIRT1 downregulation (Figure 6A, 6B). The SIRT1-SREBP1-PIR/ NLRP3 inflammasome axis may present several potential therapeutic targets for PM-related adverse health consequence.

## DISCUSSION

PM exposure is believed to be strongly linked to systemic diseases, majorly cardiopulmonary diseases [1, 4]. Alternate NF-κB redox-regulated signaling pathways have been identified to be a mediator of PM-mediated inflammation and carcinogenesis [26]. PIR, encoded by the *PIR* gene, acts as an iron-dependent redox regulator of NF-κB. PIR is a nuclear protein detectable in all human tissues [27]. Though the function of PIR has not been well studied, it is known to stabilize the formation of quaternary complexes between oncoprotein B-cell lymphoma 3-encoded (Bcl-3), anti-apoptotic protein NF-κB, and its target DNA [28]. Air pollution was identified to activate *IL-6* expression in human airway epithelial cells via NF-κB [24], though in our analysis *IL-6* had no significant association with smoking, whereas PIR was significantly correlated with smoking. PM induces adverse health events via multi-factorial mechanisms (Supplementary Figure 1); therefore, the existence of crosstalk between IL-6 and PIR needs to be addressed. Several intensive researches in the field of cancer show that PIR may act as a relevant role in melanoma and acute myeloid leukemia [23, 29]. A recent *in vitro* study demonstrated that increased expression of PIR was induced by smoke [30], whereas another *in vivo* study on 13 phenotypically normal smokers and 9 non-smokers showed that overexpression of PIR played a role in lung epithelial cell apoptosis in response to cigarette smoke [28]. Surprisingly, we found that PIR could be a biomarker for detecting early COPD. The lack of overexpression of PIR in COPD was surprising because PIR-related apoptosis was thought to result COPD [28]. In contrast to our result however, upregulation of PIR from the human small airway epithelium (n = 33) was consistent with the pathogenesis of COPD [31]. Indeed it was previously documented that tobacco-induced emphysema was independent of NF-κB [32]. Reduction of *PIR* was detected in neonatal mouse respiratory distress disease by cDNA microarray analysis [33]. The plausible explanation is that COPD is a multifactorial and complex disease, involved in variety of factors and cells [15], the inhibition or inactivation of PIR in each stage of COPD need further elucidated.

Previous *in vitro* and *in vivo* researches suggest that SIRT1 negatively regulates NF-κB-proinflammatory axis in lung disease, including COPD and allergic asthma [34]. Moreover, a recent study demonstrated that SIRT1 functions as a protector from inflammation and coagulation via modulating NF-κB activation following PM exposure [35]. The mechanism of SIRT1 regulation in response to PM, nevertheless, remains to be elucidated. Our data showed that PM inhibited SIRT1 activity, which confirmed the protective role of SIRT1 in response to PM. A feedback loop between SIRT1 and NF-κB had been documented [36]. SIRT1 mRNA and protein levels as well as SIRT1 promoter activity upregulated in NF-κB overexpression cell models, while SIRT1 protein expression and SIRT1 promoter activity decreased in NF-κB knockdown cell models. Our results revealed that the expression of SIRT1 was correlated with the expression of PIR at the protein levels in TPh A-PM co-treated HPF models (Figure 3G), which supported the hypothesis that SIRT1 may interact with NF-κB inflammation via PIR. Our results further revealed that PM exposure can activate SREBP1-PIR signaling pathway through SIRT1 downregulation. Therefore, SIRT1 may interfere with NF-κB inflammation via the SREBP1-PIR signaling pathway.

In addition to disrupting inflammation, recent studies demonstrated that SIRT1 inhibits NF-κB to suppress various metabolic diseases, including glucose homeostasis and cardiovascular disease [35, 37]. However, the crosstalk controlling metabolism between SIRT1 and NF-κB remains unclear. Several *in vitro* studies revealed that SIRT1-SREBP1 protect mice from hepatic lipogenesis [19, 38]. A study showed that fenofibrate, clinically used to reduce lipid levels, prevented rabbits with atrial fibrillation from atrial metabolic remodeling through the SIRT1-SREBP1 pathway [39]. Another study showed that fenofibrate regulated hyperglycemia-induced metabolic memory via SIRT1-dependent suppression of NF-κB in human retinal endothelial cells [40]. In addition, fenofibrate inhibited the inflammatory response via SIRT1-dependent suppression of NF-κB in adipocytes [41]. On the basis of previous work and this study, SIRT1 may acts as a protector from PM-related consequences, including cardiopulmonary and metabolic disease, by interfering in NF-κB inflammation via SREBP1-PIR signaling pathway. Interestingly, both *in vitro* and *in vivo* experiments documented that NF-κB-mediated activation of SREBP1 pathway increased lipid biosynthesis through binding to promoter region [42]. Previously studies have also claimed that PIR acts as a cofactor for NF-κB activity [27], which was consistent with our observation that both the total form and phosphorylation form of NF-κB were inhibited in TPh A-PM co-treated HPF models (Supplementary Figure 2). Our results revealed the expression SREBP1 were downregulated in a dose-dependent manner in Tph A-PM co-treated HPF models (Figure 4E), which supported the positive feedback loop between SREBP1 and NF-κB quaternary complexes (Figure 6A).

Several studies indicate that NLRP3 inflammasome activation played a key role in PM-induced lung disease, mediated by NF-κB [10]. However in our study, Tph A, reducing the binding affinity between PIR and Bcl3 in NF-κB quaternary complexes, upregulated NLRP3 protein expression in a dose-dependent manner in PM-treated HPF models, which suggested that NLRP3 inflammasome promoted PM-induced adverse health events without dependence on NF-κB quaternary complexes [23]. Furthermore, *in vivo* evidence from translational studies indicated that the dysregulation of SIRT1-SREBP-NLRP3 inflammasome may contribute to atherosclerosis [17]. Based on previous evidence and on our results, SIRT1-SREBP1-inflammasomes is another plausible axis in response to PM.

Exposure to PM was responsible for high mortality. Of the number of global deaths in specific disease categories, COPD was attributed as the leading cause of death by air pollution and is already the 3^rd^ leading cause of deaths worldwide [43]. COPD is characterized by predominantly pulmonary infiltration inflammatory cells, including macrophages, neutrophils, and T lymphocytes [15]. These inflammatory cells, together with structure cells, including epithelial and endothelial cells and fibroblasts, release multiple inflammatory mediators, including chemokines, inflammasomes, cytokines, free radicles, lipid mediators, protease, and growth factors, amplifying the inflammatory cascade and triggering airway remodeling [44]. Lung function tests assess the severity of disease in COPD. However, biomarkers have been sought for diagnosis, disease progression, exacerbation risk, and therapeutic response [45]. In 2009, SIN and VESTBO reviewed the current candidate plasma or serum biomarkers linked to COPD and found that the systematic inflammatory mediators most associated with COPD outcomes were fibrinogen and C-reactive protein (CRP) [46]. There have been further conflicting reports in large COPD cohorts [47]. IL-6, leading to a rise in plasma fibrinogen and CRP, was significantly raised in COPD and elevated during exacerbations in the Evaluation of COPD Longitudinally to Identify Surrogate Endpoints (ECLIPSE) cohort [48]. Conversely, our quantitative RT-PCR results showed that *IL-6* responded only in a dose-dependent manner for 6 hours and in a time-dependent manner at low dose (Figure 2C), while our *in silico* analysis showed that *IL-6* had no significant association with COPD (Figure 5D, *P* = 0.3742) (GSE5060, n = 38). Ilumets et al. reported that matrix metalloproteinases-9 (MMP-9) in induced sputum and plasma appeared to be overexpressed in smokers [49]. However, in humans, steroid therapy showed no effect on MMP-9 levels [50]. Ilumets et al. also mentioned that MMP-8 in the sputum distinguished individuals with asymptomatic COPD, at risk of developing COPD, from healthy smokers, while Sng et al. reported a negative correlation between MMP-8 with exhaled breath condensate in COPD patients when compared to healthy controls [49, 51]. Several clinical trials demonstrated that higher blood eosinophil counts may present increased exacerbation frequency and predict better medicine response [15]. Ichikawa et al. indicated that SIRT1 decreased in lung tissue coupled with inflammatory cell infiltration, eosinophilia, and cytokine production in an asthmatic mouse model [52]. Together, PIR may serve as a sensitive biomarker for early stage cardiopulmonary disease elicited by COPD and a predictor for medicine response. However, the importance and specific role of SIRT1 in modulating eosinophil activity remains unidentified. Additionally, further studies are needed to clarify if PIR could be a biomarker for assessing the outcome of COPD.

In summary, our studies demonstrate that SIRT1 modulates the SREBP1-PIR/NLRP3 inflammasome axis in response to PM, indicating that the SIRT1-SREBP1-PIR/ NLRP3 inflammasome axis may be associated to adverse health issues. SIRT1 functions as a protector from PM exposure, whereas PIR acts as a predictor of PM-induced cardiopulmonary disease. The SIRT1-SREBP1-PIR/ NLRP3 inflammasome axis may present an attractive therapeutic target for PM-related adverse health events.

## MATERIALS AND METHODS

### PM

We purchased urban dust from Urban dust (1649b, NIST® SRM®, Gaithersburg, MD, USA). The dried PM samples were diluted with sterilized phosphate-buVered saline solution (PBS, pH 7.2) and then frozen at −80 C prior to use.

### PM treatment

After reviewing the literature [53], we chose 5 μM PM as low dose and 50 μM as high dose. HPF was incubated with PM 5 μM, 50 μM, or sham for 6 hours and 24 hours. We estimated the global RNA expression for extra 6 hours, which is the optimal time point at which mRNAs and genes are fully transcribed by PM treatments. After PM incubation for 6 or 24 hours, we extracted HPF RNA and performed Affymetrix u133 plus 2.0 cDNA microarrays to detect the global and pre-defined gene and mRNA probe changes at the specific time points.

### Cell culture and reagents

Human pulmonary fibroblasts (HPF) were purchased from ScienCell research laboratories (Cat. #3300, ScienCell, Carlsbad CA, USA). They were grown in fibroblast medium (FM, Cat. #2301, ScienCell, Carlsbad CA, USA) and stored at 37 °C and 5% CO_2_. The medium was supplemented with 2% fetal bovine serum (FBS; Cat. #0010, ScienCell, Carlsbad CA, USA), 1% growth supplement (FGS; Cat. #2352, ScienCell, Carlsbad CA, USA) and 1% penicillin/streptomycin solution (P/S; Cat. #0503, ScienCell, Carlsbad CA, USA).

### Reagents and compounds

Reagents used for the *in vitro* culture system include Ex527 (Cat. #E7034, Sigma-Aldrich®, Deisenhofen, Germany), PF429242 (Cat. #3354, Tocris Bioscience, Bristol, UK), TPh A (Cat. #TPh A ab144376, Abcam®, Cambridge, MA, USA), and Human Pirin ELISA Kit (Cat. #MBS280347, MyBioSource, San Diego CA, USA).

### RT-qPCR

Total RNA was extracted using TRIzol reagent (Invitrogen, Carlsbad, CA) according to the manufacturer’s instruction. The RNA was quantified by NanoDrop (Thermo Fisher Scientific, Waltham, USA). Reverse transcription PCR (RT-PCR) and nested RT-PCR amplification were performed with a SuperScript III kit (Invitrogen, Carlsbad, CA) according to the manufacturer’s instruction. The expression level of *Glyceraldehyde 3-phosphate dehydrogenase (GAPDH)* was predominantly used as an internal standard for RT-PCR. The specific primer sequences are as follows: *SREBP1* forward is 5′–GCAAGGCCATCGACTACATT-3′ and reverse is 5’-GGTCAGTGTGTCCTCCACCT-3′, *C-X-C motif chemokine ligand 8 (CXCL8)* forward is 5′-ATGACTTCCAAGCTGGCCGTGGCT-3′ and reverse is 5′-TCTCAGCCCTCTTCAAAAACTTCTC-3′, *interleukin 6* (*IL-6*) forward is 5′-GGTACATCCTCGACGGCATCT-3′ and reverse is 5′-GCCTCTTTGCTGCTTTCAC-3′, *Nod-like receptor protein 3 (NLRP3)* forward is 5′-CTTCTCTGATGAGGCCCAAG-3′ and reverse is 5′- GCAGCAAACTGGAAAGGAAG-3′, *interleukin 24 (IL-24*) forward is 5′-TGTGAAAGACACTATGCAAGCTC-3′ and reverse is 5′-GTGACACGATGAGAACAAAGTTG-3′, *Interleukin 6 Family Cytokine (LIF)* forward is 5′-CCAACGTGACGGACTTCCC-3′ and reverse is 5′-TACACGACTATGCGGTACAGC-3′, and *PIR* forward is 5′-GAGCAGTCGGAAGGGGTTG-3′ and reverse is 5′-TTAACTCGGGTCTGCCAATGC-3′.

### Western-blot analysis

Western blot analysis was performed with primary anti-PIR antibody (GeneTex, Irvine CA, USA), anti-NLRP3 antibody (GeneTex, Irvine, CA, USA), anti-SREBP1 antibody (GeneTex, Irvine CA, USA), anti-IL-6 antibody (Abcam, Cambridge MA, USA), anti-IL-1β antibody (GeneTex, Irvine, CA, USA), anti-SIRT1 antibody (Proteintech®, Rosemont, USA), and anti-β-actin antibody (Sigma-Aldrich, St. Louis, MO, USA). The bands corresponding to western blot were quantified using ImageJ.

### RNA microarray analysis

Total RNA was corresponding extracted and purified from HPF samples via RNeasy Mini kit (Qiagen®, Valencia CA, USA) as the initial material and then qualified by Agilent model 2100 Bioanalyzer (Agilent Technologies, Palo Alto CA, USA). All RNA samples were labeled with the GeneChip 3′IVT Express Kit (Affymetrix, Santa Clara CA, USA), hybridized, washed, and scanned to the arrays with GeneAtlas Hybridization, Wash, and Stain Kit (Affymetrix, Santa Clara CA, USA), as described by the manufacturer. Normalization genes were identically represented on the GeneChip Human Genome U133 Plus 2.0 Array (Affymetrix, Santa Clara CA, USA). Expression of genes was then generated after log2 transformation and normalization with GeneSpring software (Agilent Technologies, Palo Alto CA, USA). To uncover the significance of genes for further analysis, cutoff values for gene expression results greater than ± 1.5-fold-change were further overlaid onto Ingenuity Pathway Analysis (IPA; QIAGEN, Valencia, CA, USA) to estimate canonical pathways, downstream effects, and potential upstream regulators. in response to PM.

### *In vivo* model

6-8-week-old nonobese diabetic/severe combined immunodeficient gamma (NOD.Cg-*Prkdc^scid^ Il2rg^tm1Wjl^*/SzJ, NOD-SCID γ, NSG™, JAX®, Harbor ME, USA) male mice (20-25 g body weight) and B6.Cg-*Lep* ^ob^/J (B6 ob JAX®, Harbor ME, USA) were used. To establish *in vivo* PM model, PM particles were diluted with 10 μl of PBS and then intratracheally administered into the pulmonary regions with Hamilton syringe. Body weight and behavior were monitored weekly.

### Immunohistochemistry staining analysis

Briefly, tissue sections (5 μm) were dewaxed and rehydrated. The slides were incubated in 10 mmol/L citric buffer (pH 6.0). Antigen retrieval was done in Tris-EDTA buffer (pH 9.0) and microwaved for 15 minutes. Endogenous peroxidase activity was blocked by placing the sections in a methanol bath containing 0.5% H_2_O_2_. After blocking as above, the slides were incubated with primary antibody against target proteins, followed by biotin-conjugated secondary antibody polymer-HRP reagent The endogenous peroxidase activity was then visualized with diaminobenzidine tetrahydroxychloride (DAB) solution.

### Public database

Analysis of Gene Expression Omnibus (GEO), a public cohort microarray database, was used to validate the correlation between expression of biomarkers and PM. The differentially expressed genes in the dataset were determined by nonparametric and *t*-test analysis.

### Statistical analysis

Statistics analysis was performed with SPSS 17.0 software for Windows (SPSS, Chicago, Illinois, USA). *P* < 0.05 on a two-sided test was considered significant.

### Ethics approval

Animal care and experiments were performed in compliance with 3R principles and approved by the Academia Sinica Institutional Animal Care and Utilization Committee (IACUC).

## Supplementary Material

Supplementary Figures

Supplementary Table 1
