# Supplementary material for: Ambient particulate matter attenuates Sirtuin1 and augments SREBP1-PIR axis to induce human pulmonary fibroblast inflammation: molecular mechanism of microenvironment associated with COPD
Source: Aging (Albany NY). 2019 Jul 12;11(13):4654–71. doi: 10.18632/aging.102077 (PMC6660058; doi:10.18632/aging.102077)
Supplement: Supplementary Figures [file aging-11-102077-s001.pdf]

SUPPLEMENTARY FIGURES

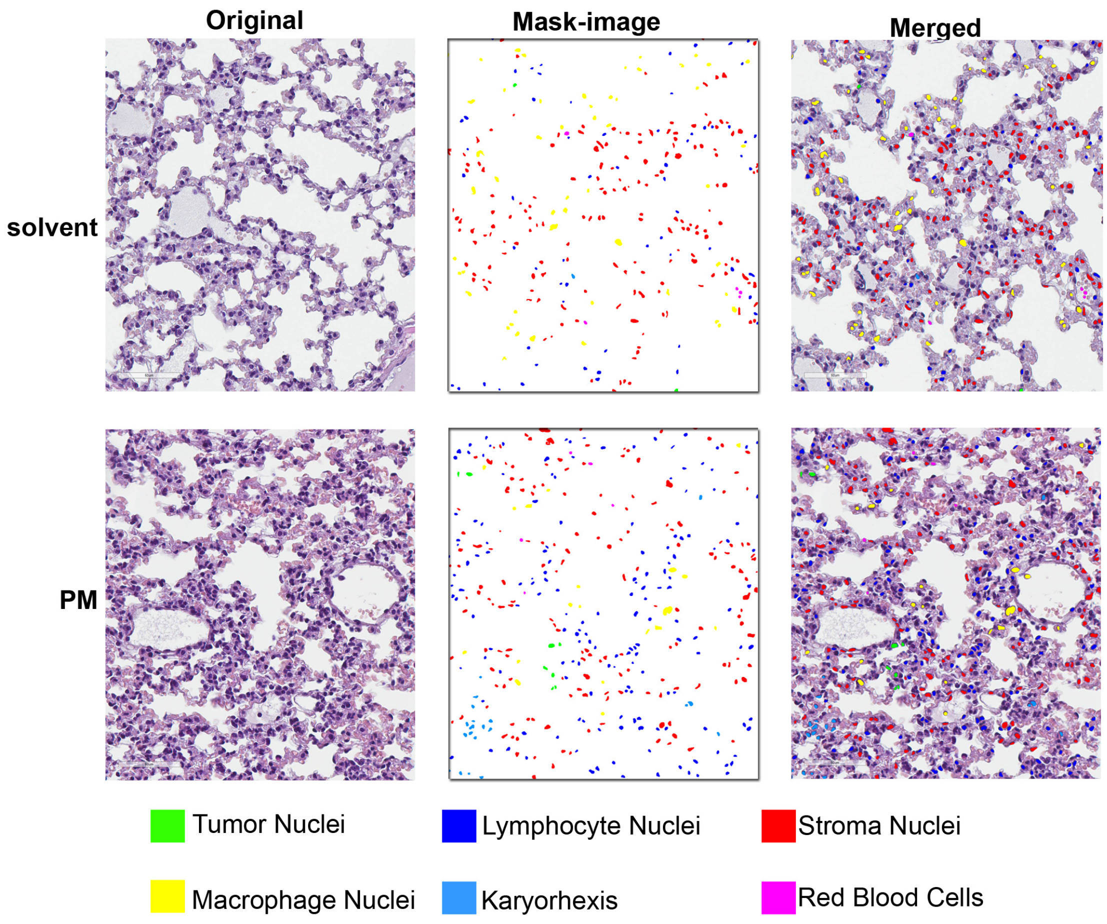

Supplementary Figure 1. PM induces adverse health events via multi-factorial mechanisms: Utilized Hematoxylin/Eosin staining of mice lung through Mask-RCNN based pathology image training model combined with deep-learning algorithm, PM-fed groups recruit more lymphocytes (blue) and stroma cell (red) than solvent control.

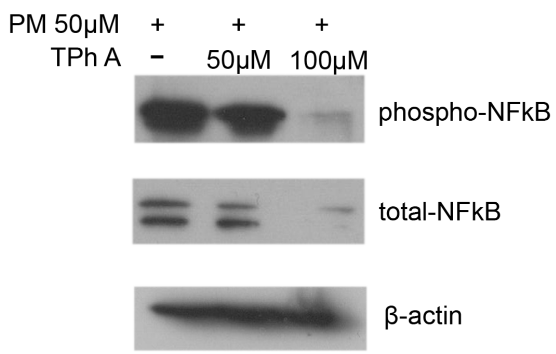

Supplementary Figure 2. NF-κB is negtively correlated with TPh A. Western blot analysis of phosphor-NFκB and total-NFκB in TPh A-PM co-treated HPF models.
