# Supplementary material for: Ambient particulate matter attenuates Sirtuin1 and augments SREBP1-PIR axis to induce human pulmonary fibroblast inflammation: molecular mechanism of microenvironment associated with COPD
Source: Aging (Albany NY). 2019 Jul 12;11(13):4654–71. doi: 10.18632/aging.102077 (PMC6660058; doi:10.18632/aging.102077)
Supplement: Supplementary Table 1 [file aging-11-102077-s002.doc]

| Probe Set ID | FC ([HPF low 6hr.CEL] vs [HPF control.CEL]) | Log FC ([HPF low 6hr.CEL] vs [HPF control.CEL]) | FC (abs) ([HPF low 6hr.CEL] vs [HPF control.CEL]) | Regulation ([HPF low 6hr.CEL] vs [HPF control.CEL]) | FC ([HPF low 24hr.CEL] vs [HPF control.CEL]) | Log FC ([HPF low 24hr.CEL] vs [HPF control.CEL]) | FC (abs) ([HPF low 24hr.CEL] vs [HPF control.CEL]) | Regulation ([HPF low 24hr.CEL] vs [HPF control.CEL]) | FC ([HPF high 6hr.CEL] vs [HPF control.CEL]) | Log FC ([HPF high 6hr.CEL] vs [HPF control.CEL]) | FC (abs) ([HPF high 6hr.CEL] vs [HPF control.CEL]) | Regulation ([HPF high 6hr.CEL] vs [HPF control.CEL]) | FC ([HPF high 24hr.CEL] vs [HPF control.CEL]) | Log FC ([HPF high 24hr.CEL] vs [HPF control.CEL]) | FC (abs) ([HPF high 24hr.CEL] vs [HPF control.CEL]) | Regulation ([HPF high 24hr.CEL] vs [HPF control.CEL]) | HPF control.CEL(normalized) | HPF low 6hr.CEL(normalized) | HPF low 24hr.CEL(normalized) | HPF high 6hr.CEL(normalized) | HPF high 24hr.CEL(normalized) | Gene Symbol |
| --- | --- | --- | --- | --- | --- | --- | --- | --- | --- | --- | --- | --- | --- | --- | --- | --- | --- | --- | --- | --- | --- | --- |
| AFFX-HUMRGE/M10098_5_at | 1.263789 | 0.337755 | 1.263789 | up | 1.399161 | 0.484561 | 1.399161 | up | 1.644649 | 0.71778 | 1.644649 | up | 1.473109 | 0.558864 | 1.473109 | up | 0 | 0.337755 | 0.484561 | 0.71778 | 0.558864 |  |
| AFFX-HUMRGE/M10098_M_at | 1.329573 | 0.410963 | 1.329573 | up | 1.347207 | 0.429972 | 1.347207 | up | 1.860881 | 0.895986 | 1.860881 | up | 1.368356 | 0.452444 | 1.368356 | up | 0 | 0.410963 | 0.429972 | 0.895986 | 0.452444 |  |
| AFFX-M27830_5_at | 1.307522 | 0.386835 | 1.307522 | up | 1.440084 | 0.526153 | 1.440084 | up | 1.604585 | 0.6822 | 1.604585 | up | 1.42872 | 0.514723 | 1.42872 | up | 0 | 0.386835 | 0.526153 | 0.6822 | 0.514723 |  |
| 160020_at | 1.253508 | 0.325972 | 1.253508 | up | 1.369936 | 0.454108 | 1.369936 | up | 1.366849 | 0.450853 | 1.366849 | up | 1.546831 | 0.629315 | 1.546831 | up | 0 | 0.325972 | 0.454108 | 0.450853 | 0.629315 | MMP14 |
| 200730_s_at | 1.554906 | 0.636827 | 1.554906 | up | 1.006 | 0.008631 | 1.006 | up | -1.19428 | -0.25614 | 1.194276 | down | -1.38254 | -0.46732 | 1.382543 | down | 0 | 0.636827 | 0.008631 | -0.25614 | -0.46732 | PTP4A1 |
| 200796_s_at | 1.272989 | 0.34822 | 1.272989 | up | 1.817307 | 0.861802 | 1.817307 | up | 1.545283 | 0.627871 | 1.545283 | up | 1.767928 | 0.82206 | 1.767928 | up | 0 | 0.34822 | 0.861802 | 0.627871 | 0.82206 | MCL1 |
| 200835_s_at | 1.208698 | 0.273453 | 1.208698 | up | 1.304667 | 0.383681 | 1.304667 | up | 1.576809 | 0.657008 | 1.576809 | up | 1.14302 | 0.192851 | 1.14302 | up | 0 | 0.273453 | 0.383681 | 0.657008 | 0.192851 | MAP4 |
| 200952_s_at | -1.03294 | -0.04676 | 1.032945 | down | 1.026138 | 0.037225 | 1.026138 | up | 1.511688 | 0.59616 | 1.511688 | up | 1.041706 | 0.058948 | 1.041706 | up | 0 | -0.04676 | 0.037225 | 0.59616 | 0.058948 | CCND2 |
| 201044_x_at | 1.296079 | 0.374154 | 1.296079 | up | 1.577016 | 0.657197 | 1.577016 | up | 1.651121 | 0.723445 | 1.651121 | up | 1.729905 | 0.790693 | 1.729905 | up | 0 | 0.374154 | 0.657197 | 0.723445 | 0.790693 | DUSP1 |
| 201050_at | 1.240963 | 0.311461 | 1.240963 | up | 1.419062 | 0.504938 | 1.419062 | up | 1.417725 | 0.503578 | 1.417725 | up | 2.036374 | 1.026002 | 2.036374 | up | 0 | 0.311461 | 0.504938 | 0.503578 | 1.026002 | PLD3 |
| 201055_s_at | 1.249485 | 0.321334 | 1.249485 | up | 1.213676 | 0.279383 | 1.213676 | up | 1.571066 | 0.651744 | 1.571066 | up | 1.074148 | 0.103192 | 1.074148 | up | 0 | 0.321334 | 0.279383 | 0.651744 | 0.103192 | HNRNPA0 |
| 201072_s_at | 1.434418 | 0.520466 | 1.434418 | up | 1.173998 | 0.23143 | 1.173998 | up | 1.766605 | 0.82098 | 1.766605 | up | 1.331943 | 0.413533 | 1.331943 | up | 0 | 0.520466 | 0.23143 | 0.82098 | 0.413533 | SMARCC1 |
| 201123_s_at | 1.134795 | 0.182432 | 1.134795 | up | 1.442906 | 0.528977 | 1.442906 | up | 1.403065 | 0.488582 | 1.403065 | up | 1.658015 | 0.729457 | 1.658015 | up | 0 | 0.182432 | 0.528977 | 0.488582 | 0.729457 | EIF5A |
| 201163_s_at | -1.39623 | -0.48154 | 1.396234 | down | -1.1333 | -0.18053 | 1.133302 | down | -1.16273 | -0.21752 | 1.162733 | down | -1.50296 | -0.5878 | 1.502958 | down | 0 | -0.48154 | -0.18053 | -0.21752 | -0.5878 | IGFBP7 |
| 201283_s_at | 1.437397 | 0.523459 | 1.437397 | up | 1.327311 | 0.408506 | 1.327311 | up | 1.327311 | 0.408506 | 1.327311 | up | 1.607517 | 0.684834 | 1.607517 | up | 0 | 0.523459 | 0.408506 | 0.408506 | 0.684834 | TRAK1 |
| 201367_s_at | 1.358631 | 0.442153 | 1.358631 | up | 1.585345 | 0.664797 | 1.585345 | up | 1.585345 | 0.664797 | 1.585345 | up | 1.985696 | 0.989645 | 1.985696 | up | 0 | 0.442153 | 0.664797 | 0.664797 | 0.989645 | ZFP36L2 |
| 201373_at | 1.214082 | 0.279866 | 1.214082 | up | 1.306759 | 0.385993 | 1.306759 | up | 1.35589 | 0.43924 | 1.35589 | up | 1.833369 | 0.874497 | 1.833369 | up | 0 | 0.279866 | 0.385993 | 0.43924 | 0.874497 | PLEC |
| 201469_s_at | 1.111098 | 0.151986 | 1.111098 | up | 1.456322 | 0.542329 | 1.456322 | up | 1.224707 | 0.292437 | 1.224707 | up | 1.63064 | 0.705438 | 1.63064 | up | 0 | 0.151986 | 0.542329 | 0.292437 | 0.705438 | SHC1 |
| 201545_s_at | 1.099795 | 0.137234 | 1.099795 | up | 1.466963 | 0.552833 | 1.466963 | up | 1.364105 | 0.447954 | 1.364105 | up | 1.517718 | 0.601904 | 1.517718 | up | 0 | 0.137234 | 0.552833 | 0.447954 | 0.601904 | BCL2L2-PABPN1///PABPN1 |
| 201551_s_at | 1.254648 | 0.327283 | 1.254648 | up | 1.355182 | 0.438486 | 1.355182 | up | 1.417801 | 0.503655 | 1.417801 | up | 1.722166 | 0.784225 | 1.722166 | up | 0 | 0.327283 | 0.438486 | 0.503655 | 0.784225 | LAMP1 |
| 201566_x_at | -1.14672 | -0.19751 | 1.146721 | down | -1.04009 | -0.05671 | 1.040093 | down | -1.22679 | -0.29489 | 1.226793 | down | -1.52474 | -0.60856 | 1.524737 | down | 0 | -0.19751 | -0.05671 | -0.29489 | -0.60856 | ID2 |
| 201679_at | 1.247459 | 0.318992 | 1.247459 | up | 1.325171 | 0.406178 | 1.325171 | up | 1.305849 | 0.384988 | 1.305849 | up | 1.532336 | 0.615733 | 1.532336 | up | 0 | 0.318992 | 0.406178 | 0.384988 | 0.615733 | SRRT |
| 201747_s_at | 1.28375 | 0.360364 | 1.28375 | up | 1.298962 | 0.377359 | 1.298962 | up | 1.413659 | 0.499434 | 1.413659 | up | 1.535334 | 0.618552 | 1.535334 | up | 0 | 0.360364 | 0.377359 | 0.499434 | 0.618552 | SAFB |
| 201750_s_at | 1.295155 | 0.373125 | 1.295155 | up | 1.587067 | 0.666363 | 1.587067 | up | 1.444514 | 0.530584 | 1.444514 | up | 1.631334 | 0.706052 | 1.631334 | up | 0 | 0.373125 | 0.666363 | 0.530584 | 0.706052 | ECE1 |
| 201790_s_at | 1.130449 | 0.176896 | 1.130449 | up | 1.352355 | 0.435474 | 1.352355 | up | 1.184209 | 0.243924 | 1.184209 | up | 1.76641 | 0.82082 | 1.76641 | up | 0 | 0.176896 | 0.435474 | 0.243924 | 0.82082 | DHCR7 |
| 201979_s_at | 1.237257 | 0.307146 | 1.237257 | up | 1.569099 | 0.649937 | 1.569099 | up | 1.561904 | 0.643306 | 1.561904 | up | 2.026013 | 1.018643 | 2.026013 | up | 0 | 0.307146 | 0.649937 | 0.643306 | 1.018643 | PPP5C |
| 201981_at | 1.25134 | 0.323474 | 1.25134 | up | 1.276624 | 0.352333 | 1.276624 | up | 1.586684 | 0.666015 | 1.586684 | up | 1.455624 | 0.541637 | 1.455624 | up | 0 | 0.323474 | 0.352333 | 0.666015 | 0.541637 | PAPPA |
| 201983_s_at | 1.225659 | 0.293557 | 1.225659 | up | 1.088248 | 0.122007 | 1.088248 | up | 1.629763 | 0.704662 | 1.629763 | up | 1.225659 | 0.293557 | 1.225659 | up | 0 | 0.293557 | 0.122007 | 0.704662 | 0.293557 | EGFR |
| 202045_s_at | 1.470247 | 0.556059 | 1.470247 | up | 1.451395 | 0.53744 | 1.451395 | up | 1.568187 | 0.649098 | 1.568187 | up | 1.853572 | 0.890308 | 1.853572 | up | 0 | 0.556059 | 0.53744 | 0.649098 | 0.890308 | ARHGAP35 |
| 202149_at | -1.18857 | -0.24923 | 1.188571 | down | -1.18857 | -0.24923 | 1.188571 | down | 1.2957 | 0.373732 | 1.2957 | up | -1.68765 | -0.75502 | 1.687653 | down | 0 | -0.24923 | -0.24923 | 0.373732 | -0.75502 | NEDD9 |
| 202198_s_at | 1.701765 | 0.767032 | 1.701765 | up | 1.312907 | 0.392765 | 1.312907 | up | 1.547921 | 0.630332 | 1.547921 | up | 1.8747 | 0.906659 | 1.8747 | up | 0 | 0.767032 | 0.392765 | 0.630332 | 0.906659 | MTMR3 |
| 202247_s_at | -1.00371 | -0.00534 | 1.003711 | down | 1.242266 | 0.312974 | 1.242266 | up | 1.242266 | 0.312974 | 1.242266 | up | 1.819782 | 0.863765 | 1.819782 | up | 0 | -0.00534 | 0.312974 | 0.312974 | 0.863765 | MTA1 |
| 202270_at | -1.04974 | -0.07003 | 1.049741 | down | 1.154807 | 0.207651 | 1.154807 | up | 1.523565 | 0.607451 | 1.523565 | up | -1.28106 | -0.35734 | 1.281064 | down | 0 | -0.07003 | 0.207651 | 0.607451 | -0.35734 | GBP1 |
| 202290_at | 1.993013 | 0.994951 | 1.993013 | up | 1.378566 | 0.463168 | 1.378566 | up | 2.641074 | 1.401125 | 2.641074 | up | 1.709365 | 0.77346 | 1.709365 | up | 0 | 0.994951 | 0.463168 | 1.401125 | 0.77346 | PDAP1 |
| 202354_s_at | 1.291935 | 0.369534 | 1.291935 | up | 1.312431 | 0.392241 | 1.312431 | up | 1.247329 | 0.318842 | 1.247329 | up | 1.565604 | 0.646719 | 1.565604 | up | 0 | 0.369534 | 0.392241 | 0.318842 | 0.646719 | GTF2F1 |
| 202434_s_at | 1.062442 | 0.087384 | 1.062442 | up | 1.806869 | 0.853492 | 1.806869 | up | 2.122108 | 1.085498 | 2.122108 | up | 2.656435 | 1.409492 | 2.656435 | up | 0 | 0.087384 | 0.853492 | 1.085498 | 1.409492 | CYP1B1 |
| 202476_s_at | 1.083664 | 0.115918 | 1.083664 | up | 1.522399 | 0.606346 | 1.522399 | up | 1.324199 | 0.40512 | 1.324199 | up | 1.939978 | 0.95604 | 1.939978 | up | 0 | 0.115918 | 0.606346 | 0.40512 | 0.95604 | TUBGCP2 |
| 202643_s_at | -1.01168 | -0.01675 | 1.01168 | down | -1.21433 | -0.28016 | 1.21433 | down | 1.516878 | 0.601105 | 1.516878 | up | -1.30697 | -0.38623 | 1.306975 | down | 0 | -0.01675 | -0.28016 | 0.601105 | -0.38623 | TNFAIP3 |
| 202714_s_at | 1.516918 | 0.601143 | 1.516918 | up | 1.303742 | 0.382658 | 1.303742 | up | 1.303742 | 0.382658 | 1.303742 | up | 1.438821 | 0.524887 | 1.438821 | up | 0 | 0.601143 | 0.382658 | 0.382658 | 0.524887 | KIAA0391 |
| 202779_s_at | 1.102453 | 0.140717 | 1.102453 | up | 1.362708 | 0.446476 | 1.362708 | up | 1.2976 | 0.375846 | 1.2976 | up | 1.5327 | 0.616076 | 1.5327 | up | 0 | 0.140717 | 0.446476 | 0.375846 | 0.616076 | UBE2S///UBE2SP1 |
| 202827_s_at | 1.505974 | 0.590696 | 1.505974 | up | 1.747398 | 0.805208 | 1.747398 | up | 1.955199 | 0.967316 | 1.955199 | up | 2.532116 | 1.340344 | 2.532116 | up | 0 | 0.590696 | 0.805208 | 0.967316 | 1.340344 | MMP14 |
| 202840_at | 1.260997 | 0.334564 | 1.260997 | up | 1.163444 | 0.218402 | 1.163444 | up | 1.977184 | 0.983447 | 1.977184 | up | 1.105195 | 0.144301 | 1.105195 | up | 0 | 0.334564 | 0.218402 | 0.983447 | 0.144301 | TAF15 |
| 202852_s_at | 2.109667 | 1.077015 | 2.109667 | up | 1.003519 | 0.005067 | 1.003519 | up | 1.328691 | 0.410006 | 1.328691 | up | -1.14385 | -0.1939 | 1.143849 | down | 0 | 1.077015 | 0.005067 | 0.410006 | -0.1939 | AAGAB |
| 202859_x_at | -1 | 0 | 1 | down | -1 | 0 | 1 | down | 2.386292 | 1.25477 | 2.386292 | up | -1.45936 | -0.54534 | 1.459363 | down | 0 | 0 | 0 | 1.25477 | -0.54534 | CXCL8 |
| 203027_s_at | 1.126076 | 0.171304 | 1.126076 | up | 1.132345 | 0.179314 | 1.132345 | up | 1.132345 | 0.179314 | 1.132345 | up | 1.720573 | 0.782889 | 1.720573 | up | 0 | 0.171304 | 0.179314 | 0.179314 | 0.782889 | MVD |
| 203093_s_at | 1.088845 | 0.122798 | 1.088845 | up | 1.354601 | 0.437868 | 1.354601 | up | 1.241082 | 0.311598 | 1.241082 | up | 1.514076 | 0.598437 | 1.514076 | up | 0 | 0.122798 | 0.437868 | 0.311598 | 0.598437 | TIMM44 |
| 203101_s_at | 1.321648 | 0.402338 | 1.321648 | up | 1.397522 | 0.482871 | 1.397522 | up | 1.44857 | 0.534629 | 1.44857 | up | 1.650056 | 0.722515 | 1.650056 | up | 0 | 0.402338 | 0.482871 | 0.534629 | 0.722515 | MGAT2 |
| 203109_at | 1.104331 | 0.143172 | 1.104331 | up | 1.537078 | 0.620191 | 1.537078 | up | 1.388553 | 0.473582 | 1.388553 | up | 1.692178 | 0.758882 | 1.692178 | up | 0 | 0.143172 | 0.620191 | 0.473582 | 0.758882 | UBE2M |
| 203134_at | 1.978246 | 0.984221 | 1.978246 | up | 1.088114 | 0.12183 | 1.088114 | up | 1.065841 | 0.091992 | 1.065841 | up | 1.054478 | 0.07653 | 1.054478 | up | 0 | 0.984221 | 0.12183 | 0.091992 | 0.07653 | PICALM |
| 203239_s_at | 1.503291 | 0.588124 | 1.503291 | up | 1.503291 | 0.588124 | 1.503291 | up | 1.522645 | 0.606579 | 1.522645 | up | 2.19957 | 1.137222 | 2.19957 | up | 0 | 0.588124 | 0.588124 | 0.606579 | 1.137222 | CNOT3 |
| 203242_s_at | -1.06703 | -0.0936 | 1.067028 | down | -1.00268 | -0.00386 | 1.002682 | down | 1.532059 | 0.615471 | 1.532059 | up | -1 | 0 | 1 | down | 0 | -0.0936 | -0.00386 | 0.615471 | 0 | PDLIM5 |
| 203243_s_at | -1.07414 | -0.10319 | 1.074143 | down | 1.117793 | 0.160653 | 1.117793 | up | 1.752795 | 0.809657 | 1.752795 | up | 1.087957 | 0.121622 | 1.087957 | up | 0 | -0.10319 | 0.160653 | 0.809657 | 0.121622 | PDLIM5 |
| 203289_s_at | -1.01459 | -0.0209 | 1.01459 | down | 1.357418 | 0.440865 | 1.357418 | up | 1.30996 | 0.389523 | 1.30996 | up | 1.573742 | 0.654199 | 1.573742 | up | 0 | -0.0209 | 0.440865 | 0.389523 | 0.654199 | NPRL3 |
| 203381_s_at | 1.425134 | 0.511098 | 1.425134 | up | 1.946322 | 0.960751 | 1.946322 | up | 1.621598 | 0.697416 | 1.621598 | up | 1.946003 | 0.960514 | 1.946003 | up | 0 | 0.511098 | 0.960751 | 0.697416 | 0.960514 | APOE |
| 203424_s_at | -1.08766 | -0.12122 | 1.087657 | down | 1.201279 | 0.264571 | 1.201279 | up | 1.111392 | 0.152368 | 1.111392 | up | 1.672486 | 0.741994 | 1.672486 | up | 0 | -0.12122 | 0.264571 | 0.152368 | 0.741994 | IGFBP5 |
| 203472_s_at | 1.090673 | 0.125219 | 1.090673 | up | 1.171803 | 0.22873 | 1.171803 | up | 1.171803 | 0.22873 | 1.171803 | up | 1.638494 | 0.71237 | 1.638494 | up | 0 | 0.125219 | 0.22873 | 0.22873 | 0.71237 | SLCO2B1 |
| 203498_at | 1.562513 | 0.643868 | 1.562513 | up | 1.120334 | 0.163929 | 1.120334 | up | 1.120334 | 0.163929 | 1.120334 | up | -1.1246 | -0.16941 | 1.1246 | down | 0 | 0.643868 | 0.163929 | 0.163929 | -0.16941 | RCAN2 |
| 203564_at | -1.56457 | -0.64577 | 1.564571 | down | 1.078368 | 0.10885 | 1.078368 | up | -1.45728 | -0.54328 | 1.45728 | down | -1.06446 | -0.09012 | 1.064455 | down | 0 | -0.64577 | 0.10885 | -0.54328 | -0.09012 | FANCG |
| 203641_s_at | -1.58378 | -0.66337 | 1.583783 | down | -1.16834 | -0.22446 | 1.168337 | down | -1.42459 | -0.51054 | 1.424586 | down | -1.24922 | -0.32103 | 1.249219 | down | 0 | -0.66337 | -0.22446 | -0.51054 | -0.32103 | COBLL1 |
| 203665_at | 1.134838 | 0.182487 | 1.134838 | up | 1.236241 | 0.30596 | 1.236241 | up | 1.134838 | 0.182487 | 1.134838 | up | 1.629538 | 0.704463 | 1.629538 | up | 0 | 0.182487 | 0.30596 | 0.182487 | 0.704463 | HMOX1 |
| 203718_at | 1.229966 | 0.298619 | 1.229966 | up | 1.190463 | 0.251523 | 1.190463 | up | 1.277516 | 0.353342 | 1.277516 | up | 1.541222 | 0.624075 | 1.541222 | up | 0 | 0.298619 | 0.251523 | 0.353342 | 0.624075 | PNPLA6 |
| 203751_x_at | 1.047582 | 0.067063 | 1.047582 | up | 1.138193 | 0.186745 | 1.138193 | up | 1.138193 | 0.186745 | 1.138193 | up | 1.517607 | 0.601799 | 1.517607 | up | 0 | 0.067063 | 0.186745 | 0.186745 | 0.601799 | JUND |
| 203865_s_at | 1.275616 | 0.351194 | 1.275616 | up | 1.19281 | 0.254365 | 1.19281 | up | 1.512185 | 0.596634 | 1.512185 | up | 1.409184 | 0.49486 | 1.409184 | up | 0 | 0.351194 | 0.254365 | 0.596634 | 0.49486 | ADARB1 |
| 203876_s_at | -1.05145 | -0.07238 | 1.051453 | down | 1.141454 | 0.190872 | 1.141454 | up | 1.092548 | 0.127697 | 1.092548 | up | 1.551866 | 0.634004 | 1.551866 | up | 0 | -0.07238 | 0.190872 | 0.127697 | 0.634004 | MMP11 |
| 203890_s_at | 1.519539 | 0.603634 | 1.519539 | up | 1.977917 | 0.983982 | 1.977917 | up | 2.21034 | 1.144268 | 2.21034 | up | 2.539588 | 1.344595 | 2.539588 | up | 0 | 0.603634 | 0.983982 | 1.144268 | 1.344595 | DAPK3 |
| 204070_at | -1.5598 | -0.64136 | 1.559801 | down | 1.016301 | 0.023327 | 1.016301 | up | 1.081603 | 0.113171 | 1.081603 | up | 1.016301 | 0.023327 | 1.016301 | up | 0 | -0.64136 | 0.023327 | 0.113171 | 0.023327 | RARRES3 |
| 204163_at | 1.035274 | 0.050013 | 1.035274 | up | 1.287584 | 0.364666 | 1.287584 | up | 1.297449 | 0.375678 | 1.297449 | up | 1.777722 | 0.83003 | 1.777722 | up | 0 | 0.050013 | 0.364666 | 0.375678 | 0.83003 | EMILIN1 |
| 204239_s_at | -1.61146 | -0.68837 | 1.611458 | down | -1.21395 | -0.27971 | 1.213952 | down | -1.21395 | -0.27971 | 1.213952 | down | -1.17292 | -0.2301 | 1.17292 | down | 0 | -0.68837 | -0.27971 | -0.27971 | -0.2301 | NNAT |
| 204337_at | -1.24764 | -0.3192 | 1.247638 | down | -1.8499 | -0.88745 | 1.849901 | down | -1.45335 | -0.53938 | 1.453352 | down | -1.52918 | -0.61276 | 1.529178 | down | 0 | -0.3192 | -0.88745 | -0.53938 | -0.61276 | RGS4 |
| 204339_s_at | -1.00836 | -0.012 | 1.008356 | down | -1.53248 | -0.61586 | 1.532476 | down | -1.09649 | -0.13289 | 1.096492 | down | -1.09649 | -0.13289 | 1.096492 | down | 0 | -0.012 | -0.61586 | -0.13289 | -0.13289 | RGS4 |
| 204470_at | -1.13285 | -0.17996 | 1.132853 | down | -1.08354 | -0.11575 | 1.083539 | down | 1.306594 | 0.38581 | 1.306594 | up | -1.61247 | -0.68927 | 1.61247 | down | 0 | -0.17996 | -0.11575 | 0.38581 | -0.68927 | CXCL1 |
| 204548_at | -1.30636 | -0.38556 | 1.306364 | down | -1.39223 | -0.4774 | 1.39223 | down | -1.51727 | -0.60148 | 1.517271 | down | -1.33923 | -0.42141 | 1.339232 | down | 0 | -0.38556 | -0.4774 | -0.60148 | -0.42141 | STAR |
| 204567_s_at | -1.09012 | -0.12448 | 1.090115 | down | 1.73336 | 0.793571 | 1.73336 | up | 1.050253 | 0.070737 | 1.050253 | up | 1.152728 | 0.205052 | 1.152728 | up | 0 | -0.12448 | 0.793571 | 0.070737 | 0.205052 | ABCG1 |
| 204581_at | -1.13139 | -0.17809 | 1.131389 | down | -1.55628 | -0.6381 | 1.556278 | down | -1.04328 | -0.06113 | 1.043284 | down | -1.00857 | -0.01231 | 1.008566 | down | 0 | -0.17809 | -0.6381 | -0.06113 | -0.01231 | CD22 |
| 204592_at | 1.014324 | 0.020519 | 1.014324 | up | 1.381529 | 0.466266 | 1.381529 | up | 1.333426 | 0.415137 | 1.333426 | up | 1.504025 | 0.588828 | 1.504025 | up | 0 | 0.020519 | 0.466266 | 0.415137 | 0.588828 | DLG4 |
| 204614_at | -1.08553 | -0.1184 | 1.085529 | down | -1.0427 | -0.06033 | 1.042703 | down | 1.591397 | 0.670294 | 1.591397 | up | 1.035286 | 0.05003 | 1.035286 | up | 0 | -0.1184 | -0.06033 | 0.670294 | 0.05003 | SERPINB2 |
| 204673_at | -1.57343 | -0.65392 | 1.573433 | down | -1.18098 | -0.23998 | 1.180977 | down | -1.18098 | -0.23998 | 1.180977 | down | -1.18098 | -0.23998 | 1.180977 | down | 0 | -0.65392 | -0.23998 | -0.23998 | -0.23998 | MUC2 |
| 204684_at | 1.330366 | 0.411823 | 1.330366 | up | 1.488173 | 0.573543 | 1.488173 | up | 1.929416 | 0.948164 | 1.929416 | up | 1.73066 | 0.791323 | 1.73066 | up | 0 | 0.411823 | 0.573543 | 0.948164 | 0.791323 | NPTX1 |
| 204705_x_at | -1.69914 | -0.76481 | 1.699144 | down | -1.43033 | -0.51635 | 1.43033 | down | -1.69429 | -0.76068 | 1.694293 | down | -1.39793 | -0.4833 | 1.397934 | down | 0 | -0.76481 | -0.51635 | -0.76068 | -0.4833 | ALDOB |
| 204748_at | -1.00345 | -0.00496 | 1.003446 | down | -1 | 0 | 1 | down | 1.513305 | 0.597703 | 1.513305 | up | -1.1359 | -0.18384 | 1.135905 | down | 0 | -0.00496 | 0 | 0.597703 | -0.18384 | PTGS2 |
| 204769_s_at | 1.117332 | 0.160058 | 1.117332 | up | 1.162567 | 0.217314 | 1.162567 | up | 1.558708 | 0.640351 | 1.558708 | up | 1.079226 | 0.109997 | 1.079226 | up | 0 | 0.160058 | 0.217314 | 0.640351 | 0.109997 | TAP2 |
| 204792_s_at | 1.316095 | 0.396264 | 1.316095 | up | 1.116961 | 0.159579 | 1.116961 | up | 1.24822 | 0.319872 | 1.24822 | up | 1.575892 | 0.656169 | 1.575892 | up | 0 | 0.396264 | 0.159579 | 0.319872 | 0.656169 | IFT140 |
| 204897_at | -1.15118 | -0.20312 | 1.151183 | down | -1.50207 | -0.58695 | 1.502066 | down | 1.001008 | 0.001454 | 1.001008 | up | -1.74899 | -0.80652 | 1.748986 | down | 0 | -0.20312 | -0.58695 | 0.001454 | -0.80652 | PTGER4 |
| 204898_at | -1.2535 | -0.32596 | 1.253502 | down | -1.51493 | -0.59925 | 1.514932 | down | -1.49966 | -0.58463 | 1.499658 | down | -1.35791 | -0.44139 | 1.357915 | down | 0 | -0.32596 | -0.59925 | -0.58463 | -0.44139 | SAP30 |
| 204933_s_at | 1.208349 | 0.273037 | 1.208349 | up | 1.554539 | 0.636487 | 1.554539 | up | 1.333896 | 0.415647 | 1.333896 | up | 1.298758 | 0.377133 | 1.298758 | up | 0 | 0.273037 | 0.636487 | 0.415647 | 0.377133 | TNFRSF11B |
| 204935_at | -1.02901 | -0.04126 | 1.029012 | down | 1.191959 | 0.253335 | 1.191959 | up | -1.02901 | -0.04126 | 1.029012 | down | -1.54581 | -0.62836 | 1.545812 | down | 0 | -0.04126 | 0.253335 | -0.04126 | -0.62836 | PTPN2 |
| 204956_at | -1.1918 | -0.25314 | 1.191797 | down | 1.069625 | 0.097106 | 1.069625 | up | 1.027251 | 0.038789 | 1.027251 | up | -1.65092 | -0.72327 | 1.650924 | down | 0 | -0.25314 | 0.097106 | 0.038789 | -0.72327 | MTAP |
| 205195_at | -1.07204 | -0.10035 | 1.072037 | down | 1.484766 | 0.570235 | 1.484766 | up | 1.869615 | 0.902741 | 1.869615 | up | 1.74311 | 0.801664 | 1.74311 | up | 0 | -0.10035 | 0.570235 | 0.902741 | 0.801664 | AP1S1 |
| 205196_s_at | 1.026252 | 0.037385 | 1.026252 | up | 1.647182 | 0.72 | 1.647182 | up | 1.842749 | 0.881859 | 1.842749 | up | 1.815414 | 0.860298 | 1.815414 | up | 0 | 0.037385 | 0.72 | 0.881859 | 0.860298 | AP1S1 |
| 205207_at | 1.020714 | 0.029578 | 1.020714 | up | 1.250364 | 0.322349 | 1.250364 | up | 1.65761 | 0.729105 | 1.65761 | up | 1.191068 | 0.252255 | 1.191068 | up | 0 | 0.029578 | 0.322349 | 0.729105 | 0.252255 | IL6 |
| 205266_at | 1.106476 | 0.145972 | 1.106476 | up | 1.27372 | 0.349048 | 1.27372 | up | 1.903518 | 0.928668 | 1.903518 | up | 1.271459 | 0.346485 | 1.271459 | up | 0 | 0.145972 | 0.349048 | 0.928668 | 0.346485 | LIF |
| 205461_at | 1.093861 | 0.12943 | 1.093861 | up | 1.218422 | 0.285013 | 1.218422 | up | 1.208824 | 0.273604 | 1.208824 | up | 1.559919 | 0.641471 | 1.559919 | up | 0 | 0.12943 | 0.285013 | 0.273604 | 0.641471 | RAB35 |
| 205462_s_at | 1.132713 | 0.179782 | 1.132713 | up | 1.410622 | 0.496331 | 1.410622 | up | 1.246139 | 0.317465 | 1.246139 | up | 1.540451 | 0.623353 | 1.540451 | up | 0 | 0.179782 | 0.496331 | 0.317465 | 0.623353 | HPCAL1 |
| 205529_s_at | 1.497106 | 0.582177 | 1.497106 | up | 1.720135 | 0.782522 | 1.720135 | up | 1.467814 | 0.553669 | 1.467814 | up | 1.474158 | 0.559891 | 1.474158 | up | 0 | 0.582177 | 0.782522 | 0.553669 | 0.559891 | RUNX1T1 |
| 205594_at | 1.530624 | 0.61412 | 1.530624 | up | -1.09008 | -0.12443 | 1.090076 | down | -1.15276 | -0.20509 | 1.152762 | down | 1.031242 | 0.044383 | 1.031242 | up | 0 | 0.61412 | -0.12443 | -0.20509 | 0.044383 | ZNF652 |
| 205681_at | -1.16927 | -0.2256 | 1.169266 | down | -1.09969 | -0.1371 | 1.099693 | down | 1.634129 | 0.708522 | 1.634129 | up | -1.42835 | -0.51435 | 1.428346 | down | 0 | -0.2256 | -0.1371 | 0.708522 | -0.51435 | BCL2A1 |
| 205713_s_at | 1.139562 | 0.188479 | 1.139562 | up | 1.385669 | 0.470582 | 1.385669 | up | 1.363105 | 0.446897 | 1.363105 | up | 1.60944 | 0.686559 | 1.60944 | up | 0 | 0.188479 | 0.470582 | 0.446897 | 0.686559 | COMP |
| 205738_s_at | 1.221557 | 0.288721 | 1.221557 | up | 1.238473 | 0.308563 | 1.238473 | up | 1.095413 | 0.131475 | 1.095413 | up | 1.553031 | 0.635087 | 1.553031 | up | 0 | 0.288721 | 0.308563 | 0.131475 | 0.635087 | FABP3 |
| 205748_s_at | 1.090583 | 0.1251 | 1.090583 | up | 1.31753 | 0.397836 | 1.31753 | up | 1.289191 | 0.366466 | 1.289191 | up | 1.564566 | 0.645762 | 1.564566 | up | 0 | 0.1251 | 0.397836 | 0.366466 | 0.645762 | RNF126 |
| 205762_s_at | -1.09943 | -0.13675 | 1.099429 | down | -1.09761 | -0.13437 | 1.097612 | down | -1.09943 | -0.13675 | 1.099429 | down | -1.64184 | -0.71532 | 1.641844 | down | 0 | -0.13675 | -0.13437 | -0.13675 | -0.71532 | DUS4L |
| 205822_s_at | 1.276026 | 0.351657 | 1.276026 | up | 1.099788 | 0.137226 | 1.099788 | up | 1.63208 | 0.706711 | 1.63208 | up | 1.370046 | 0.454224 | 1.370046 | up | 0 | 0.351657 | 0.137226 | 0.706711 | 0.454224 | HMGCS1 |
| 205925_s_at | 1.288348 | 0.365522 | 1.288348 | up | 1.286199 | 0.363114 | 1.286199 | up | 1.541807 | 0.624622 | 1.541807 | up | 1.58276 | 0.662443 | 1.58276 | up | 0 | 0.365522 | 0.363114 | 0.624622 | 0.662443 | RAB3B |
| 206030_at | -1.27343 | -0.34872 | 1.273427 | down | -1.27343 | -0.34872 | 1.273427 | down | -1.582 | -0.66175 | 1.581999 | down | -1.27343 | -0.34872 | 1.273427 | down | 0 | -0.34872 | -0.34872 | -0.66175 | -0.34872 | ASPA |
| 206059_at | 1.259931 | 0.333344 | 1.259931 | up | 1.384811 | 0.469689 | 1.384811 | up | 1.606314 | 0.683754 | 1.606314 | up | 1.384811 | 0.469689 | 1.384811 | up | 0 | 0.333344 | 0.469689 | 0.683754 | 0.469689 | ZNF91 |
| 206103_at | 1.425456 | 0.511424 | 1.425456 | up | 1.556494 | 0.6383 | 1.556494 | up | 1.696456 | 0.762524 | 1.696456 | up | 1.655975 | 0.727681 | 1.655975 | up | 0 | 0.511424 | 0.6383 | 0.762524 | 0.727681 | RAC3 |
| 206220_s_at | -1.03915 | -0.0554 | 1.039149 | down | 1.123912 | 0.168529 | 1.123912 | up | 1.123912 | 0.168529 | 1.123912 | up | 1.520307 | 0.604363 | 1.520307 | up | 0 | -0.0554 | 0.168529 | 0.168529 | 0.604363 | RASA3 |
| 206266_at | -1.64131 | -0.71484 | 1.641305 | down | -1.25216 | -0.32442 | 1.252163 | down | -1.14628 | -0.19695 | 1.146275 | down | -1.01656 | -0.02369 | 1.016558 | down | 0 | -0.71484 | -0.32442 | -0.19695 | -0.02369 | GPLD1 |
| 206331_at | -1.0298 | -0.04237 | 1.029803 | down | -1.33805 | -0.42013 | 1.338052 | down | -1.59064 | -0.66961 | 1.590644 | down | -1.38962 | -0.47469 | 1.389621 | down | 0 | -0.04237 | -0.42013 | -0.66961 | -0.47469 | CALCRL |
| 206363_at | 1.33034 | 0.411795 | 1.33034 | up | 1.614799 | 0.691355 | 1.614799 | up | 1.284132 | 0.360793 | 1.284132 | up | 1.203903 | 0.267719 | 1.203903 | up | 0 | 0.411795 | 0.691355 | 0.360793 | 0.267719 | MAF |
| 206399_x_at | -1.16549 | -0.22094 | 1.165494 | down | -1.16813 | -0.22421 | 1.168134 | down | -1.51578 | -0.60006 | 1.515778 | down | -1.07095 | -0.09889 | 1.070952 | down | 0 | -0.22094 | -0.22421 | -0.60006 | -0.09889 | CACNA1A |
| 206432_at | -1.23512 | -0.30465 | 1.235122 | down | -1.39294 | -0.47813 | 1.392941 | down | -1.17235 | -0.2294 | 1.172347 | down | -1.98856 | -0.99172 | 1.988557 | down | 0 | -0.30465 | -0.47813 | -0.2294 | -0.99172 | HAS2 |
| 206463_s_at | 1.073937 | 0.102909 | 1.073937 | up | 1.044015 | 0.062142 | 1.044015 | up | 1.097428 | 0.134127 | 1.097428 | up | 1.541832 | 0.624646 | 1.541832 | up | 0 | 0.102909 | 0.062142 | 0.134127 | 0.624646 | DHRS2 |
| 206569_at | 1.03763 | 0.053292 | 1.03763 | up | -1.35166 | -0.43474 | 1.351663 | down | 1.818155 | 0.862475 | 1.818155 | up | 1.03763 | 0.053292 | 1.03763 | up | 0 | 0.053292 | -0.43474 | 0.862475 | 0.053292 | IL24 |
| 206638_at | 1.183279 | 0.242791 | 1.183279 | up | 1.545848 | 0.628398 | 1.545848 | up | 1.153384 | 0.205873 | 1.153384 | up | 1.183279 | 0.242791 | 1.183279 | up | 0 | 0.242791 | 0.628398 | 0.205873 | 0.242791 | HTR2B |
| 206665_s_at | -1.13676 | -0.18493 | 1.136761 | down | 1.057338 | 0.080436 | 1.057338 | up | 1.024852 | 0.035416 | 1.024852 | up | 1.625943 | 0.701276 | 1.625943 | up | 0 | -0.18493 | 0.080436 | 0.035416 | 0.701276 | BCL2L1 |
| 206674_at | -1.41717 | -0.50301 | 1.417171 | down | -1.43782 | -0.52388 | 1.437818 | down | -1.51193 | -0.59639 | 1.511926 | down | -1.08326 | -0.11538 | 1.083264 | down | 0 | -0.50301 | -0.52388 | -0.59639 | -0.11538 | FLT3 |
| 206708_at | 1.511195 | 0.59569 | 1.511195 | up | 1.262566 | 0.336359 | 1.262566 | up | 1.262566 | 0.336359 | 1.262566 | up | 1.262566 | 0.336359 | 1.262566 | up | 0 | 0.59569 | 0.336359 | 0.336359 | 0.336359 | FOXN2 |
| 206824_at | 1.727071 | 0.788327 | 1.727071 | up | 1.465011 | 0.550911 | 1.465011 | up | 1.465011 | 0.550911 | 1.465011 | up | 1.465011 | 0.550911 | 1.465011 | up | 0 | 0.788327 | 0.550911 | 0.550911 | 0.550911 | CES1P1 |
| 206988_at | -1.50533 | -0.59008 | 1.505335 | down | -1.16561 | -0.22108 | 1.165609 | down | -1.16561 | -0.22108 | 1.165609 | down | -1.16561 | -0.22108 | 1.165609 | down | 0 | -0.59008 | -0.22108 | -0.22108 | -0.22108 | CCL25 |
| 207001_x_at | 1.1279 | 0.173639 | 1.1279 | up | 1.472784 | 0.558546 | 1.472784 | up | 1.173418 | 0.230717 | 1.173418 | up | 1.665899 | 0.736301 | 1.665899 | up | 0 | 0.173639 | 0.558546 | 0.230717 | 0.736301 | TSC22D3 |
| 207038_at | 1.282902 | 0.359411 | 1.282902 | up | 1.411974 | 0.497714 | 1.411974 | up | 1.895212 | 0.922359 | 1.895212 | up | 1.500509 | 0.585452 | 1.500509 | up | 0 | 0.359411 | 0.497714 | 0.922359 | 0.585452 | SLC16A6 |
| 207057_at | 1.353353 | 0.436538 | 1.353353 | up | 1.340969 | 0.423275 | 1.340969 | up | 1.573514 | 0.65399 | 1.573514 | up | 1.161025 | 0.215399 | 1.161025 | up | 0 | 0.436538 | 0.423275 | 0.65399 | 0.215399 | SLC16A7 |
| 207077_at | 1.063717 | 0.089114 | 1.063717 | up | -1.09579 | -0.13197 | 1.095791 | down | -1.05835 | -0.08182 | 1.058352 | down | 1.501816 | 0.586708 | 1.501816 | up | 0 | 0.089114 | -0.13197 | -0.08182 | 0.586708 | CELA2B |
| 207096_at | -1.20938 | -0.27427 | 1.209379 | down | -1.54584 | -0.62839 | 1.545839 | down | -1.19622 | -0.25848 | 1.196219 | down | -1.19622 | -0.25848 | 1.196219 | down | 0 | -0.27427 | -0.62839 | -0.25848 | -0.25848 | SAA4 |
| 207219_at | -1.12276 | -0.16705 | 1.122758 | down | -1.0184 | -0.02631 | 1.018405 | down | -1.12276 | -0.16705 | 1.122758 | down | -1.54473 | -0.62736 | 1.544735 | down | 0 | -0.16705 | -0.02631 | -0.16705 | -0.62736 | ZFP69B |
| 207302_at | 1.138668 | 0.187346 | 1.138668 | up | 1.538721 | 0.621731 | 1.538721 | up | 1.274108 | 0.349487 | 1.274108 | up | 1.290121 | 0.367507 | 1.290121 | up | 0 | 0.187346 | 0.621731 | 0.349487 | 0.367507 | SGCG |
| 207398_at | -1.22183 | -0.28904 | 1.221827 | down | -1.22183 | -0.28904 | 1.221827 | down | -1.6183 | -0.69448 | 1.618303 | down | -1.22183 | -0.28904 | 1.221827 | down | 0 | -0.28904 | -0.28904 | -0.69448 | -0.28904 | HOXD13 |
| 207469_s_at | 1.194884 | 0.256871 | 1.194884 | up | 1.194884 | 0.256871 | 1.194884 | up | 1.240681 | 0.311132 | 1.240681 | up | 1.519544 | 0.603638 | 1.519544 | up | 0 | 0.256871 | 0.256871 | 0.311132 | 0.603638 | PIR |
| 207522_s_at | -1.03222 | -0.04575 | 1.032218 | down | 1.515733 | 0.600016 | 1.515733 | up | 1.478409 | 0.564045 | 1.478409 | up | 2.267291 | 1.180969 | 2.267291 | up | 0 | -0.04575 | 0.600016 | 0.564045 | 1.180969 | ATP2A3 |
| 207528_s_at | 1.274917 | 0.350403 | 1.274917 | up | 1.30047 | 0.379034 | 1.30047 | up | 1.646344 | 0.719265 | 1.646344 | up | 1.31534 | 0.395435 | 1.31534 | up | 0 | 0.350403 | 0.379034 | 0.719265 | 0.395435 | SLC7A11 |
| 207886_s_at | 1.551367 | 0.63354 | 1.551367 | up | -1.08602 | -0.11905 | 1.086022 | down | -1.07254 | -0.10104 | 1.072545 | down | 1.181338 | 0.240422 | 1.181338 | up | 0 | 0.63354 | -0.11905 | -0.10104 | 0.240422 | CALCR |
| 208038_at | -1.00868 | -0.01246 | 1.008675 | down | 1.552391 | 0.634492 | 1.552391 | up | -1 | 0 | 1 | down | -1.07145 | -0.09957 | 1.071451 | down | 0 | -0.01246 | 0.634492 | 0 | -0.09957 | IL1RL2 |
| 208167_s_at | 1.272018 | 0.347119 | 1.272018 | up | 1.500927 | 0.585854 | 1.500927 | up | 1.081884 | 0.113545 | 1.081884 | up | 1.081884 | 0.113545 | 1.081884 | up | 0 | 0.347119 | 0.585854 | 0.113545 | 0.113545 | MMP16 |
| 208224_at | -1.225 | -0.29279 | 1.225004 | down | -1.59887 | -0.67705 | 1.598867 | down | -1.20535 | -0.26945 | 1.205347 | down | 1.052624 | 0.07399 | 1.052624 | up | 0 | -0.29279 | -0.67705 | -0.26945 | 0.07399 | HOXB1 |
| 208378_x_at | -1.09709 | -0.13369 | 1.097093 | down | -1.30486 | -0.38389 | 1.304856 | down | 1.204241 | 0.268124 | 1.204241 | up | -1.61376 | -0.69043 | 1.61376 | down | 0 | -0.13369 | -0.38389 | 0.268124 | -0.69043 | FGF5 |
| 208394_x_at | -1.24719 | -0.31868 | 1.247188 | down | -1.30465 | -0.38366 | 1.304645 | down | 1.118919 | 0.162105 | 1.118919 | up | -1.9868 | -0.99045 | 1.986798 | down | 0 | -0.31868 | -0.38366 | 0.162105 | -0.99045 | ESM1 |
| 208478_s_at | 1.64504 | 0.718123 | 1.64504 | up | 1.343529 | 0.426028 | 1.343529 | up | 1.336333 | 0.41828 | 1.336333 | up | 1.262562 | 0.336354 | 1.262562 | up | 0 | 0.718123 | 0.426028 | 0.41828 | 0.336354 | BAX |
| 208610_s_at | 1.132534 | 0.179554 | 1.132534 | up | 1.271576 | 0.346618 | 1.271576 | up | 1.775015 | 0.827831 | 1.775015 | up | 1.578235 | 0.658312 | 1.578235 | up | 0 | 0.179554 | 0.346618 | 0.827831 | 0.658312 | SRRM2 |
| 208958_at | -1.16791 | -0.22393 | 1.167913 | down | -1.12346 | -0.16795 | 1.123463 | down | -1.04519 | -0.06377 | 1.045191 | down | -1.74932 | -0.80679 | 1.749321 | down | 0 | -0.22393 | -0.16795 | -0.06377 | -0.80679 | ERP44 |
| 208978_at | 1.150385 | 0.202116 | 1.150385 | up | 1.378565 | 0.463167 | 1.378565 | up | 1.195245 | 0.257307 | 1.195245 | up | 1.573364 | 0.653852 | 1.573364 | up | 0 | 0.202116 | 0.463167 | 0.257307 | 0.653852 | CRIP2 |
| 208987_s_at | 1.3949 | 0.480162 | 1.3949 | up | 1.502568 | 0.58743 | 1.502568 | up | 1.3949 | 0.480162 | 1.3949 | up | 1.3949 | 0.480162 | 1.3949 | up | 0 | 0.480162 | 0.58743 | 0.480162 | 0.480162 | KDM2A |
| 209013_x_at | 1.357474 | 0.440925 | 1.357474 | up | 1.350695 | 0.433702 | 1.350695 | up | 1.473585 | 0.55933 | 1.473585 | up | 1.561689 | 0.643107 | 1.561689 | up | 0 | 0.440925 | 0.433702 | 0.55933 | 0.643107 | TRIO |
| 209113_s_at | 1.176351 | 0.234319 | 1.176351 | up | 1.362627 | 0.446391 | 1.362627 | up | 1.263529 | 0.337459 | 1.263529 | up | 1.606121 | 0.683581 | 1.606121 | up | 0 | 0.234319 | 0.446391 | 0.337459 | 0.683581 | HMG20B |
| 209151_x_at | 1.416719 | 0.502553 | 1.416719 | up | 1.449023 | 0.535081 | 1.449023 | up | 1.449023 | 0.535081 | 1.449023 | up | 1.769183 | 0.823083 | 1.769183 | up | 0 | 0.502553 | 0.535081 | 0.535081 | 0.823083 | TCF3 |
| 209156_s_at | 1.182179 | 0.241448 | 1.182179 | up | 1.314132 | 0.39411 | 1.314132 | up | 1.271407 | 0.346426 | 1.271407 | up | 1.615903 | 0.692341 | 1.615903 | up | 0 | 0.241448 | 0.39411 | 0.346426 | 0.692341 | COL6A2 |
| 209169_at | 1.304607 | 0.383615 | 1.304607 | up | 1.304607 | 0.383615 | 1.304607 | up | 1.526573 | 0.610297 | 1.526573 | up | -1.1055 | -0.1447 | 1.105504 | down | 0 | 0.383615 | 0.383615 | 0.610297 | -0.1447 | GPM6B |
| 209184_s_at | 1.230381 | 0.299105 | 1.230381 | up | 1.215498 | 0.281548 | 1.215498 | up | 1.578502 | 0.658556 | 1.578502 | up | 1.318189 | 0.398558 | 1.318189 | up | 0 | 0.299105 | 0.281548 | 0.658556 | 0.398558 | IRS2 |
| 209260_at | -1.10431 | -0.14314 | 1.104307 | down | -1.59097 | -0.66991 | 1.590971 | down | -1.25892 | -0.33219 | 1.258925 | down | -1.15222 | -0.20442 | 1.152222 | down | 0 | -0.14314 | -0.66991 | -0.33219 | -0.20442 | SFN |
| 209395_at | 1.250326 | 0.322304 | 1.250326 | up | 1.71666 | 0.779604 | 1.71666 | up | 1.570576 | 0.651293 | 1.570576 | up | 1.412192 | 0.497936 | 1.412192 | up | 0 | 0.322304 | 0.779604 | 0.651293 | 0.497936 | CHI3L1 |
| 209428_s_at | 1.077778 | 0.10806 | 1.077778 | up | 1.20733 | 0.27182 | 1.20733 | up | 1.20733 | 0.27182 | 1.20733 | up | 1.679687 | 0.748193 | 1.679687 | up | 0 | 0.10806 | 0.27182 | 0.27182 | 0.748193 | ZFPL1 |
| 209456_s_at | 1.801785 | 0.849427 | 1.801785 | up | 1.462051 | 0.547993 | 1.462051 | up | 1.521009 | 0.605028 | 1.521009 | up | 1.462051 | 0.547993 | 1.462051 | up | 0 | 0.849427 | 0.547993 | 0.605028 | 0.547993 | FBXW11 |
| 209612_s_at | 1.013279 | 0.019032 | 1.013279 | up | 1.731681 | 0.792173 | 1.731681 | up | 1.289685 | 0.367019 | 1.289685 | up | 1.020316 | 0.029016 | 1.020316 | up | 0 | 0.019032 | 0.792173 | 0.367019 | 0.029016 | ADH1B |
| 209613_s_at | -1.01347 | -0.0193 | 1.01347 | down | 2.188342 | 1.129838 | 2.188342 | up | 1.512328 | 0.596771 | 1.512328 | up | -1.23899 | -0.30917 | 1.238995 | down | 0 | -0.0193 | 1.129838 | 0.596771 | -0.30917 | ADH1B |
| 209638_x_at | 1.562372 | 0.643738 | 1.562372 | up | 1.014386 | 0.020606 | 1.014386 | up | 1.172086 | 0.229078 | 1.172086 | up | 1.211618 | 0.276935 | 1.211618 | up | 0 | 0.643738 | 0.020606 | 0.229078 | 0.276935 | RGS12 |
| 209687_at | -1 | 0 | 1 | down | 1.381097 | 0.465815 | 1.381097 | up | -1 | 0 | 1 | down | -1.83779 | -0.87797 | 1.837785 | down | 0 | 0 | 0.465815 | 0 | -0.87797 | CXCL12 |
| 209754_s_at | 1.020578 | 0.029386 | 1.020578 | up | 1.396442 | 0.481756 | 1.396442 | up | 1.415055 | 0.500858 | 1.415055 | up | 1.541279 | 0.624128 | 1.541279 | up | 0 | 0.029386 | 0.481756 | 0.500858 | 0.624128 | TMPO |
| 209859_at | -1.94533 | -0.96002 | 1.945333 | down | -1.29257 | -0.37025 | 1.292574 | down | -1.29257 | -0.37025 | 1.292574 | down | -1.55012 | -0.63238 | 1.550115 | down | 0 | -0.96002 | -0.37025 | -0.37025 | -0.63238 | TRIM9 |
| 209979_at | 1.893263 | 0.920875 | 1.893263 | up | 1.353408 | 0.436597 | 1.353408 | up | 1.353408 | 0.436597 | 1.353408 | up | 1.413856 | 0.499635 | 1.413856 | up | 0 | 0.920875 | 0.436597 | 0.436597 | 0.499635 | ADARB1 |
| 210010_s_at | -1.01797 | -0.0257 | 1.017971 | down | 1.3161 | 0.396269 | 1.3161 | up | 1.100877 | 0.138654 | 1.100877 | up | 1.53594 | 0.619122 | 1.53594 | up | 0 | -0.0257 | 0.396269 | 0.138654 | 0.619122 | SLC25A1 |
| 210042_s_at | 1.297916 | 0.376197 | 1.297916 | up | 1.094483 | 0.130249 | 1.094483 | up | 1.7123 | 0.775935 | 1.7123 | up | 1.23478 | 0.304255 | 1.23478 | up | 0 | 0.376197 | 0.130249 | 0.775935 | 0.304255 | CTSZ |
| 210077_s_at | 1.53909 | 0.622078 | 1.53909 | up | -1.03346 | -0.04748 | 1.033457 | down | -1.18134 | -0.24042 | 1.181338 | down | -1.03532 | -0.05008 | 1.035323 | down | 0 | 0.622078 | -0.04748 | -0.24042 | -0.05008 | SRSF5 |
| 210119_at | 1.505518 | 0.59026 | 1.505518 | up | -1.23184 | -0.30081 | 1.231839 | down | 1.002843 | 0.004096 | 1.002843 | up | 1.002843 | 0.004096 | 1.002843 | up | 0 | 0.59026 | -0.30081 | 0.004096 | 0.004096 | KCNJ15 |
| 210175_at | -1.3559 | -0.43926 | 1.355905 | down | -1.03923 | -0.05552 | 1.039233 | down | -1.06437 | -0.09 | 1.064372 | down | -1.51909 | -0.60321 | 1.519094 | down | 0 | -0.43926 | -0.05552 | -0.09 | -0.60321 | GCFC2 |
| 210256_s_at | 1.898459 | 0.924829 | 1.898459 | up | 1.523582 | 0.607467 | 1.523582 | up | 1.940718 | 0.956591 | 1.940718 | up | 1.061853 | 0.086585 | 1.061853 | up | 0 | 0.924829 | 0.607467 | 0.956591 | 0.086585 | PIP5K1A |
| 210286_s_at | -1 | 0 | 1 | down | 1.125282 | 0.170286 | 1.125282 | up | 1.144333 | 0.194507 | 1.144333 | up | -1.5428 | -0.62556 | 1.542804 | down | 0 | 0 | 0.170286 | 0.194507 | -0.62556 | SLC4A7 |
| 210376_x_at | 1.206529 | 0.270862 | 1.206529 | up | 1.630262 | 0.705104 | 1.630262 | up | 1.66748 | 0.737669 | 1.66748 | up | 1.509053 | 0.593643 | 1.509053 | up | 0 | 0.270862 | 0.705104 | 0.737669 | 0.593643 | ELK1 |
| 210394_x_at | 1.219013 | 0.285713 | 1.219013 | up | 1.361212 | 0.444892 | 1.361212 | up | 1.286345 | 0.363278 | 1.286345 | up | 1.586085 | 0.66547 | 1.586085 | up | 0 | 0.285713 | 0.444892 | 0.363278 | 0.66547 | SSX4///SSX4B |
| 210513_s_at | 1.140263 | 0.189367 | 1.140263 | up | 1.579504 | 0.659472 | 1.579504 | up | 1.411396 | 0.497123 | 1.411396 | up | 1.798175 | 0.846533 | 1.798175 | up | 0 | 0.189367 | 0.659472 | 0.497123 | 0.846533 | VEGFA |
| 210523_at | -1.64876 | -0.72138 | 1.648758 | down | -1.24976 | -0.32165 | 1.249756 | down | -1.23389 | -0.30321 | 1.233889 | down | -1.34554 | -0.42818 | 1.345539 | down | 0 | -0.72138 | -0.32165 | -0.30321 | -0.42818 | BMPR1B |
| 210538_s_at | -1.07674 | -0.10667 | 1.076739 | down | -1.12216 | -0.16627 | 1.122157 | down | 1.694764 | 0.761085 | 1.694764 | up | -2.08403 | -1.05937 | 2.084027 | down | 0 | -0.10667 | -0.16627 | 0.761085 | -1.05937 | BIRC3 |
| 210620_s_at | 1.262117 | 0.335846 | 1.262117 | up | 1.262117 | 0.335846 | 1.262117 | up | 1.52651 | 0.610237 | 1.52651 | up | 1.38332 | 0.468135 | 1.38332 | up | 0 | 0.335846 | 0.335846 | 0.610237 | 0.468135 | GTF3C2 |
| 210622_x_at | 1.042866 | 0.060554 | 1.042866 | up | 1.322373 | 0.403129 | 1.322373 | up | 1.284216 | 0.360888 | 1.284216 | up | 1.687495 | 0.754883 | 1.687495 | up | 0 | 0.060554 | 0.403129 | 0.360888 | 0.754883 | CDK10 |
| 210747_at | 1.515485 | 0.59978 | 1.515485 | up | 1.172904 | 0.230084 | 1.172904 | up | 1.172904 | 0.230084 | 1.172904 | up | 1.056836 | 0.079752 | 1.056836 | up | 0 | 0.59978 | 0.230084 | 0.230084 | 0.079752 | HLA-DQB1 |
| 210755_at | -1.07208 | -0.10042 | 1.072083 | down | 1.148633 | 0.199917 | 1.148633 | up | -1.28852 | -0.36571 | 1.288519 | down | -1.71823 | -0.78092 | 1.718226 | down | 0 | -0.10042 | 0.199917 | -0.36571 | -0.78092 | HGF |
| 210791_s_at | 1.655091 | 0.72691 | 1.655091 | up | 1.283731 | 0.360343 | 1.283731 | up | 1.477547 | 0.563204 | 1.477547 | up | 1.234287 | 0.303678 | 1.234287 | up | 0 | 0.72691 | 0.360343 | 0.563204 | 0.303678 | ARHGAP32 |
| 210807_s_at | 1.245978 | 0.317278 | 1.245978 | up | 1.245978 | 0.317278 | 1.245978 | up | 1.535448 | 0.61866 | 1.535448 | up | 1.135323 | 0.183103 | 1.135323 | up | 0 | 0.317278 | 0.317278 | 0.61866 | 0.183103 | SLC16A7 |
| 210815_s_at | 1.10397 | 0.142701 | 1.10397 | up | -1.28753 | -0.3646 | 1.287528 | down | -1.39464 | -0.47989 | 1.39464 | down | -1.60327 | -0.68102 | 1.603271 | down | 0 | 0.142701 | -0.3646 | -0.47989 | -0.68102 | CALCRL |
| 210973_s_at | 1.16315 | 0.218038 | 1.16315 | up | 1.372919 | 0.457246 | 1.372919 | up | 1.272239 | 0.34737 | 1.272239 | up | 1.737415 | 0.796942 | 1.737415 | up | 0 | 0.218038 | 0.457246 | 0.34737 | 0.796942 | FGFR1 |
| 210974_s_at | 1.295827 | 0.373873 | 1.295827 | up | 1.444762 | 0.530832 | 1.444762 | up | 1.559992 | 0.641538 | 1.559992 | up | 1.667209 | 0.737435 | 1.667209 | up | 0 | 0.373873 | 0.530832 | 0.641538 | 0.737435 | AP3D1 |
| 211019_s_at | 1.039048 | 0.055262 | 1.039048 | up | 1.328585 | 0.40989 | 1.328585 | up | 1.328585 | 0.40989 | 1.328585 | up | 1.948448 | 0.962325 | 1.948448 | up | 0 | 0.055262 | 0.40989 | 0.40989 | 0.962325 | LSS |
| 211022_s_at | 1.633763 | 0.708199 | 1.633763 | up | 1.090641 | 0.125176 | 1.090641 | up | 1.319046 | 0.399495 | 1.319046 | up | 1.211272 | 0.276523 | 1.211272 | up | 0 | 0.708199 | 0.125176 | 0.399495 | 0.276523 | ATRX |
| 211059_s_at | 1.146377 | 0.197082 | 1.146377 | up | 1.146377 | 0.197082 | 1.146377 | up | 1.530724 | 0.614214 | 1.530724 | up | 1.185598 | 0.245615 | 1.185598 | up | 0 | 0.197082 | 0.197082 | 0.614214 | 0.245615 | GOLGA2 |
| 211136_s_at | 1.152796 | 0.205138 | 1.152796 | up | 1.345321 | 0.42795 | 1.345321 | up | 1.30575 | 0.384878 | 1.30575 | up | 1.751862 | 0.808889 | 1.751862 | up | 0 | 0.205138 | 0.42795 | 0.384878 | 0.808889 | CLPTM1 |
| 211162_x_at | 1.049682 | 0.069952 | 1.049682 | up | 1.285927 | 0.362808 | 1.285927 | up | 1.242098 | 0.312779 | 1.242098 | up | 1.808696 | 0.85495 | 1.808696 | up | 0 | 0.069952 | 0.362808 | 0.312779 | 0.85495 | SCD |
| 211195_s_at | -1.69544 | -0.76166 | 1.695443 | down | 1.028451 | 0.040473 | 1.028451 | up | -1.23904 | -0.30922 | 1.239039 | down | -1.50413 | -0.58893 | 1.504127 | down | 0 | -0.76166 | 0.040473 | -0.30922 | -0.58893 | TP63 |
| 211205_x_at | 1.553738 | 0.635744 | 1.553738 | up | 1.314828 | 0.394874 | 1.314828 | up | 1.824428 | 0.867445 | 1.824428 | up | -1.16316 | -0.21805 | 1.163164 | down | 0 | 0.635744 | 0.394874 | 0.867445 | -0.21805 | PIP5K1A |
| 211274_at | -1.53878 | -0.62178 | 1.538778 | down | -1.17394 | -0.23136 | 1.173944 | down | -1.46406 | -0.54997 | 1.464057 | down | -1.36905 | -0.45317 | 1.369047 | down | 0 | -0.62178 | -0.23136 | -0.54997 | -0.45317 | TBX1 |
| 211317_s_at | 1.25917 | 0.332473 | 1.25917 | up | 1.25917 | 0.332473 | 1.25917 | up | 1.577835 | 0.657947 | 1.577835 | up | 1.34766 | 0.430456 | 1.34766 | up | 0 | 0.332473 | 0.332473 | 0.657947 | 0.430456 | CFLAR |
| 211391_s_at | 1.191129 | 0.25233 | 1.191129 | up | 1.488438 | 0.573799 | 1.488438 | up | 1.698083 | 0.763907 | 1.698083 | up | 2.115857 | 1.081242 | 2.115857 | up | 0 | 0.25233 | 0.573799 | 0.763907 | 1.081242 | PATZ1 |
| 211417_x_at | -1.13965 | -0.1886 | 1.139655 | down | -1.52644 | -0.61017 | 1.526444 | down | -1.19361 | -0.25533 | 1.193611 | down | -1.19361 | -0.25533 | 1.193611 | down | 0 | -0.1886 | -0.61017 | -0.25533 | -0.25533 | GGT1///GGT2///GGTLC1///GGTLC2///LOC102724197 |
| 211506_s_at | -1 | 0 | 1 | down | -1.13556 | -0.1834 | 1.135557 | down | 2.462165 | 1.299927 | 2.462165 | up | -1.45003 | -0.53608 | 1.450032 | down | 0 | 0 | -0.1834 | 1.299927 | -0.53608 | CXCL8 |
| 211527_x_at | 1.344684 | 0.427267 | 1.344684 | up | 1.908497 | 0.932437 | 1.908497 | up | 1.65032 | 0.722745 | 1.65032 | up | 1.791924 | 0.841509 | 1.791924 | up | 0 | 0.427267 | 0.932437 | 0.722745 | 0.841509 | VEGFA |
| 211541_s_at | 1.180916 | 0.239906 | 1.180916 | up | 1.004068 | 0.005857 | 1.004068 | up | 1.590844 | 0.669792 | 1.590844 | up | 1.294262 | 0.372129 | 1.294262 | up | 0 | 0.239906 | 0.005857 | 0.669792 | 0.372129 | DYRK1A |
| 211600_at | 1.529149 | 0.612729 | 1.529149 | up | 1.031506 | 0.044752 | 1.031506 | up | 1.049071 | 0.069112 | 1.049071 | up | -1.06672 | -0.09318 | 1.066716 | down | 0 | 0.612729 | 0.044752 | 0.069112 | -0.09318 | PTPRO |
| 211605_s_at | 1.275608 | 0.351185 | 1.275608 | up | 1.512949 | 0.597363 | 1.512949 | up | 1.497259 | 0.582324 | 1.497259 | up | 1.382087 | 0.466848 | 1.382087 | up | 0 | 0.351185 | 0.597363 | 0.582324 | 0.466848 | RARA |
| 211611_s_at | 1.229807 | 0.298431 | 1.229807 | up | 2.125639 | 1.087896 | 2.125639 | up | 2.719025 | 1.44309 | 2.719025 | up | 2.397364 | 1.261449 | 2.397364 | up | 0 | 0.298431 | 1.087896 | 1.44309 | 1.261449 | ATF6B///TNXB |
| 211708_s_at | 1.108885 | 0.149109 | 1.108885 | up | 1.293738 | 0.371546 | 1.293738 | up | 1.122149 | 0.166264 | 1.122149 | up | 1.682314 | 0.750447 | 1.682314 | up | 0 | 0.149109 | 0.371546 | 0.166264 | 0.750447 | SCD |
| 211829_s_at | 1.179326 | 0.237962 | 1.179326 | up | 1.97248 | 0.980011 | 1.97248 | up | 1.678648 | 0.7473 | 1.678648 | up | 1.24523 | 0.316413 | 1.24523 | up | 0 | 0.237962 | 0.980011 | 0.7473 | 0.316413 | GPER1 |
| 211833_s_at | 1.578399 | 0.658462 | 1.578399 | up | 1.285941 | 0.362825 | 1.285941 | up | 1.238248 | 0.308301 | 1.238248 | up | 1.175563 | 0.233351 | 1.175563 | up | 0 | 0.658462 | 0.362825 | 0.308301 | 0.233351 | BAX |
| 211888_x_at | -1.50095 | -0.58588 | 1.50095 | down | -1 | 0 | 1 | down | -1 | 0 | 1 | down | 1.154954 | 0.207835 | 1.154954 | up | 0 | -0.58588 | 0 | 0 | 0.207835 | CASP10 |
| 211950_at | 1.180005 | 0.238792 | 1.180005 | up | 1.132792 | 0.179883 | 1.132792 | up | 1.196378 | 0.258673 | 1.196378 | up | 1.561561 | 0.642989 | 1.561561 | up | 0 | 0.238792 | 0.179883 | 0.258673 | 0.642989 | UBR4 |
| 211993_at | 1.132534 | 0.179554 | 1.132534 | up | 1.075024 | 0.104369 | 1.075024 | up | 1.717114 | 0.779985 | 1.717114 | up | 1.18666 | 0.246907 | 1.18666 | up | 0 | 0.179554 | 0.104369 | 0.779985 | 0.246907 | WNK1 |
| 212005_at | 1.044946 | 0.063428 | 1.044946 | up | 1.183224 | 0.242723 | 1.183224 | up | 1.183224 | 0.242723 | 1.183224 | up | 1.533535 | 0.616861 | 1.533535 | up | 0 | 0.063428 | 0.242723 | 0.242723 | 0.616861 | SZRD1 |
| 212016_s_at | 1.211031 | 0.276236 | 1.211031 | up | 1.904868 | 0.929691 | 1.904868 | up | 1.812812 | 0.85823 | 1.812812 | up | 2.375615 | 1.248301 | 2.375615 | up | 0 | 0.276236 | 0.929691 | 0.85823 | 1.248301 | PTBP1 |
| 212019_at | 1.063717 | 0.089114 | 1.063717 | up | 1.277706 | 0.353556 | 1.277706 | up | 1.298758 | 0.377133 | 1.298758 | up | 1.688125 | 0.755421 | 1.688125 | up | 0 | 0.089114 | 0.353556 | 0.377133 | 0.755421 | RSL1D1 |
| 212070_at | -1.01486 | -0.02128 | 1.014858 | down | 1.176442 | 0.23443 | 1.176442 | up | 1.104099 | 0.142869 | 1.104099 | up | 1.547463 | 0.629905 | 1.547463 | up | 0 | -0.02128 | 0.23443 | 0.142869 | 0.629905 | ADGRG1 |
| 212105_s_at | 1.173285 | 0.230553 | 1.173285 | up | 1.710378 | 0.774315 | 1.710378 | up | 1.339615 | 0.421818 | 1.339615 | up | 1.413805 | 0.499583 | 1.413805 | up | 0 | 0.230553 | 0.774315 | 0.421818 | 0.499583 | DHX9 |
| 212125_at | 1.053954 | 0.075811 | 1.053954 | up | 1.506702 | 0.591394 | 1.506702 | up | 1.364252 | 0.44811 | 1.364252 | up | 1.630638 | 0.705437 | 1.630638 | up | 0 | 0.075811 | 0.591394 | 0.44811 | 0.705437 | RANGAP1 |
| 212218_s_at | 1.066189 | 0.092463 | 1.066189 | up | -1.00077 | -0.0011 | 1.000766 | down | 1.036737 | 0.05205 | 1.036737 | up | 1.706576 | 0.771105 | 1.706576 | up | 0 | 0.092463 | -0.0011 | 0.05205 | 0.771105 | FASN |
| 212225_at | 1.201442 | 0.264767 | 1.201442 | up | 1.165335 | 0.220745 | 1.165335 | up | 1.649997 | 0.722463 | 1.649997 | up | 1.167548 | 0.223482 | 1.167548 | up | 0 | 0.264767 | 0.220745 | 0.722463 | 0.223482 | EIF1 |
| 212393_at | 1.100631 | 0.138331 | 1.100631 | up | 1.254469 | 0.327077 | 1.254469 | up | 1.522924 | 0.606844 | 1.522924 | up | 1.662184 | 0.73308 | 1.662184 | up | 0 | 0.138331 | 0.327077 | 0.606844 | 0.73308 | SBF1 |
| 212489_at | 1.166123 | 0.22172 | 1.166123 | up | 1.288336 | 0.365509 | 1.288336 | up | 1.150256 | 0.201955 | 1.150256 | up | 1.516452 | 0.600699 | 1.516452 | up | 0 | 0.22172 | 0.365509 | 0.201955 | 0.600699 | COL5A1 |
| 212492_s_at | 1.115989 | 0.158323 | 1.115989 | up | 1.151235 | 0.203183 | 1.151235 | up | 1.191842 | 0.253193 | 1.191842 | up | 1.553037 | 0.635092 | 1.553037 | up | 0 | 0.158323 | 0.203183 | 0.253193 | 0.635092 | KDM4B |
| 212665_at | 1.139222 | 0.188048 | 1.139222 | up | 1.285946 | 0.36283 | 1.285946 | up | 1.796009 | 0.844794 | 1.796009 | up | 1.53945 | 0.622415 | 1.53945 | up | 0 | 0.188048 | 0.36283 | 0.844794 | 0.622415 | TIPARP |
| 212843_at | -1.50095 | -0.58588 | 1.500949 | down | -1.1754 | -0.23315 | 1.175401 | down | -1 | 0 | 1 | down | 1.147364 | 0.198323 | 1.147364 | up | 0 | -0.58588 | -0.23315 | 0 | 0.198323 | NCAM1 |
| 212921_at | 1.555807 | 0.637663 | 1.555807 | up | -1 | 0 | 1 | down | -1 | 0 | 1 | down | -1.01805 | -0.02581 | 1.018052 | down | 0 | 0.637663 | 0 | 0 | -0.02581 | SMYD2 |
| 212937_s_at | -1.01259 | -0.01805 | 1.012589 | down | -1 | 0 | 1 | down | -1.02393 | -0.03412 | 1.02393 | down | 1.614296 | 0.690906 | 1.614296 | up | 0 | -0.01805 | 0 | -0.03412 | 0.690906 | COL6A1 |
| 213036_x_at | 1.085821 | 0.118786 | 1.085821 | up | 1.316112 | 0.396283 | 1.316112 | up | 1.316112 | 0.396283 | 1.316112 | up | 1.574229 | 0.654645 | 1.574229 | up | 0 | 0.118786 | 0.396283 | 0.396283 | 0.654645 | ATP2A3 |
| 213112_s_at | 1.059655 | 0.083595 | 1.059655 | up | 1.214162 | 0.279961 | 1.214162 | up | 1.40849 | 0.49415 | 1.40849 | up | 1.589093 | 0.668204 | 1.589093 | up | 0 | 0.083595 | 0.279961 | 0.49415 | 0.668204 | SQSTM1 |
| 213490_s_at | 1.21159 | 0.276901 | 1.21159 | up | 1.659467 | 0.73072 | 1.659467 | up | 1.651842 | 0.724076 | 1.651842 | up | 1.92185 | 0.942495 | 1.92185 | up | 0 | 0.276901 | 0.73072 | 0.724076 | 0.942495 | MAP2K2 |
| 213522_s_at | -1.05665 | -0.0795 | 1.056654 | down | -1.56618 | -0.64725 | 1.566185 | down | -1.2572 | -0.33021 | 1.257197 | down | -1.17561 | -0.23341 | 1.175611 | down | 0 | -0.0795 | -0.64725 | -0.33021 | -0.23341 | CSNK1D |
| 213570_at | -1.23432 | -0.30371 | 1.234319 | down | -1.0219 | -0.03126 | 1.021904 | down | -1.06659 | -0.09301 | 1.06659 | down | -1.55646 | -0.63827 | 1.556459 | down | 0 | -0.30371 | -0.03126 | -0.09301 | -0.63827 | EIF4E2 |
| 213618_at | 1.270109 | 0.344952 | 1.270109 | up | 1.570352 | 0.651088 | 1.570352 | up | 1.226018 | 0.29398 | 1.226018 | up | -1.0605 | -0.08474 | 1.060497 | down | 0 | 0.344952 | 0.651088 | 0.29398 | -0.08474 | ARAP2 |
| 213640_s_at | 1.219477 | 0.286263 | 1.219477 | up | 1.596281 | 0.674715 | 1.596281 | up | 1.318381 | 0.398767 | 1.318381 | up | 1.334567 | 0.416371 | 1.334567 | up | 0 | 0.286263 | 0.674715 | 0.398767 | 0.416371 | LOX |
| 213746_s_at | 1.230667 | 0.29944 | 1.230667 | up | 1.23462 | 0.304067 | 1.23462 | up | 1.339667 | 0.421874 | 1.339667 | up | 1.719672 | 0.782133 | 1.719672 | up | 0 | 0.29944 | 0.304067 | 0.421874 | 0.782133 | FLNA |
| 213813_x_at | 1.272566 | 0.347741 | 1.272566 | up | 1.290337 | 0.367748 | 1.290337 | up | 1.649188 | 0.721756 | 1.649188 | up | -1.09482 | -0.1307 | 1.094825 | down | 0 | 0.347741 | 0.367748 | 0.721756 | -0.1307 |  |
| 213827_at | -1.10238 | -0.14062 | 1.102377 | down | -1.36377 | -0.4476 | 1.363767 | down | -1.52468 | -0.60851 | 1.52468 | down | -1.10318 | -0.14167 | 1.103183 | down | 0 | -0.14062 | -0.4476 | -0.60851 | -0.14167 | ARHGAP33 |
| 213882_at | -1.14939 | -0.20087 | 1.149389 | down | 1.085674 | 0.118591 | 1.085674 | up | 1.136909 | 0.185117 | 1.136909 | up | -1.54769 | -0.63011 | 1.547688 | down | 0 | -0.20087 | 0.118591 | 0.185117 | -0.63011 | TM2D1 |
| 213887_s_at | 1.03399 | 0.048222 | 1.03399 | up | 1.407633 | 0.493271 | 1.407633 | up | 1.334314 | 0.416098 | 1.334314 | up | 1.601172 | 0.679129 | 1.601172 | up | 0 | 0.048222 | 0.493271 | 0.416098 | 0.679129 | POLR2E |
| 213922_at | 1.231128 | 0.29998 | 1.231128 | up | 1.272737 | 0.347934 | 1.272737 | up | 1.673969 | 0.743273 | 1.673969 | up | 1.084142 | 0.116553 | 1.084142 | up | 0 | 0.29998 | 0.347934 | 0.743273 | 0.116553 | TTBK2 |
| 213984_at | -1.38974 | -0.47482 | 1.389741 | down | 1.075079 | 0.104443 | 1.075079 | up | -1.07959 | -0.11049 | 1.079593 | down | -1.52541 | -0.6092 | 1.525408 | down | 0 | -0.47482 | 0.104443 | -0.11049 | -0.6092 | PDS5A |
| 213987_s_at | -1.12977 | -0.17603 | 1.129774 | down | -1.58959 | -0.66865 | 1.589589 | down | -1.48186 | -0.56741 | 1.481865 | down | -1.0469 | -0.06613 | 1.046902 | down | 0 | -0.17603 | -0.66865 | -0.56741 | -0.06613 | CDK13 |
| 214040_s_at | -1.20261 | -0.26617 | 1.202611 | down | 1.615388 | 0.691881 | 1.615388 | up | 1.615388 | 0.691881 | 1.615388 | up | 2.054395 | 1.038714 | 2.054395 | up | 0 | -0.26617 | 0.691881 | 0.691881 | 1.038714 | GSN |
| 214169_at | -1.16454 | -0.21976 | 1.164537 | down | -1.16454 | -0.21976 | 1.164537 | down | -1.07262 | -0.10113 | 1.072616 | down | -1.50902 | -0.59361 | 1.509016 | down | 0 | -0.21976 | -0.21976 | -0.10113 | -0.59361 | SUN1 |
| 214251_s_at | 1.05056 | 0.071159 | 1.05056 | up | 1.274291 | 0.349694 | 1.274291 | up | 1.308108 | 0.387481 | 1.308108 | up | 1.801122 | 0.848896 | 1.801122 | up | 0 | 0.071159 | 0.349694 | 0.387481 | 0.848896 | NUMA1 |
| 214310_s_at | 1.125181 | 0.170156 | 1.125181 | up | 1.460078 | 0.546045 | 1.460078 | up | 1.153775 | 0.206362 | 1.153775 | up | 1.529566 | 0.613122 | 1.529566 | up | 0 | 0.170156 | 0.546045 | 0.206362 | 0.613122 | ZFPL1 |
| 214417_s_at | -1.5381 | -0.62115 | 1.538096 | down | -1.22805 | -0.29637 | 1.228051 | down | -1.19828 | -0.26096 | 1.198278 | down | -1.24514 | -0.31631 | 1.245145 | down | 0 | -0.62115 | -0.29637 | -0.26096 | -0.31631 | LOC105374258 |
| 214478_at | 1.073569 | 0.102414 | 1.073569 | up | 1.615176 | 0.691691 | 1.615176 | up | 1.393759 | 0.478981 | 1.393759 | up | 1.20918 | 0.274029 | 1.20918 | up | 0 | 0.102414 | 0.691691 | 0.478981 | 0.274029 | SPP2 |
| 214576_at | 1.898448 | 0.92482 | 1.898448 | up | 1.150344 | 0.202065 | 1.150344 | up | 1.114749 | 0.156718 | 1.114749 | up | 1.150344 | 0.202065 | 1.150344 | up | 0 | 0.92482 | 0.202065 | 0.156718 | 0.202065 | KRT36 |
| 214657_s_at | 1.947362 | 0.961521 | 1.947362 | up | 1.273761 | 0.349094 | 1.273761 | up | 1.54627 | 0.628793 | 1.54627 | up | 1.130732 | 0.177257 | 1.130732 | up | 0 | 0.961521 | 0.349094 | 0.628793 | 0.177257 | NEAT1 |
| 214671_s_at | 1.019555 | 0.02794 | 1.019555 | up | 1.714082 | 0.777436 | 1.714082 | up | 1.400475 | 0.485917 | 1.400475 | up | 1.968499 | 0.977096 | 1.968499 | up | 0 | 0.02794 | 0.777436 | 0.485917 | 0.977096 | ABR |
| 214722_at | 1.298747 | 0.37712 | 1.298747 | up | -1.00649 | -0.00933 | 1.006489 | down | 1.221514 | 0.288671 | 1.221514 | up | 1.542085 | 0.624883 | 1.542085 | up | 0 | 0.37712 | -0.00933 | 0.288671 | 0.624883 | NOTCH2NL |
| 214727_at | 1.29067 | 0.36812 | 1.29067 | up | 1.446805 | 0.532871 | 1.446805 | up | 1.370825 | 0.455045 | 1.370825 | up | 1.527387 | 0.611065 | 1.527387 | up | 0 | 0.36812 | 0.532871 | 0.455045 | 0.611065 | BRCA2 |
| 214752_x_at | 1.174977 | 0.232633 | 1.174977 | up | 1.294656 | 0.372569 | 1.294656 | up | 1.326091 | 0.40718 | 1.326091 | up | 1.523964 | 0.607829 | 1.523964 | up | 0 | 0.232633 | 0.372569 | 0.40718 | 0.607829 | FLNA |
| 214860_at | 1.719762 | 0.782209 | 1.719762 | up | 1.449658 | 0.535713 | 1.449658 | up | 1.757381 | 0.813427 | 1.757381 | up | 1.362187 | 0.445925 | 1.362187 | up | 0 | 0.782209 | 0.535713 | 0.813427 | 0.445925 | SLC9A7 |
| 214891_at | 1.538069 | 0.62112 | 1.538069 | up | -1.011 | -0.01578 | 1.010997 | down | 1.230566 | 0.299322 | 1.230566 | up | 1.058369 | 0.081843 | 1.058369 | up | 0 | 0.62112 | -0.01578 | 0.299322 | 0.081843 | FBXO21 |
| 215099_s_at | 1.14938 | 0.200856 | 1.14938 | up | 1.684535 | 0.75235 | 1.684535 | up | 1.829723 | 0.871625 | 1.829723 | up | 2.202816 | 1.139349 | 2.202816 | up | 0 | 0.200856 | 0.75235 | 0.871625 | 1.139349 | RXRB |
| 215185_at | 1.226923 | 0.295045 | 1.226923 | up | 1.378798 | 0.463411 | 1.378798 | up | 1.378798 | 0.463411 | 1.378798 | up | 1.556249 | 0.638073 | 1.556249 | up | 0 | 0.295045 | 0.463411 | 0.463411 | 0.638073 | LINC00963 |
| 215210_s_at | 1.530148 | 0.613671 | 1.530148 | up | 1.263202 | 0.337085 | 1.263202 | up | 1.508248 | 0.592874 | 1.508248 | up | 1.375079 | 0.459515 | 1.375079 | up | 0 | 0.613671 | 0.337085 | 0.592874 | 0.459515 | DLST |
| 215236_s_at | 2.735069 | 1.451577 | 2.735069 | up | 1.218484 | 0.285088 | 1.218484 | up | 1.319246 | 0.399714 | 1.319246 | up | 1.07232 | 0.100735 | 1.07232 | up | 0 | 1.451577 | 0.285088 | 0.399714 | 0.100735 | PICALM |
| 215282_at | -1.50931 | -0.59389 | 1.509314 | down | -1.03411 | -0.04839 | 1.034108 | down | -1.00834 | -0.01199 | 1.008342 | down | -1.17182 | -0.22875 | 1.171821 | down | 0 | -0.59389 | -0.04839 | -0.01199 | -0.22875 | ANAPC13 |
| 215325_x_at | 1.589118 | 0.668226 | 1.589118 | up | 1.423468 | 0.50941 | 1.423468 | up | 1.293025 | 0.37075 | 1.293025 | up | 2.207048 | 1.142118 | 2.207048 | up | 0 | 0.668226 | 0.50941 | 0.37075 | 1.142118 | CBARP |
| 215357_s_at | 1.160215 | 0.214392 | 1.160215 | up | 1.501475 | 0.586381 | 1.501475 | up | 1.283998 | 0.360642 | 1.283998 | up | 1.283998 | 0.360642 | 1.283998 | up | 0 | 0.214392 | 0.586381 | 0.360642 | 0.360642 | POLDIP3 |
| 215384_s_at | 1.377869 | 0.462439 | 1.377869 | up | 1.167971 | 0.224004 | 1.167971 | up | -1.0109 | -0.01564 | 1.0109 | down | 1.503771 | 0.588585 | 1.503771 | up | 0 | 0.462439 | 0.224004 | -0.01564 | 0.588585 | MAP1A |
| 215391_at | -1.04223 | -0.05968 | 1.042235 | down | 1.046094 | 0.065012 | 1.046094 | up | 1.566854 | 0.647871 | 1.566854 | up | 1.046094 | 0.065012 | 1.046094 | up | 0 | -0.05968 | 0.065012 | 0.647871 | 0.065012 | MAP1A |
| 215489_x_at | 1.118355 | 0.161378 | 1.118355 | up | 1.609445 | 0.686563 | 1.609445 | up | 1.341223 | 0.423549 | 1.341223 | up | 1.697679 | 0.763564 | 1.697679 | up | 0 | 0.161378 | 0.686563 | 0.423549 | 0.763564 | HOMER3 |
| 215581_s_at | 1.093794 | 0.129341 | 1.093794 | up | 1.655278 | 0.727073 | 1.655278 | up | 1.36316 | 0.446955 | 1.36316 | up | 1.312948 | 0.39281 | 1.312948 | up | 0 | 0.129341 | 0.727073 | 0.446955 | 0.39281 | MCM3AP |
| 215649_s_at | -1.09664 | -0.13309 | 1.096638 | down | 1.342662 | 0.425096 | 1.342662 | up | 1.225376 | 0.293224 | 1.225376 | up | 1.620165 | 0.696141 | 1.620165 | up | 0 | -0.13309 | 0.425096 | 0.293224 | 0.696141 | MVK |
| 215708_s_at | 1.063146 | 0.088339 | 1.063146 | up | 1.502187 | 0.587064 | 1.502187 | up | 1.362821 | 0.446596 | 1.362821 | up | 1.495961 | 0.581072 | 1.495961 | up | 0 | 0.088339 | 0.587064 | 0.446596 | 0.581072 | PRIM2 |
| 215781_s_at | 1.190631 | 0.251727 | 1.190631 | up | 1.27411 | 0.34949 | 1.27411 | up | 1.233722 | 0.303018 | 1.233722 | up | 1.553884 | 0.635879 | 1.553884 | up | 0 | 0.251727 | 0.34949 | 0.303018 | 0.635879 | TOP3B |
| 215810_x_at | 1.256074 | 0.328922 | 1.256074 | up | 1.222258 | 0.289548 | 1.222258 | up | 1.51761 | 0.601801 | 1.51761 | up | 1.222258 | 0.289548 | 1.222258 | up | 0 | 0.328922 | 0.289548 | 0.601801 | 0.289548 | DST |
| 215832_x_at | 1.861577 | 0.896525 | 1.861577 | up | 1.111383 | 0.152356 | 1.111383 | up | 1.185285 | 0.245234 | 1.185285 | up | 1.108828 | 0.149035 | 1.108828 | up | 0 | 0.896525 | 0.152356 | 0.245234 | 0.149035 | PICALM |
| 215984_s_at | 1.067843 | 0.0947 | 1.067843 | up | 1.586828 | 0.666146 | 1.586828 | up | 1.310561 | 0.390184 | 1.310561 | up | 1.600517 | 0.678538 | 1.600517 | up | 0 | 0.0947 | 0.666146 | 0.390184 | 0.678538 | ARFRP1 |
| 216009_at | -1.50831 | -0.59293 | 1.508309 | down | -1.17214 | -0.22915 | 1.172141 | down | -1.30303 | -0.38187 | 1.303028 | down | -1.55517 | -0.63707 | 1.555172 | down | 0 | -0.59293 | -0.22915 | -0.38187 | -0.63707 | SLC39A9 |
| 216036_x_at | -1.00076 | -0.0011 | 1.000763 | down | -1.50098 | -0.58591 | 1.500981 | down | -1.02858 | -0.04066 | 1.028584 | down | 1.142823 | 0.192602 | 1.142823 | up | 0 | -0.0011 | -0.58591 | -0.04066 | 0.192602 | WDTC1 |
| 216205_s_at | -1.06543 | -0.09144 | 1.065435 | down | 1.467282 | 0.553146 | 1.467282 | up | 1.392831 | 0.478021 | 1.392831 | up | 1.575275 | 0.655604 | 1.575275 | up | 0 | -0.09144 | 0.553146 | 0.478021 | 0.655604 | MFN2 |
| 216222_s_at | 1.095325 | 0.131359 | 1.095325 | up | 1.142318 | 0.191965 | 1.142318 | up | 1.673064 | 0.742493 | 1.673064 | up | 1.142318 | 0.191965 | 1.142318 | up | 0 | 0.131359 | 0.191965 | 0.742493 | 0.191965 | MYO10 |
| 216230_x_at | 1.14106 | 0.190375 | 1.14106 | up | 1.589276 | 0.668369 | 1.589276 | up | 1.127559 | 0.173203 | 1.127559 | up | 1.422799 | 0.508731 | 1.422799 | up | 0 | 0.190375 | 0.668369 | 0.173203 | 0.508731 | SMPD1 |
| 216281_at | -1.56871 | -0.64957 | 1.568705 | down | -1.52731 | -0.61099 | 1.527312 | down | -1.5032 | -0.58804 | 1.503199 | down | -1.52731 | -0.61099 | 1.527312 | down | 0 | -0.64957 | -0.61099 | -0.58804 | -0.61099 | DICER1 |
| 216319_at | -1.56529 | -0.64643 | 1.565287 | down | -1.21545 | -0.2815 | 1.215454 | down | -1.16102 | -0.21539 | 1.161021 | down | -1.21917 | -0.2859 | 1.21917 | down | 0 | -0.64643 | -0.2815 | -0.21539 | -0.2859 |  |
| 216375_s_at | 1.064567 | 0.090267 | 1.064567 | up | -1.0918 | -0.12671 | 1.0918 | down | -1.51468 | -0.59901 | 1.514679 | down | -1.1729 | -0.23008 | 1.1729 | down | 0 | 0.090267 | -0.12671 | -0.59901 | -0.23008 | ETV5 |
| 216436_at | -1.24327 | -0.31414 | 1.243269 | down | -1.57653 | -0.65675 | 1.576529 | down | -1.24327 | -0.31414 | 1.243269 | down | -1.27141 | -0.34643 | 1.271413 | down | 0 | -0.31414 | -0.65675 | -0.31414 | -0.34643 | PIK3R4 |
| 216480_x_at | 1.538493 | 0.621518 | 1.538493 | up | -1.06592 | -0.09209 | 1.065916 | down | -1.19077 | -0.2519 | 1.190773 | down | -1 | 0 | 1 | down | 0 | 0.621518 | -0.09209 | -0.2519 | 0 | MLLT10 |
| 216482_x_at | -1.66645 | -0.73678 | 1.666448 | down | 1.12989 | 0.176182 | 1.12989 | up | -1.28332 | -0.35988 | 1.283317 | down | -1.14008 | -0.18914 | 1.140081 | down | 0 | -0.73678 | 0.176182 | -0.35988 | -0.18914 | ZNF79 |
| 216501_at | -1.74303 | -0.8016 | 1.743027 | down | -1.29404 | -0.37188 | 1.294037 | down | -1.35702 | -0.44045 | 1.357024 | down | -1.45483 | -0.54085 | 1.454826 | down | 0 | -0.8016 | -0.37188 | -0.44045 | -0.54085 | VAC14 |
| 216627_s_at | 1.000815 | 0.001176 | 1.000815 | up | 1.78758 | 0.838007 | 1.78758 | up | 1.212963 | 0.278535 | 1.212963 | up | 1.408458 | 0.494117 | 1.408458 | up | 0 | 0.001176 | 0.838007 | 0.278535 | 0.494117 | B4GALT1 |
| 216655_s_at | 1.314761 | 0.3948 | 1.314761 | up | 1.475611 | 0.561312 | 1.475611 | up | 1.314761 | 0.3948 | 1.314761 | up | 1.611694 | 0.688578 | 1.611694 | up | 0 | 0.3948 | 0.561312 | 0.3948 | 0.688578 |  |
| 216717_at | -1.60216 | -0.68002 | 1.602165 | down | -1.38252 | -0.4673 | 1.38252 | down | -1.38252 | -0.4673 | 1.38252 | down | -1.33741 | -0.41944 | 1.337412 | down | 0 | -0.68002 | -0.4673 | -0.4673 | -0.41944 | SUPT20H |
| 216797_at | -1.01262 | -0.01809 | 1.012619 | down | -1.07228 | -0.10069 | 1.072284 | down | -1.0387 | -0.05477 | 1.038696 | down | 1.581218 | 0.661037 | 1.581218 | up | 0 | -0.01809 | -0.10069 | -0.05477 | 0.661037 |  |
| 216830_at | -1.22463 | -0.29235 | 1.224635 | down | -1.07868 | -0.10927 | 1.078681 | down | -1.22463 | -0.29235 | 1.224635 | down | -1.5332 | -0.61655 | 1.533201 | down | 0 | -0.29235 | -0.10927 | -0.29235 | -0.61655 | HERC2///HERC2P10 |
| 216861_at | 1.00021 | 3.02E-04 | 1.00021 | up | -1.10695 | -0.14659 | 1.10695 | down | 1.530604 | 0.614101 | 1.530604 | up | 1.00021 | 3.02E-04 | 1.00021 | up | 0 | 3.02E-04 | -0.14659 | 0.614101 | 3.02E-04 |  |
| 216969_s_at | 1.09915 | 0.136388 | 1.09915 | up | 1.622316 | 0.698055 | 1.622316 | up | 1.618171 | 0.694364 | 1.618171 | up | 1.975443 | 0.982176 | 1.975443 | up | 0 | 0.136388 | 0.698055 | 0.694364 | 0.982176 | KIF22 |
| 217173_s_at | 1.094384 | 0.130119 | 1.094384 | up | 1.435263 | 0.521316 | 1.435263 | up | 1.448054 | 0.534116 | 1.448054 | up | 1.843061 | 0.882104 | 1.843061 | up | 0 | 0.130119 | 0.521316 | 0.534116 | 0.882104 | LDLR |
| 217202_s_at | 1.197846 | 0.260442 | 1.197846 | up | 1.265732 | 0.339972 | 1.265732 | up | 1.256071 | 0.328918 | 1.256071 | up | 1.56283 | 0.644161 | 1.56283 | up | 0 | 0.260442 | 0.339972 | 0.328918 | 0.644161 | GLUL |
| 217279_x_at | 1.209727 | 0.274682 | 1.209727 | up | 1.4099 | 0.495593 | 1.4099 | up | 1.365109 | 0.449016 | 1.365109 | up | 1.870766 | 0.903629 | 1.870766 | up | 0 | 0.274682 | 0.495593 | 0.449016 | 0.903629 | MMP14 |
| 217289_s_at | 1.251924 | 0.324147 | 1.251924 | up | 1.535062 | 0.618297 | 1.535062 | up | 1.330404 | 0.411864 | 1.330404 | up | 1.767656 | 0.821838 | 1.767656 | up | 0 | 0.324147 | 0.618297 | 0.411864 | 0.821838 | SLC37A4 |
| 217304_at | 1.116068 | 0.158424 | 1.116068 | up | 1.593787 | 0.672459 | 1.593787 | up | 1.212681 | 0.2782 | 1.212681 | up | 1.164704 | 0.219963 | 1.164704 | up | 0 | 0.158424 | 0.672459 | 0.2782 | 0.219963 | SHMT1 |
| 217458_at | -1.07046 | -0.09823 | 1.070459 | down | -1.5899 | -0.66893 | 1.589898 | down | -1.12767 | -0.17335 | 1.127672 | down | -1.12767 | -0.17335 | 1.127672 | down | 0 | -0.09823 | -0.66893 | -0.17335 | -0.17335 |  |
| 217564_s_at | -1.01621 | -0.0232 | 1.016209 | down | -1.18738 | -0.24778 | 1.187378 | down | -1.03777 | -0.05348 | 1.037768 | down | -1.61663 | -0.69299 | 1.61663 | down | 0 | -0.0232 | -0.24778 | -0.05348 | -0.69299 | CPS1 |
| 217615_at | 1.575451 | 0.655765 | 1.575451 | up | -1.01248 | -0.01789 | 1.012479 | down | 1.204322 | 0.268221 | 1.204322 | up | 1.275242 | 0.350771 | 1.275242 | up | 0 | 0.655765 | -0.01789 | 0.268221 | 0.350771 |  |
| 217648_at | -1.33207 | -0.41367 | 1.332066 | down | -1.33207 | -0.41367 | 1.332066 | down | -1.56301 | -0.64433 | 1.563011 | down | -1.33207 | -0.41367 | 1.332066 | down | 0 | -0.41367 | -0.41367 | -0.64433 | -0.41367 |  |
| 217847_s_at | 1.357621 | 0.441081 | 1.357621 | up | 1.513409 | 0.597802 | 1.513409 | up | 1.697858 | 0.763716 | 1.697858 | up | 1.670418 | 0.740209 | 1.670418 | up | 0 | 0.441081 | 0.597802 | 0.763716 | 0.740209 | THRAP3 |
| 218228_s_at | 1.065991 | 0.092196 | 1.065991 | up | 1.005597 | 0.008052 | 1.005597 | up | -1.15646 | -0.20972 | 1.156461 | down | -1.93298 | -0.95082 | 1.932977 | down | 0 | 0.092196 | 0.008052 | -0.20972 | -0.95082 | TNKS2 |
| 218302_at | 1.374364 | 0.458764 | 1.374364 | up | 1.406208 | 0.49181 | 1.406208 | up | 1.455405 | 0.54142 | 1.455405 | up | 1.657222 | 0.728767 | 1.657222 | up | 0 | 0.458764 | 0.49181 | 0.54142 | 0.728767 | PSENEN |
| 218353_at | -1.30029 | -0.37884 | 1.300294 | down | -1.56482 | -0.646 | 1.564819 | down | -1.30029 | -0.37884 | 1.300294 | down | -1.30648 | -0.38569 | 1.306483 | down | 0 | -0.37884 | -0.646 | -0.37884 | -0.38569 | RGS5 |
| 218368_s_at | 1.076787 | 0.106732 | 1.076787 | up | 1.400968 | 0.486424 | 1.400968 | up | 1.443196 | 0.529267 | 1.443196 | up | 1.54474 | 0.627364 | 1.54474 | up | 0 | 0.106732 | 0.486424 | 0.529267 | 0.627364 | TNFRSF12A |
| 218509_at | 1.206574 | 0.270916 | 1.206574 | up | 1.510712 | 0.595229 | 1.510712 | up | 1.382751 | 0.467542 | 1.382751 | up | 1.362743 | 0.446513 | 1.362743 | up | 0 | 0.270916 | 0.595229 | 0.467542 | 0.446513 | PLPPR2 |
| 218621_at | -1.0579 | -0.0812 | 1.057897 | down | -1.26969 | -0.34448 | 1.269694 | down | -1.635 | -0.70929 | 1.635001 | down | 1.000674 | 9.72E-04 | 1.000674 | up | 0 | -0.0812 | -0.34448 | -0.70929 | 9.72E-04 | HEMK1 |
| 218800_at | 1.490948 | 0.57623 | 1.490948 | up | 1.634262 | 0.708639 | 1.634262 | up | 1.338631 | 0.420758 | 1.338631 | up | 1.42295 | 0.508885 | 1.42295 | up | 0 | 0.57623 | 0.708639 | 0.420758 | 0.508885 | SRD5A3 |
| 218813_s_at | 1.441718 | 0.527789 | 1.441718 | up | 1.458487 | 0.544473 | 1.458487 | up | 1.528972 | 0.612562 | 1.528972 | up | 2.075863 | 1.053711 | 2.075863 | up | 0 | 0.527789 | 0.544473 | 0.612562 | 1.053711 | SH3GLB2 |
| 219028_at | 1.174267 | 0.23176 | 1.174267 | up | 1.164064 | 0.219171 | 1.164064 | up | 1.525777 | 0.609544 | 1.525777 | up | 1.394841 | 0.4801 | 1.394841 | up | 0 | 0.23176 | 0.219171 | 0.609544 | 0.4801 | HIPK2 |
| 219171_s_at | 1.512411 | 0.59685 | 1.512411 | up | 1.473688 | 0.559431 | 1.473688 | up | 1.590491 | 0.669472 | 1.590491 | up | 1.275941 | 0.351561 | 1.275941 | up | 0 | 0.59685 | 0.559431 | 0.669472 | 0.351561 | ZNF236 |
| 219180_s_at | 1.443904 | 0.529975 | 1.443904 | up | 1.200168 | 0.263236 | 1.200168 | up | 1.542504 | 0.625275 | 1.542504 | up | 1.219327 | 0.286086 | 1.219327 | up | 0 | 0.529975 | 0.263236 | 0.625275 | 0.286086 | PEX26 |
| 219250_s_at | -1.23577 | -0.30541 | 1.235768 | down | -1.43266 | -0.5187 | 1.432665 | down | -1.60687 | -0.68425 | 1.606872 | down | -1.32294 | -0.40374 | 1.322935 | down | 0 | -0.30541 | -0.5187 | -0.68425 | -0.40374 | FLRT3 |
| 219273_at | 1.741013 | 0.799927 | 1.741013 | up | 1.129806 | 0.176075 | 1.129806 | up | 1.094054 | 0.129684 | 1.094054 | up | 1.129806 | 0.176075 | 1.129806 | up | 0 | 0.799927 | 0.176075 | 0.129684 | 0.176075 | CCNK |
| 219280_at | 1.259896 | 0.333304 | 1.259896 | up | 1.181495 | 0.240614 | 1.181495 | up | 1.520004 | 0.604075 | 1.520004 | up | 1.251138 | 0.323241 | 1.251138 | up | 0 | 0.333304 | 0.240614 | 0.604075 | 0.323241 | BRWD1 |
| 219528_s_at | -1.39023 | -0.47532 | 1.390231 | down | -1.3568 | -0.44021 | 1.356801 | down | -1.5075 | -0.59216 | 1.507503 | down | -1.3568 | -0.44021 | 1.356801 | down | 0 | -0.47532 | -0.44021 | -0.59216 | -0.44021 | BCL11B |
| 219535_at | 1.507173 | 0.591845 | 1.507173 | up | 1.225677 | 0.293579 | 1.225677 | up | -1.0787 | -0.10929 | 1.078697 | down | 1.09608 | 0.132353 | 1.09608 | up | 0 | 0.591845 | 0.293579 | -0.10929 | 0.132353 | HUNK |
| 219728_at | 1.661947 | 0.732874 | 1.661947 | up | 1.06483 | 0.090623 | 1.06483 | up | -1.10241 | -0.14066 | 1.102406 | down | 1.255781 | 0.328584 | 1.255781 | up | 0 | 0.732874 | 0.090623 | -0.14066 | 0.328584 | MYOT |
| 219820_at | -1.08936 | -0.12348 | 1.089358 | down | -1.51604 | -0.60031 | 1.516038 | down | -1.0158 | -0.02262 | 1.015799 | down | -1.08309 | -0.11516 | 1.083093 | down | 0 | -0.12348 | -0.60031 | -0.02262 | -0.11516 | SLC6A16 |
| 219922_s_at | 1.087217 | 0.12064 | 1.087217 | up | 1.527252 | 0.610938 | 1.527252 | up | 1.432505 | 0.51854 | 1.432505 | up | 1.710205 | 0.774169 | 1.710205 | up | 0 | 0.12064 | 0.610938 | 0.51854 | 0.774169 | LTBP3 |
| 219949_at | 1.03525 | 0.049979 | 1.03525 | up | 1.11702 | 0.159655 | 1.11702 | up | 1.553781 | 0.635783 | 1.553781 | up | 1.112091 | 0.153274 | 1.112091 | up | 0 | 0.049979 | 0.159655 | 0.635783 | 0.153274 | LRRC2 |
| 220189_s_at | 1.03353 | 0.047581 | 1.03353 | up | 1.310969 | 0.390634 | 1.310969 | up | 1.302314 | 0.381078 | 1.302314 | up | 1.51492 | 0.599241 | 1.51492 | up | 0 | 0.047581 | 0.390634 | 0.381078 | 0.599241 | MGAT4B |
| 220450_at | -1.27716 | -0.35294 | 1.277161 | down | -1.36478 | -0.44867 | 1.364778 | down | -1.18053 | -0.23944 | 1.180535 | down | -1.51209 | -0.59654 | 1.51209 | down | 0 | -0.35294 | -0.44867 | -0.23944 | -0.59654 |  |
| 220611_at | 1.443362 | 0.529433 | 1.443362 | up | 1.421319 | 0.507231 | 1.421319 | up | 1.421319 | 0.507231 | 1.421319 | up | 1.608443 | 0.685664 | 1.608443 | up | 0 | 0.529433 | 0.507231 | 0.507231 | 0.685664 | DAB1 |
| 220677_s_at | 1.206648 | 0.271005 | 1.206648 | up | 1.511289 | 0.59578 | 1.511289 | up | 1.206648 | 0.271005 | 1.206648 | up | 1.206648 | 0.271005 | 1.206648 | up | 0 | 0.271005 | 0.59578 | 0.271005 | 0.271005 | ADAMTS8 |
| 220678_at | -1.40355 | -0.48908 | 1.403547 | down | -2.03199 | -1.02289 | 2.031988 | down | -1.40355 | -0.48908 | 1.403547 | down | -1.35358 | -0.43678 | 1.353576 | down | 0 | -0.48908 | -1.02289 | -0.48908 | -0.43678 | FLJ20712 |
| 220937_s_at | 1.270056 | 0.344892 | 1.270056 | up | 1.500965 | 0.58589 | 1.500965 | up | 1.410504 | 0.496211 | 1.410504 | up | 2.009419 | 1.006778 | 2.009419 | up | 0 | 0.344892 | 0.58589 | 0.496211 | 1.006778 | ST6GALNAC4 |
| 221009_s_at | -1.04264 | -0.06024 | 1.042636 | down | 1.106646 | 0.146194 | 1.106646 | up | 1.211536 | 0.276837 | 1.211536 | up | 1.57402 | 0.654454 | 1.57402 | up | 0 | -0.06024 | 0.146194 | 0.276837 | 0.654454 | ANGPTL4 |
| 221110_x_at | -1.6157 | -0.69216 | 1.6157 | down | -1.14649 | -0.19722 | 1.146488 | down | -1.14649 | -0.19722 | 1.146488 | down | -1.14649 | -0.19722 | 1.146488 | down | 0 | -0.69216 | -0.19722 | -0.19722 | -0.19722 | PDE11A |
| 221210_s_at | -1.16924 | -0.22557 | 1.169241 | down | -1.58851 | -0.66767 | 1.588505 | down | -1.16924 | -0.22557 | 1.169241 | down | -1.01755 | -0.0251 | 1.017551 | down | 0 | -0.22557 | -0.66767 | -0.22557 | -0.0251 | NPL |
| 221224_s_at | 1.142934 | 0.192742 | 1.142934 | up | 1.411116 | 0.496837 | 1.411116 | up | 1.503255 | 0.58809 | 1.503255 | up | 1.50652 | 0.591219 | 1.50652 | up | 0 | 0.192742 | 0.496837 | 0.58809 | 0.591219 | DCAKD |
| 221300_at | -1.53088 | -0.61436 | 1.53088 | down | 1.166219 | 0.221838 | 1.166219 | up | -1 | 0 | 1 | down | -1.05217 | -0.07336 | 1.052166 | down | 0 | -0.61436 | 0.221838 | 0 | -0.07336 | NPAP1 |
| 221438_s_at | -1.24514 | -0.31631 | 1.245145 | down | -1.24514 | -0.31631 | 1.245145 | down | -1.42384 | -0.50978 | 1.423836 | down | -1.55377 | -0.63578 | 1.553774 | down | 0 | -0.31631 | -0.31631 | -0.50978 | -0.63578 | TEX12 |
| 221551_x_at | 1.132756 | 0.179837 | 1.132756 | up | 1.535617 | 0.618818 | 1.535617 | up | 1.535617 | 0.618818 | 1.535617 | up | 1.933431 | 0.951163 | 1.933431 | up | 0 | 0.179837 | 0.618818 | 0.618818 | 0.951163 | ST6GALNAC4 |
| 221616_s_at | -1.52583 | -0.6096 | 1.525831 | down | -1 | 0 | 1 | down | 1.28469 | 0.36142 | 1.28469 | up | -1.03479 | -0.04933 | 1.034786 | down | 0 | -0.6096 | 0 | 0.36142 | -0.04933 | TAF9B |
| 221621_at | -1.13126 | -0.17793 | 1.131259 | down | -1.13126 | -0.17793 | 1.131259 | down | -1.13126 | -0.17793 | 1.131259 | down | -1.56555 | -0.64667 | 1.565552 | down | 0 | -0.17793 | -0.17793 | -0.17793 | -0.64667 | SNHG20 |
| 221638_s_at | 1.292483 | 0.370145 | 1.292483 | up | 1.561058 | 0.642524 | 1.561058 | up | 1.479452 | 0.565063 | 1.479452 | up | 1.450707 | 0.536756 | 1.450707 | up | 0 | 0.370145 | 0.642524 | 0.565063 | 0.536756 | STX16 |
| 221745_at | 1.827077 | 0.869537 | 1.827077 | up | 1.719605 | 0.782077 | 1.719605 | up | 2.378226 | 1.249886 | 2.378226 | up | 1.719605 | 0.782077 | 1.719605 | up | 0 | 0.869537 | 0.782077 | 1.249886 | 0.782077 | DCAF7 |
| 221764_at | 1.091044 | 0.125709 | 1.091044 | up | 1.561611 | 0.643035 | 1.561611 | up | 1.373598 | 0.45796 | 1.373598 | up | 1.774662 | 0.827544 | 1.774662 | up | 0 | 0.125709 | 0.643035 | 0.45796 | 0.827544 | R3HDM4 |
| 221905_at | 1.267149 | 0.341587 | 1.267149 | up | 1.300619 | 0.379199 | 1.300619 | up | 1.58162 | 0.661403 | 1.58162 | up | 1.275529 | 0.351095 | 1.275529 | up | 0 | 0.341587 | 0.379199 | 0.661403 | 0.351095 | CYLD |
| 222003_s_at | 1.366732 | 0.45073 | 1.366732 | up | 1.366732 | 0.45073 | 1.366732 | up | 1.366732 | 0.45073 | 1.366732 | up | 1.515051 | 0.599366 | 1.515051 | up | 0 | 0.45073 | 0.45073 | 0.45073 | 0.599366 | DOCK6 |
| 222108_at | -1.02374 | -0.03385 | 1.023742 | down | -1.02709 | -0.03856 | 1.027087 | down | 1.513094 | 0.597502 | 1.513094 | up | 1.214648 | 0.280539 | 1.214648 | up | 0 | -0.03385 | -0.03856 | 0.597502 | 0.280539 | AMIGO2 |
| 222128_at | 1.20046 | 0.263587 | 1.20046 | up | 1.030965 | 0.043995 | 1.030965 | up | 1.502033 | 0.586917 | 1.502033 | up | 1.441671 | 0.527742 | 1.441671 | up | 0 | 0.263587 | 0.043995 | 0.586917 | 0.527742 | LOC105376439 |
| 222264_at | 1.333803 | 0.415546 | 1.333803 | up | 1.354583 | 0.437849 | 1.354583 | up | 1.540177 | 0.623096 | 1.540177 | up | 1.383995 | 0.468838 | 1.383995 | up | 0 | 0.415546 | 0.437849 | 0.623096 | 0.468838 | HNRNPUL2 |
| 222309_at | -1.27278 | -0.34799 | 1.272783 | down | 1.018015 | 0.025759 | 1.018015 | up | -1.09649 | -0.1329 | 1.096492 | down | -1.55187 | -0.63401 | 1.551874 | down | 0 | -0.34799 | 0.025759 | -0.1329 | -0.63401 | C6orf62 |
| 32088_at | -1.156 | -0.20914 | 1.155997 | down | -1.07317 | -0.10188 | 1.073174 | down | -1.05258 | -0.07393 | 1.052576 | down | -1.57365 | -0.65411 | 1.573649 | down | 0 | -0.20914 | -0.10188 | -0.07393 | -0.65411 | BLZF1 |
| 32402_s_at | 1.357091 | 0.440517 | 1.357091 | up | 1.357091 | 0.440517 | 1.357091 | up | 1.357091 | 0.440517 | 1.357091 | up | 1.62199 | 0.697765 | 1.62199 | up | 0 | 0.440517 | 0.440517 | 0.440517 | 0.697765 | SYMPK |
| 66053_at | 2.084187 | 1.059485 | 2.084187 | up | 1.917463 | 0.939199 | 1.917463 | up | 2.327647 | 1.218872 | 2.327647 | up | 2.271145 | 1.18342 | 2.271145 | up | 0 | 1.059485 | 0.939199 | 1.218872 | 1.18342 | HNRNPUL2///HNRNPUL2-BSCL2 |
| 222385_x_at | 1.159515 | 0.213521 | 1.159515 | up | 1.554612 | 0.636555 | 1.554612 | up | 1.499079 | 0.584076 | 1.499079 | up | 1.801745 | 0.849395 | 1.801745 | up | 0 | 0.213521 | 0.636555 | 0.584076 | 0.849395 | SEC61A1 |
| 222509_s_at | -1.5076 | -0.59226 | 1.507603 | down | -1 | 0 | 1 | down | -1 | 0 | 1 | down | 1.211647 | 0.27697 | 1.211647 | up | 0 | -0.59226 | 0 | 0 | 0.27697 | ZNF672 |
| 222598_s_at | 1.053101 | 0.074643 | 1.053101 | up | 1.00585 | 0.008415 | 1.00585 | up | 1.84236 | 0.881555 | 1.84236 | up | 1.201531 | 0.264874 | 1.201531 | up | 0 | 0.074643 | 0.008415 | 0.881555 | 0.264874 | NAV2 |
| 222625_s_at | 1.318871 | 0.399303 | 1.318871 | up | 1.386779 | 0.471738 | 1.386779 | up | 1.405906 | 0.4915 | 1.405906 | up | 1.674378 | 0.743625 | 1.674378 | up | 0 | 0.399303 | 0.471738 | 0.4915 | 0.743625 | NDE1 |
| 222644_s_at | 1.252724 | 0.325068 | 1.252724 | up | 1.324755 | 0.405725 | 1.324755 | up | 1.284414 | 0.36111 | 1.284414 | up | 1.616272 | 0.69267 | 1.616272 | up | 0 | 0.325068 | 0.405725 | 0.36111 | 0.69267 | COLGALT1 |
| 222645_s_at | -1.12755 | -0.17319 | 1.127547 | down | 1.407455 | 0.493088 | 1.407455 | up | 1.156728 | 0.21005 | 1.156728 | up | 1.650578 | 0.722971 | 1.650578 | up | 0 | -0.17319 | 0.493088 | 0.21005 | 0.722971 | KCTD5 |
| 222655_s_at | 1.017666 | 0.025265 | 1.017666 | up | 1.186058 | 0.246175 | 1.186058 | up | 1.186058 | 0.246175 | 1.186058 | up | 1.582135 | 0.661872 | 1.582135 | up | 0 | 0.025265 | 0.246175 | 0.246175 | 0.661872 | IMPAD1 |
| 222905_s_at | -1.53084 | -0.61432 | 1.530839 | down | 1.027295 | 0.03885 | 1.027295 | up | -1.09761 | -0.13437 | 1.09761 | down | -1.22898 | -0.29746 | 1.228977 | down | 0 | -0.61432 | 0.03885 | -0.13437 | -0.29746 | TMEM143 |
| 222987_s_at | 1.110645 | 0.151398 | 1.110645 | up | 1.255409 | 0.328157 | 1.255409 | up | 1.181514 | 0.240636 | 1.181514 | up | 1.545156 | 0.627753 | 1.545156 | up | 0 | 0.151398 | 0.328157 | 0.240636 | 0.627753 | TMEM9 |
| 223131_s_at | 1.419359 | 0.50524 | 1.419359 | up | 1.453926 | 0.539954 | 1.453926 | up | 1.831026 | 0.872652 | 1.831026 | up | 1.668187 | 0.738281 | 1.668187 | up | 0 | 0.50524 | 0.539954 | 0.872652 | 0.738281 | TRIM8 |
| 223142_s_at | 1.018508 | 0.026457 | 1.018508 | up | 1.574578 | 0.654965 | 1.574578 | up | 1.269956 | 0.344778 | 1.269956 | up | 1.441628 | 0.527699 | 1.441628 | up | 0 | 0.026457 | 0.654965 | 0.344778 | 0.527699 | UCK1 |
| 223143_s_at | 1.07308 | 0.101757 | 1.07308 | up | 1.522104 | 0.606067 | 1.522104 | up | 1.256058 | 0.328903 | 1.256058 | up | 1.698209 | 0.764014 | 1.698209 | up | 0 | 0.101757 | 0.606067 | 0.328903 | 0.764014 | AKIRIN2 |
| 223333_s_at | 1.111989 | 0.153143 | 1.111989 | up | -1.16464 | -0.21989 | 1.164645 | down | 1.451764 | 0.537807 | 1.451764 | up | 1.676022 | 0.745041 | 1.676022 | up | 0 | 0.153143 | -0.21989 | 0.537807 | 0.745041 | ANGPTL4 |
| 223537_s_at | 1.015606 | 0.022341 | 1.015606 | up | 1.06819 | 0.095169 | 1.06819 | up | 1.06819 | 0.095169 | 1.06819 | up | 1.55735 | 0.639093 | 1.55735 | up | 0 | 0.022341 | 0.095169 | 0.095169 | 0.639093 | WNT5B |
| 223588_at | -1.16827 | -0.22437 | 1.168265 | down | -1.02112 | -0.03016 | 1.021123 | down | -1.13437 | -0.18189 | 1.134372 | down | -1.55978 | -0.64134 | 1.559781 | down | 0 | -0.22437 | -0.03016 | -0.18189 | -0.64134 | THAP2 |
| 223674_s_at | 1.280808 | 0.357054 | 1.280808 | up | 1.280808 | 0.357054 | 1.280808 | up | 1.280808 | 0.357054 | 1.280808 | up | 1.631642 | 0.706325 | 1.631642 | up | 0 | 0.357054 | 0.357054 | 0.357054 | 0.706325 | CDC42SE1 |
| 223698_at | -1.21762 | -0.28406 | 1.21762 | down | -1.59613 | -0.67458 | 1.596132 | down | -1.21762 | -0.28406 | 1.21762 | down | -1.22782 | -0.2961 | 1.227819 | down | 0 | -0.28406 | -0.67458 | -0.28406 | -0.2961 | SLC25A36 |
| 223725_at | -1.12325 | -0.16769 | 1.123255 | down | -1.28569 | -0.36255 | 1.285695 | down | -1.28569 | -0.36255 | 1.285695 | down | -1.76264 | -0.81774 | 1.762641 | down | 0 | -0.16769 | -0.36255 | -0.36255 | -0.81774 | LINC00852 |
| 223789_s_at | -1.539 | -0.622 | 1.539003 | down | 1.295275 | 0.373258 | 1.295275 | up | 1.286734 | 0.363714 | 1.286734 | up | 1.492482 | 0.577714 | 1.492482 | up | 0 | -0.622 | 0.373258 | 0.363714 | 0.577714 | GTPBP2 |
| 223940_x_at | 2.105252 | 1.073993 | 2.105252 | up | 1.120255 | 0.163827 | 1.120255 | up | -1.16659 | -0.22229 | 1.166586 | down | -1.15193 | -0.20406 | 1.151932 | down | 0 | 1.073993 | 0.163827 | -0.22229 | -0.20406 | MALAT1 |
| 224102_at | 1.576131 | 0.656387 | 1.576131 | up | 1.130903 | 0.177475 | 1.130903 | up | 1.212264 | 0.277704 | 1.212264 | up | 1.130903 | 0.177475 | 1.130903 | up | 0 | 0.656387 | 0.177475 | 0.277704 | 0.177475 | P2RY12 |
| 224192_at | -1.29825 | -0.37657 | 1.298251 | down | -1.69524 | -0.76149 | 1.695244 | down | -1.22454 | -0.29224 | 1.224538 | down | -1.22454 | -0.29224 | 1.224538 | down | 0 | -0.37657 | -0.76149 | -0.29224 | -0.29224 | FCRL2 |
| 224372_at | 1.745964 | 0.804024 | 1.745964 | up | 1.133198 | 0.1804 | 1.133198 | up | 1.549553 | 0.631852 | 1.549553 | up | -1.08635 | -0.11949 | 1.086348 | down | 0 | 0.804024 | 0.1804 | 0.631852 | -0.11949 | ND4 |
| 224373_s_at | 1.703084 | 0.768149 | 1.703084 | up | 1.144819 | 0.195119 | 1.144819 | up | 1.540842 | 0.623719 | 1.540842 | up | 1.0164 | 0.023468 | 1.0164 | up | 0 | 0.768149 | 0.195119 | 0.623719 | 0.023468 | ND4 |
| 224559_at | 1.299974 | 0.378483 | 1.299974 | up | 1.112339 | 0.153596 | 1.112339 | up | -1.66218 | -0.73308 | 1.662178 | down | 1.079423 | 0.110261 | 1.079423 | up | 0 | 0.378483 | 0.153596 | -0.73308 | 0.110261 | MALAT1 |
| 224563_at | 1.175131 | 0.232822 | 1.175131 | up | 1.179753 | 0.238485 | 1.179753 | up | 2.021557 | 1.015467 | 2.021557 | up | 1.179753 | 0.238485 | 1.179753 | up | 0 | 0.232822 | 0.238485 | 1.015467 | 0.238485 | WASF2 |
| 224566_at | 1.833052 | 0.874248 | 1.833052 | up | 1.20966 | 0.274601 | 1.20966 | up | 1.34039 | 0.422652 | 1.34039 | up | 1.127814 | 0.173529 | 1.127814 | up | 0 | 0.874248 | 0.274601 | 0.422652 | 0.173529 | NEAT1 |
| 224568_x_at | 2.241655 | 1.164564 | 2.241655 | up | 1.4935 | 0.578697 | 1.4935 | up | 1.057089 | 0.080096 | 1.057089 | up | 1.221918 | 0.289147 | 1.221918 | up | 0 | 1.164564 | 0.578697 | 0.080096 | 0.289147 | MALAT1 |
| 224646_x_at | 1.046979 | 0.066233 | 1.046979 | up | 1.535327 | 0.618546 | 1.535327 | up | 1.112898 | 0.154322 | 1.112898 | up | -1.23414 | -0.30351 | 1.234141 | down | 0 | 0.066233 | 0.618546 | 0.154322 | -0.30351 | H19///LOC102724852 |
| 224730_at | 1.359184 | 0.44274 | 1.359184 | up | 1.393342 | 0.478549 | 1.393342 | up | 2.339539 | 1.226224 | 2.339539 | up | 1.39368 | 0.478899 | 1.39368 | up | 0 | 0.44274 | 0.478549 | 1.226224 | 0.478899 | DCAF7 |
| 224770_s_at | 1.210923 | 0.276107 | 1.210923 | up | 1.074387 | 0.103514 | 1.074387 | up | 1.524206 | 0.608058 | 1.524206 | up | 1.354443 | 0.437699 | 1.354443 | up | 0 | 0.276107 | 0.103514 | 0.608058 | 0.437699 | NAV1 |
| 224771_at | 1.283482 | 0.360063 | 1.283482 | up | 1.283482 | 0.360063 | 1.283482 | up | 2.009612 | 1.006917 | 2.009612 | up | 1.349889 | 0.43284 | 1.349889 | up | 0 | 0.360063 | 0.360063 | 1.006917 | 0.43284 | NAV1 |
| 224920_x_at | 1.220903 | 0.287949 | 1.220903 | up | 1.10618 | 0.145586 | 1.10618 | up | 1.579241 | 0.659231 | 1.579241 | up | 1.22885 | 0.297309 | 1.22885 | up | 0 | 0.287949 | 0.145586 | 0.659231 | 0.297309 | MYADM |
| 224982_at | 1.238258 | 0.308311 | 1.238258 | up | 1.726925 | 0.788206 | 1.726925 | up | 1.74455 | 0.802855 | 1.74455 | up | 2.000771 | 1.000556 | 2.000771 | up | 0 | 0.308311 | 0.788206 | 0.802855 | 1.000556 | AKT1S1 |
| 225089_at | 1.069636 | 0.09712 | 1.069636 | up | 1.191058 | 0.252244 | 1.191058 | up | 1.883296 | 0.91326 | 1.883296 | up | 1.468969 | 0.554804 | 1.468969 | up | 0 | 0.09712 | 0.252244 | 0.91326 | 0.554804 | USP40 |
| 225097_at | 1.345715 | 0.428373 | 1.345715 | up | 1.178542 | 0.237003 | 1.178542 | up | 1.653936 | 0.725904 | 1.653936 | up | 1.443959 | 0.530029 | 1.443959 | up | 0 | 0.428373 | 0.237003 | 0.725904 | 0.530029 | HIPK2 |
| 225119_at | 1.274974 | 0.350468 | 1.274974 | up | 1.224206 | 0.291846 | 1.224206 | up | 1.622867 | 0.698545 | 1.622867 | up | 1.274974 | 0.350468 | 1.274974 | up | 0 | 0.350468 | 0.291846 | 0.698545 | 0.350468 | CHMP4B |
| 225239_at | 1.67777 | 0.746545 | 1.67777 | up | 1.02632 | 0.037481 | 1.02632 | up | 1.58657 | 0.665911 | 1.58657 | up | 1.379917 | 0.464581 | 1.379917 | up | 0 | 0.746545 | 0.037481 | 0.665911 | 0.464581 | NEAT1 |
| 225428_s_at | 1.15866 | 0.212457 | 1.15866 | up | 1.458946 | 0.544927 | 1.458946 | up | 1.570088 | 0.650846 | 1.570088 | up | 1.443984 | 0.530055 | 1.443984 | up | 0 | 0.212457 | 0.544927 | 0.650846 | 0.530055 | DDX54 |
| 225452_at | 1.083486 | 0.115681 | 1.083486 | up | 1.169775 | 0.226231 | 1.169775 | up | 1.5633 | 0.644595 | 1.5633 | up | 1.2197 | 0.286526 | 1.2197 | up | 0 | 0.115681 | 0.226231 | 0.644595 | 0.286526 | MED1 |
| 225453_x_at | -1.51474 | -0.59907 | 1.514744 | down | -1.16531 | -0.22072 | 1.165313 | down | -1.17264 | -0.22976 | 1.172641 | down | -1.03016 | -0.04287 | 1.030158 | down | 0 | -0.59907 | -0.22072 | -0.22976 | -0.04287 | CCDC124 |
| 225454_at | 1.074181 | 0.103237 | 1.074181 | up | 1.233203 | 0.30241 | 1.233203 | up | 1.25514 | 0.327848 | 1.25514 | up | 1.514731 | 0.599062 | 1.514731 | up | 0 | 0.103237 | 0.30241 | 0.327848 | 0.599062 | CCDC124 |
| 225605_at | 1.264917 | 0.339042 | 1.264917 | up | 1.240505 | 0.310927 | 1.240505 | up | 1.240505 | 0.310927 | 1.240505 | up | 1.603517 | 0.681239 | 1.603517 | up | 0 | 0.339042 | 0.310927 | 0.310927 | 0.681239 | TP53I13 |
| 225767_at | 1.313252 | 0.393144 | 1.313252 | up | 1.395654 | 0.480942 | 1.395654 | up | 1.634297 | 0.70867 | 1.634297 | up | 1.336786 | 0.418768 | 1.336786 | up | 0 | 0.393144 | 0.480942 | 0.70867 | 0.418768 | RNA45S5 |
| 225786_at | 1.556768 | 0.638554 | 1.556768 | up | 1.274059 | 0.349432 | 1.274059 | up | 1.780525 | 0.832303 | 1.780525 | up | 1.208893 | 0.273687 | 1.208893 | up | 0 | 0.638554 | 0.349432 | 0.832303 | 0.273687 | HNRNPU |
| 225818_s_at | 1.164575 | 0.219804 | 1.164575 | up | 1.334782 | 0.416604 | 1.334782 | up | 1.583478 | 0.663096 | 1.583478 | up | 1.220244 | 0.287169 | 1.220244 | up | 0 | 0.219804 | 0.416604 | 0.663096 | 0.287169 | TBRG1 |
| 225828_at | 1.20646 | 0.27078 | 1.20646 | up | 1.745866 | 0.803943 | 1.745866 | up | 1.607664 | 0.684966 | 1.607664 | up | 1.82201 | 0.865531 | 1.82201 | up | 0 | 0.27078 | 0.803943 | 0.684966 | 0.865531 | DAGLB |
| 225929_s_at | 1.182497 | 0.241837 | 1.182497 | up | -1.03098 | -0.04401 | 1.030979 | down | 1.100266 | 0.137853 | 1.100266 | up | 1.562177 | 0.643558 | 1.562177 | up | 0 | 0.241837 | -0.04401 | 0.137853 | 0.643558 | RNF213 |
| 226085_at | 1.211903 | 0.277274 | 1.211903 | up | 1.246861 | 0.318301 | 1.246861 | up | 1.613521 | 0.690212 | 1.613521 | up | 1.606864 | 0.684248 | 1.606864 | up | 0 | 0.277274 | 0.318301 | 0.690212 | 0.684248 | CBX5 |
| 226305_at | 1.09238 | 0.127475 | 1.09238 | up | 1.245649 | 0.316897 | 1.245649 | up | 1.188294 | 0.248892 | 1.188294 | up | 1.603743 | 0.681443 | 1.603743 | up | 0 | 0.127475 | 0.316897 | 0.248892 | 0.681443 | LYNX1 |
| 226663_at | 2.221733 | 1.151686 | 2.221733 | up | 1.292343 | 0.369989 | 1.292343 | up | 1.569289 | 0.650111 | 1.569289 | up | 1.340397 | 0.422661 | 1.340397 | up | 0 | 1.151686 | 0.369989 | 0.650111 | 0.422661 | ANKRD10///ANKRD10-IT1 |
| 226788_at | 1.009962 | 0.014301 | 1.009962 | up | -1.54537 | -0.62795 | 1.545368 | down | -1.16217 | -0.21682 | 1.162169 | down | -1.16217 | -0.21682 | 1.162169 | down | 0 | 0.014301 | -0.62795 | -0.21682 | -0.21682 | CENPT |
| 226983_at | 1.308958 | 0.388419 | 1.308958 | up | 1.373963 | 0.458344 | 1.373963 | up | 1.373963 | 0.458344 | 1.373963 | up | 1.810805 | 0.856631 | 1.810805 | up | 0 | 0.388419 | 0.458344 | 0.458344 | 0.856631 | ZNF777 |
| 226991_at | 1.070838 | 0.098741 | 1.070838 | up | 1.280537 | 0.356749 | 1.280537 | up | 2.073032 | 1.051742 | 2.073032 | up | 1.538263 | 0.621302 | 1.538263 | up | 0 | 0.098741 | 0.356749 | 1.051742 | 0.621302 | NFATC2 |
| 227055_at | -1.05122 | -0.07206 | 1.051218 | down | -1.19416 | -0.256 | 1.194162 | down | -1.23384 | -0.30316 | 1.233844 | down | -1.56417 | -0.6454 | 1.564174 | down | 0 | -0.07206 | -0.256 | -0.30316 | -0.6454 | METTL7B |
| 227070_at | -1.12229 | -0.16645 | 1.122291 | down | -1.00493 | -0.00709 | 1.004926 | down | -1.0522 | -0.07341 | 1.052199 | down | -1.53036 | -0.61387 | 1.530364 | down | 0 | -0.16645 | -0.00709 | -0.07341 | -0.61387 | GLT8D2 |
| 227082_at | 1.290004 | 0.367375 | 1.290004 | up | 1.290004 | 0.367375 | 1.290004 | up | 1.490028 | 0.57534 | 1.490028 | up | 1.511795 | 0.596262 | 1.511795 | up | 0 | 0.367375 | 0.367375 | 0.57534 | 0.596262 | ZBTB20 |
| 227121_at | 1.067294 | 0.093957 | 1.067294 | up | 1.210789 | 0.275947 | 1.210789 | up | 1.292039 | 0.369649 | 1.292039 | up | 1.746796 | 0.804711 | 1.746796 | up | 0 | 0.093957 | 0.275947 | 0.369649 | 0.804711 | ZBTB20 |
| 227269_s_at | -1.50312 | -0.58796 | 1.503119 | down | -1.1065 | -0.146 | 1.106496 | down | -1.16277 | -0.21756 | 1.162767 | down | -1.11939 | -0.16271 | 1.119388 | down | 0 | -0.58796 | -0.146 | -0.21756 | -0.16271 | RAB40C |
| 227300_at | 1.309681 | 0.389215 | 1.309681 | up | 1.599303 | 0.677444 | 1.599303 | up | 1.559663 | 0.641234 | 1.559663 | up | 1.901171 | 0.926888 | 1.901171 | up | 0 | 0.389215 | 0.677444 | 0.641234 | 0.926888 | TMEM119 |
| 227404_s_at | 1.081002 | 0.112369 | 1.081002 | up | -1.20433 | -0.26823 | 1.204326 | down | 1.522856 | 0.606779 | 1.522856 | up | -1 | 0 | 1 | down | 0 | 0.112369 | -0.26823 | 0.606779 | 0 | EGR1 |
| 227458_at | -1.11296 | -0.1544 | 1.112956 | down | -1.45329 | -0.53932 | 1.453287 | down | 1.138818 | 0.187537 | 1.138818 | up | -1.71406 | -0.77742 | 1.714057 | down | 0 | -0.1544 | -0.53932 | 0.187537 | -0.77742 | CD274 |
| 227781_x_at | 1.289099 | 0.366363 | 1.289099 | up | 1.517549 | 0.601743 | 1.517549 | up | 1.289099 | 0.366363 | 1.289099 | up | 1.289099 | 0.366363 | 1.289099 | up | 0 | 0.366363 | 0.601743 | 0.366363 | 0.366363 | FAM57B |
| 227855_at | -1.21166 | -0.27698 | 1.211658 | down | -1.51524 | -0.59955 | 1.515244 | down | -1.2779 | -0.35377 | 1.2779 | down | -1.21166 | -0.27698 | 1.211658 | down | 0 | -0.27698 | -0.59955 | -0.35377 | -0.27698 | ARHGEF40 |
| 227948_at | 1.220339 | 0.287282 | 1.220339 | up | 1.150652 | 0.202451 | 1.150652 | up | 1.531858 | 0.615283 | 1.531858 | up | 1.148912 | 0.200268 | 1.148912 | up | 0 | 0.287282 | 0.202451 | 0.615283 | 0.200268 | FGD4 |
| 228060_at | -1.44224 | -0.52831 | 1.44224 | down | -1.32241 | -0.40317 | 1.322413 | down | -1.57776 | -0.65788 | 1.577759 | down | -1.32166 | -0.40235 | 1.321656 | down | 0 | -0.52831 | -0.40317 | -0.65788 | -0.40235 | SLC35F1 |
| 228074_at | 1.239898 | 0.310222 | 1.239898 | up | 1.422513 | 0.508441 | 1.422513 | up | 1.336333 | 0.41828 | 1.336333 | up | 1.512233 | 0.596681 | 1.512233 | up | 0 | 0.310222 | 0.508441 | 0.41828 | 0.596681 | ITPRIPL2 |
| 228079_at | -1.25649 | -0.3294 | 1.256494 | down | -1.50215 | -0.58703 | 1.502152 | down | -1.50215 | -0.58703 | 1.502152 | down | -1.70938 | -0.77347 | 1.709378 | down | 0 | -0.3294 | -0.58703 | -0.58703 | -0.77347 | C3orf58 |
| 228128_x_at | 1.050244 | 0.070724 | 1.050244 | up | 1.258222 | 0.331387 | 1.258222 | up | 1.55687 | 0.638648 | 1.55687 | up | 1.398448 | 0.483827 | 1.398448 | up | 0 | 0.070724 | 0.331387 | 0.638648 | 0.483827 | PAPPA |
| 228156_at | 1.806034 | 0.852825 | 1.806034 | up | 1.588119 | 0.667319 | 1.588119 | up | 1.644096 | 0.717294 | 1.644096 | up | 1.666688 | 0.736984 | 1.666688 | up | 0 | 0.852825 | 0.667319 | 0.717294 | 0.736984 | ZNF782 |
| 228262_at | -1.29357 | -0.37136 | 1.293568 | down | -1.29357 | -0.37136 | 1.293568 | down | -1.29357 | -0.37136 | 1.293568 | down | -1.54346 | -0.62617 | 1.543457 | down | 0 | -0.37136 | -0.37136 | -0.37136 | -0.62617 | MAP7D2 |
| 228442_at | -1.00113 | -0.00164 | 1.001135 | down | -1.09992 | -0.1374 | 1.099925 | down | 1.663063 | 0.733842 | 1.663063 | up | 1.185472 | 0.245461 | 1.185472 | up | 0 | -0.00164 | -0.1374 | 0.733842 | 0.245461 | NFATC2 |
| 228512_at | 1.431144 | 0.517169 | 1.431144 | up | 1.265234 | 0.339405 | 1.265234 | up | 1.533584 | 0.616907 | 1.533584 | up | 1.281586 | 0.357931 | 1.281586 | up | 0 | 0.517169 | 0.339405 | 0.616907 | 0.357931 | PTCD3 |
| 228599_at | -1.41799 | -0.50384 | 1.417988 | down | -1.53523 | -0.61846 | 1.535235 | down | -1.41799 | -0.50384 | 1.417988 | down | -1.41799 | -0.50384 | 1.417988 | down | 0 | -0.50384 | -0.61846 | -0.50384 | -0.50384 | MS4A1 |
| 228754_at | 1.072686 | 0.101227 | 1.072686 | up | 1.099031 | 0.136232 | 1.099031 | up | 1.46638 | 0.552259 | 1.46638 | up | 1.609363 | 0.68649 | 1.609363 | up | 0 | 0.101227 | 0.136232 | 0.552259 | 0.68649 | SLC6A6 |
| 228779_at | 1.851013 | 0.888315 | 1.851013 | up | 1.227601 | 0.295842 | 1.227601 | up | 1.227601 | 0.295842 | 1.227601 | up | 1.289055 | 0.366314 | 1.289055 | up | 0 | 0.888315 | 0.295842 | 0.295842 | 0.366314 | LOC146880 |
| 228896_at | -1.62855 | -0.70359 | 1.628548 | down | -1.355 | -0.43829 | 1.355 | down | -1.30617 | -0.38534 | 1.306167 | down | -1.355 | -0.43829 | 1.355 | down | 0 | -0.70359 | -0.43829 | -0.38534 | -0.43829 |  |
| 228937_at | 1.054915 | 0.077127 | 1.054915 | up | 1.25263 | 0.32496 | 1.25263 | up | 1.558211 | 0.639891 | 1.558211 | up | 1.135834 | 0.183752 | 1.135834 | up | 0 | 0.077127 | 0.32496 | 0.639891 | 0.183752 | LACC1 |
| 228948_at | -1.18429 | -0.24402 | 1.184285 | down | -1.57378 | -0.65424 | 1.573782 | down | -1.18429 | -0.24402 | 1.184285 | down | -1.0589 | -0.08257 | 1.058901 | down | 0 | -0.24402 | -0.65424 | -0.24402 | -0.08257 | EPHA4 |
| 228955_at | 1.108486 | 0.148591 | 1.108486 | up | -1.01337 | -0.01916 | 1.013372 | down | 1.143384 | 0.19331 | 1.143384 | up | 1.608954 | 0.686123 | 1.608954 | up | 0 | 0.148591 | -0.01916 | 0.19331 | 0.686123 | LRP8 |
| 228982_s_at | 1.642887 | 0.716233 | 1.642887 | up | 1.212264 | 0.277704 | 1.212264 | up | 1.212264 | 0.277704 | 1.212264 | up | 1.432682 | 0.518719 | 1.432682 | up | 0 | 0.716233 | 0.277704 | 0.277704 | 0.518719 | USP42 |
| 229038_at | 1.156569 | 0.209852 | 1.156569 | up | 1.148486 | 0.199733 | 1.148486 | up | 1.573574 | 0.654045 | 1.573574 | up | 1.109845 | 0.150358 | 1.109845 | up | 0 | 0.209852 | 0.199733 | 0.654045 | 0.150358 | CWF19L1 |
| 229040_at | 1.267601 | 0.3421 | 1.267601 | up | 1.106582 | 0.146111 | 1.106582 | up | 1.027437 | 0.03905 | 1.027437 | up | 1.655333 | 0.727121 | 1.655333 | up | 0 | 0.3421 | 0.146111 | 0.03905 | 0.727121 | ITGB2-AS1 |
| 229070_at | -1.01028 | -0.01476 | 1.010284 | down | 1.0531 | 0.074643 | 1.0531 | up | 1.247399 | 0.318923 | 1.247399 | up | -1.53023 | -0.61375 | 1.530231 | down | 0 | -0.01476 | 0.074643 | 0.318923 | -0.61375 | ADTRP |
| 229115_at | 1.063077 | 0.088245 | 1.063077 | up | -1.16307 | -0.21793 | 1.163067 | down | 1.572884 | 0.653412 | 1.572884 | up | 1.498591 | 0.583607 | 1.498591 | up | 0 | 0.088245 | -0.21793 | 0.653412 | 0.583607 | DYNC1H1 |
| 229206_at | 1.149099 | 0.200504 | 1.149099 | up | 1.149099 | 0.200504 | 1.149099 | up | 1.167286 | 0.223159 | 1.167286 | up | 1.621044 | 0.696923 | 1.621044 | up | 0 | 0.200504 | 0.200504 | 0.223159 | 0.696923 |  |
| 229273_at | 1.522612 | 0.606548 | 1.522612 | up | 1.344048 | 0.426585 | 1.344048 | up | 1.472967 | 0.558725 | 1.472967 | up | 1.304415 | 0.383403 | 1.304415 | up | 0 | 0.606548 | 0.426585 | 0.558725 | 0.383403 | SALL1 |
| 229346_at | 1.642532 | 0.715921 | 1.642532 | up | 1.342878 | 0.425328 | 1.342878 | up | 1.342878 | 0.425328 | 1.342878 | up | 1.335338 | 0.417204 | 1.335338 | up | 0 | 0.715921 | 0.425328 | 0.425328 | 0.417204 | NES |
| 229366_at | 1.622867 | 0.698545 | 1.622867 | up | -1 | 0 | 1 | down | -1 | 0 | 1 | down | -1 | 0 | 1 | down | 0 | 0.698545 | 0 | 0 | 0 |  |
| 229547_s_at | 1.366148 | 0.450114 | 1.366148 | up | 1.366148 | 0.450114 | 1.366148 | up | 1.366148 | 0.450114 | 1.366148 | up | 1.542528 | 0.625297 | 1.542528 | up | 0 | 0.450114 | 0.450114 | 0.450114 | 0.625297 | WNK2 |
| 229550_at | -1.46868 | -0.55452 | 1.46868 | down | -1.66608 | -0.73646 | 1.666083 | down | -1.74581 | -0.8039 | 1.745812 | down | -1.32276 | -0.40355 | 1.322756 | down | 0 | -0.55452 | -0.73646 | -0.8039 | -0.40355 | UNC79 |
| 229625_at | -1.71293 | -0.77646 | 1.712927 | down | -1.36009 | -0.4437 | 1.360092 | down | -1.36009 | -0.4437 | 1.360092 | down | -1.27388 | -0.34923 | 1.273881 | down | 0 | -0.77646 | -0.4437 | -0.4437 | -0.34923 | GBP5 |
| 229633_at | 1.696557 | 0.76261 | 1.696557 | up | 1.301806 | 0.380515 | 1.301806 | up | 1.274805 | 0.350276 | 1.274805 | up | 1.133022 | 0.180175 | 1.133022 | up | 0 | 0.76261 | 0.380515 | 0.350276 | 0.180175 | INTS10 |
| 229733_s_at | -1.0208 | -0.0297 | 1.0208 | down | 1.242328 | 0.313046 | 1.242328 | up | 1.258129 | 0.33128 | 1.258129 | up | 1.523872 | 0.607742 | 1.523872 | up | 0 | -0.0297 | 0.313046 | 0.33128 | 0.607742 |  |
| 229756_at | -1.55067 | -0.63289 | 1.550665 | down | -1.06474 | -0.0905 | 1.064743 | down | -1.39637 | -0.48168 | 1.39637 | down | -1.44586 | -0.53193 | 1.445863 | down | 0 | -0.63289 | -0.0905 | -0.48168 | -0.53193 | ID2-AS1 |
| 229916_at | 1.563392 | 0.64468 | 1.563392 | up | -1 | 0 | 1 | down | -1 | 0 | 1 | down | -1.0676 | -0.09437 | 1.067599 | down | 0 | 0.64468 | 0 | 0 | -0.09437 | ENPP6 |
| 230221_at | 1.663525 | 0.734244 | 1.663525 | up | 1.299295 | 0.377729 | 1.299295 | up | 1.299295 | 0.377729 | 1.299295 | up | 1.337643 | 0.419693 | 1.337643 | up | 0 | 0.734244 | 0.377729 | 0.377729 | 0.419693 | ABHD16A |
| 230229_at | 1.754415 | 0.81099 | 1.754415 | up | -1.04942 | -0.06959 | 1.049421 | down | 1.200159 | 0.263225 | 1.200159 | up | 1.172853 | 0.230022 | 1.172853 | up | 0 | 0.81099 | -0.06959 | 0.263225 | 0.230022 | DLG1 |
| 230340_s_at | 1.362139 | 0.445874 | 1.362139 | up | 1.129806 | 0.176075 | 1.129806 | up | 1.574251 | 0.654665 | 1.574251 | up | 1.09908 | 0.136297 | 1.09908 | up | 0 | 0.445874 | 0.176075 | 0.654665 | 0.136297 | WASL |
| 230372_at | 1.006941 | 0.009979 | 1.006941 | up | -1.4728 | -0.55856 | 1.472801 | down | -1.03668 | -0.05198 | 1.036683 | down | -1.54735 | -0.6298 | 1.547351 | down | 0 | 0.009979 | -0.55856 | -0.05198 | -0.6298 | HAS2 |
| 230395_at | 1.523838 | 0.60771 | 1.523838 | up | -1.07936 | -0.11018 | 1.079359 | down | -1.13779 | -0.18623 | 1.13779 | down | -1.00148 | -0.00213 | 1.001475 | down | 0 | 0.60771 | -0.11018 | -0.18623 | -0.00213 | METTL9 |
| 230493_at | 1.152116 | 0.204286 | 1.152116 | up | 1.355915 | 0.439267 | 1.355915 | up | 2.81076 | 1.49096 | 2.81076 | up | 1.591194 | 0.67011 | 1.591194 | up | 0 | 0.204286 | 0.439267 | 1.49096 | 0.67011 | SHISA2 |
| 230559_x_at | -1.10824 | -0.14827 | 1.108241 | down | 1.146017 | 0.196628 | 1.146017 | up | 1.621142 | 0.697011 | 1.621142 | up | 1.146017 | 0.196628 | 1.146017 | up | 0 | -0.14827 | 0.196628 | 0.697011 | 0.196628 | FGD4 |
| 230646_at | -1.31798 | -0.39833 | 1.317977 | down | -1.34988 | -0.43283 | 1.349883 | down | -1.54402 | -0.62669 | 1.544017 | down | -1.34988 | -0.43283 | 1.349883 | down | 0 | -0.39833 | -0.43283 | -0.62669 | -0.43283 | FNDC5 |
| 230704_s_at | -1.08541 | -0.11824 | 1.08541 | down | -1.23371 | -0.303 | 1.233706 | down | -1.53692 | -0.62004 | 1.536916 | down | -1.13735 | -0.18568 | 1.13735 | down | 0 | -0.11824 | -0.303 | -0.62004 | -0.18568 | ITGB4 |
| 230748_at | 1.171503 | 0.228361 | 1.171503 | up | 1.325848 | 0.406916 | 1.325848 | up | 1.631078 | 0.705826 | 1.631078 | up | 1.431547 | 0.517575 | 1.431547 | up | 0 | 0.228361 | 0.406916 | 0.705826 | 0.517575 | SLC16A6 |
| 230779_at | 1.144735 | 0.195014 | 1.144735 | up | 1.323456 | 0.40431 | 1.323456 | up | 1.355174 | 0.438478 | 1.355174 | up | 1.567947 | 0.648877 | 1.567947 | up | 0 | 0.195014 | 0.40431 | 0.438478 | 0.648877 | TNRC6B |
| 230884_s_at | 1.372849 | 0.457173 | 1.372849 | up | 1.508726 | 0.59333 | 1.508726 | up | 1.447692 | 0.533754 | 1.447692 | up | 1.952403 | 0.965251 | 1.952403 | up | 0 | 0.457173 | 0.59333 | 0.533754 | 0.965251 | SPG7 |
| 230918_at | 1.179814 | 0.23856 | 1.179814 | up | -1.06919 | -0.09652 | 1.069192 | down | 1.187046 | 0.247375 | 1.187046 | up | 1.538906 | 0.621905 | 1.538906 | up | 0 | 0.23856 | -0.09652 | 0.247375 | 0.621905 | GALK2 |
| 231169_at | 1.370794 | 0.455012 | 1.370794 | up | 1.370794 | 0.455012 | 1.370794 | up | 1.370794 | 0.455012 | 1.370794 | up | 1.620037 | 0.696027 | 1.620037 | up | 0 | 0.455012 | 0.455012 | 0.455012 | 0.696027 |  |
| 231292_at | -1.19632 | -0.2586 | 1.196321 | down | -1.36116 | -0.44484 | 1.361159 | down | -1.36116 | -0.44484 | 1.361159 | down | -1.79554 | -0.84442 | 1.79554 | down | 0 | -0.2586 | -0.44484 | -0.44484 | -0.84442 | EID3 |
| 231427_at | 1.22975 | 0.298365 | 1.22975 | up | -1.08111 | -0.11252 | 1.081113 | down | -1.14789 | -0.19898 | 1.14789 | down | 1.626008 | 0.701335 | 1.626008 | up | 0 | 0.298365 | -0.11252 | -0.19898 | 0.701335 | LOC284648 |
| 231486_x_at | 1.11804 | 0.160972 | 1.11804 | up | -1.03398 | -0.0482 | 1.033977 | down | 1.11636 | 0.158802 | 1.11636 | up | 1.993399 | 0.99523 | 1.993399 | up | 0 | 0.160972 | -0.0482 | 0.158802 | 0.99523 |  |
| 231504_at | 1.599871 | 0.677955 | 1.599871 | up | -1.0829 | -0.1149 | 1.082899 | down | 1.035163 | 0.049859 | 1.035163 | up | 1.051646 | 0.072649 | 1.051646 | up | 0 | 0.677955 | -0.1149 | 0.049859 | 0.072649 | CCDC148 |
| 231533_at | -1.51067 | -0.59519 | 1.510671 | down | -1.19069 | -0.2518 | 1.190694 | down | -1.13027 | -0.17667 | 1.130272 | down | 1.023806 | 0.033942 | 1.023806 | up | 0 | -0.59519 | -0.2518 | -0.17667 | 0.033942 |  |
| 231790_at | 1.60682 | 0.684208 | 1.60682 | up | 1.071527 | 0.099669 | 1.071527 | up | 1.058335 | 0.081797 | 1.058335 | up | -1.16961 | -0.22603 | 1.169609 | down | 0 | 0.684208 | 0.099669 | 0.081797 | -0.22603 | DMGDH |
| 231926_at | -1.16253 | -0.21727 | 1.162531 | down | -1.19993 | -0.26295 | 1.199926 | down | -1.23096 | -0.29978 | 1.230955 | down | -1.60765 | -0.68495 | 1.607647 | down | 0 | -0.21727 | -0.26295 | -0.29978 | -0.68495 | EPS15L1 |
| 231929_at | 1.578822 | 0.658849 | 1.578822 | up | 1.241282 | 0.311831 | 1.241282 | up | 1.085389 | 0.118212 | 1.085389 | up | 1.241282 | 0.311831 | 1.241282 | up | 0 | 0.658849 | 0.311831 | 0.118212 | 0.311831 | IKZF2 |
| 231960_at | 1.470953 | 0.556751 | 1.470953 | up | 1.470953 | 0.556751 | 1.470953 | up | 1.560664 | 0.64216 | 1.560664 | up | 1.470953 | 0.556751 | 1.470953 | up | 0 | 0.556751 | 0.556751 | 0.64216 | 0.556751 | BRWD1 |
| 232078_at | 1.413396 | 0.499166 | 1.413396 | up | 1.327204 | 0.40839 | 1.327204 | up | 1.208095 | 0.272734 | 1.208095 | up | 1.515855 | 0.600132 | 1.515855 | up | 0 | 0.499166 | 0.40839 | 0.272734 | 0.600132 | PVRL2 |
| 232113_at | 1.745618 | 0.803738 | 1.745618 | up | -1.02246 | -0.03204 | 1.022455 | down | 1.452959 | 0.538994 | 1.452959 | up | -1.1303 | -0.1767 | 1.130295 | down | 0 | 0.803738 | -0.03204 | 0.538994 | -0.1767 |  |
| 232129_s_at | 1.228054 | 0.296374 | 1.228054 | up | 1.319968 | 0.400503 | 1.319968 | up | 1.329523 | 0.410909 | 1.329523 | up | 1.674902 | 0.744076 | 1.674902 | up | 0 | 0.296374 | 0.400503 | 0.410909 | 0.744076 | LZTS2 |
| 232184_at | -1.38519 | -0.47008 | 1.385189 | down | -1.0441 | -0.06226 | 1.0441 | down | -1.02096 | -0.02993 | 1.020961 | down | -1.63014 | -0.705 | 1.630143 | down | 0 | -0.47008 | -0.06226 | -0.02993 | -0.705 | ALS2 |
| 232197_x_at | -1.016 | -0.02291 | 1.016004 | down | 1.226716 | 0.294802 | 1.226716 | up | 1.520229 | 0.604289 | 1.520229 | up | 1.110548 | 0.151271 | 1.110548 | up | 0 | -0.02291 | 0.294802 | 0.604289 | 0.151271 | ARSB |
| 232201_at | 1.231071 | 0.299914 | 1.231071 | up | 1.44486 | 0.53093 | 1.44486 | up | 1.483057 | 0.568574 | 1.483057 | up | 1.707655 | 0.772016 | 1.707655 | up | 0 | 0.299914 | 0.53093 | 0.568574 | 0.772016 | NKD2 |
| 232252_at | -1.35889 | -0.44242 | 1.358886 | down | -1.35889 | -0.44242 | 1.358886 | down | -1.35889 | -0.44242 | 1.358886 | down | -1.64855 | -0.72119 | 1.648547 | down | 0 | -0.44242 | -0.44242 | -0.44242 | -0.72119 | DUSP27 |
| 232328_at | -1.17055 | -0.22719 | 1.170553 | down | -1.65999 | -0.73118 | 1.659994 | down | -1.25278 | -0.32513 | 1.252781 | down | -1.17055 | -0.22719 | 1.170553 | down | 0 | -0.22719 | -0.73118 | -0.32513 | -0.22719 | ZNF552 |
| 232338_at | 1.679573 | 0.748095 | 1.679573 | up | 1.253224 | 0.325644 | 1.253224 | up | 1.430597 | 0.516617 | 1.430597 | up | -1.10317 | -0.14166 | 1.103174 | down | 0 | 0.748095 | 0.325644 | 0.516617 | -0.14166 | ZNF431 |
| 232412_at | 1.20383 | 0.267631 | 1.20383 | up | 1.216595 | 0.282848 | 1.216595 | up | 1.533321 | 0.61666 | 1.533321 | up | 1.182722 | 0.242111 | 1.182722 | up | 0 | 0.267631 | 0.282848 | 0.61666 | 0.242111 | FBXL20 |
| 232458_at | 1.858842 | 0.894404 | 1.858842 | up | 1.182031 | 0.241268 | 1.182031 | up | 1.201033 | 0.264276 | 1.201033 | up | 1.015001 | 0.021481 | 1.015001 | up | 0 | 0.894404 | 0.241268 | 0.264276 | 0.021481 | COL3A1 |
| 232481_s_at | 1.336595 | 0.418562 | 1.336595 | up | 1.816334 | 0.861029 | 1.816334 | up | 1.294403 | 0.372287 | 1.294403 | up | 1.184761 | 0.244596 | 1.184761 | up | 0 | 0.418562 | 0.861029 | 0.372287 | 0.244596 | SLITRK6 |
| 232574_at | -1.54467 | -0.6273 | 1.544666 | down | -1.13677 | -0.18494 | 1.136773 | down | -1.0624 | -0.08732 | 1.062397 | down | 1.03318 | 0.047092 | 1.03318 | up | 0 | -0.6273 | -0.18494 | -0.08732 | 0.047092 | XYLT1 |
| 232755_at | 1.066315 | 0.092634 | 1.066315 | up | -1.16033 | -0.21454 | 1.160332 | down | -1.11856 | -0.16164 | 1.118557 | down | -1.51967 | -0.60376 | 1.519667 | down | 0 | 0.092634 | -0.21454 | -0.16164 | -0.60376 | UBE2G2 |
| 232772_at | 1.139562 | 0.188479 | 1.139562 | up | 1.139562 | 0.188479 | 1.139562 | up | 1.139562 | 0.188479 | 1.139562 | up | 1.507302 | 0.591969 | 1.507302 | up | 0 | 0.188479 | 0.188479 | 0.188479 | 0.591969 | LOC221272 |
| 232816_s_at | 1.192209 | 0.253638 | 1.192209 | up | 1.249194 | 0.320997 | 1.249194 | up | 1.070644 | 0.098479 | 1.070644 | up | 1.505095 | 0.589854 | 1.505095 | up | 0 | 0.253638 | 0.320997 | 0.098479 | 0.589854 | DDX11 |
| 232963_at | 1.721624 | 0.78377 | 1.721624 | up | 1.0444 | 0.062674 | 1.0444 | up | -1.00118 | -0.00169 | 1.001175 | down | 1.06258 | 0.087571 | 1.06258 | up | 0 | 0.78377 | 0.062674 | -0.00169 | 0.087571 |  |
| 232988_at | -1.36869 | -0.45279 | 1.368687 | down | -1.1373 | -0.18561 | 1.137299 | down | -1.52105 | -0.60506 | 1.521047 | down | 1.122734 | 0.167017 | 1.122734 | up | 0 | -0.45279 | -0.18561 | -0.60506 | 0.167017 | GSE1 |
| 233121_at | 1.111762 | 0.152848 | 1.111762 | up | 1.553967 | 0.635956 | 1.553967 | up | 1.251962 | 0.324191 | 1.251962 | up | -1.01868 | -0.0267 | 1.018677 | down | 0 | 0.152848 | 0.635956 | 0.324191 | -0.0267 |  |
| 233303_at | 1.515117 | 0.599429 | 1.515117 | up | -1.03714 | -0.05261 | 1.037139 | down | 1.200578 | 0.263729 | 1.200578 | up | 1.136783 | 0.184957 | 1.136783 | up | 0 | 0.599429 | -0.05261 | 0.263729 | 0.184957 |  |
| 233364_s_at | 1.595498 | 0.674006 | 1.595498 | up | -1.14432 | -0.19449 | 1.144318 | down | 1.526237 | 0.609979 | 1.526237 | up | -1.24787 | -0.31947 | 1.247872 | down | 0 | 0.674006 | -0.19449 | 0.609979 | -0.31947 |  |
| 233402_at | 1.24278 | 0.313571 | 1.24278 | up | -1.10066 | -0.13837 | 1.10066 | down | -1.50812 | -0.59275 | 1.508123 | down | 1.193546 | 0.255254 | 1.193546 | up | 0 | 0.313571 | -0.13837 | -0.59275 | 0.255254 |  |
| 233476_at | -1.53342 | -0.61676 | 1.533422 | down | 1.046124 | 0.065053 | 1.046124 | up | -1.13556 | -0.18341 | 1.135562 | down | -1.13556 | -0.18341 | 1.135562 | down | 0 | -0.61676 | 0.065053 | -0.18341 | -0.18341 |  |
| 233507_at | -1.39997 | -0.48539 | 1.399968 | down | -1.39997 | -0.48539 | 1.399968 | down | -1.69171 | -0.75848 | 1.691708 | down | -1.41941 | -0.50529 | 1.419407 | down | 0 | -0.48539 | -0.48539 | -0.75848 | -0.50529 |  |
| 233518_at | 1.536641 | 0.61978 | 1.536641 | up | 1.084637 | 0.117212 | 1.084637 | up | 1.429873 | 0.515887 | 1.429873 | up | 1.126732 | 0.172144 | 1.126732 | up | 0 | 0.61978 | 0.117212 | 0.515887 | 0.172144 |  |
| 233524_at | -1.62373 | -0.69931 | 1.623733 | down | -1.10282 | -0.14119 | 1.102817 | down | -1.06491 | -0.09073 | 1.064911 | down | 1.005557 | 0.007995 | 1.005557 | up | 0 | -0.69931 | -0.14119 | -0.09073 | 0.007995 | LINC00475///LINC00475 |
| 233544_at | -1.14136 | -0.19076 | 1.141365 | down | 1.501322 | 0.586234 | 1.501322 | up | -1.01313 | -0.01881 | 1.013125 | down | -1 | 0 | 1 | down | 0 | -0.19076 | 0.586234 | -0.01881 | 0 | GNL1 |
| 233708_at | 1.11405 | 0.155814 | 1.11405 | up | 1.11405 | 0.155814 | 1.11405 | up | 1.11405 | 0.155814 | 1.11405 | up | -1.52503 | -0.60883 | 1.525026 | down | 0 | 0.155814 | 0.155814 | 0.155814 | -0.60883 | NFATC2 |
| 233723_at | 1.474171 | 0.559904 | 1.474171 | up | 1.297273 | 0.375483 | 1.297273 | up | 1.510527 | 0.595052 | 1.510527 | up | 1.167674 | 0.223638 | 1.167674 | up | 0 | 0.559904 | 0.375483 | 0.595052 | 0.223638 |  |
| 233895_at | 1.351547 | 0.434611 | 1.351547 | up | 1.454169 | 0.540195 | 1.454169 | up | 1.233929 | 0.303259 | 1.233929 | up | 1.526965 | 0.610667 | 1.526965 | up | 0 | 0.434611 | 0.540195 | 0.303259 | 0.610667 | ANKRD24 |
| 233928_at | -1.52665 | -0.61037 | 1.526648 | down | -1.19746 | -0.25998 | 1.197464 | down | -1.20472 | -0.2687 | 1.204724 | down | -1.19746 | -0.25998 | 1.197464 | down | 0 | -0.61037 | -0.25998 | -0.2687 | -0.25998 | ADAMTS9-AS1 |
| 234001_s_at | 1.203037 | 0.266681 | 1.203037 | up | 1.280059 | 0.35621 | 1.280059 | up | 1.280059 | 0.35621 | 1.280059 | up | 1.597735 | 0.676028 | 1.597735 | up | 0 | 0.266681 | 0.35621 | 0.35621 | 0.676028 | ARFGAP1 |
| 234089_at | -1.3455 | -0.42814 | 1.345496 | down | -1.67883 | -0.74745 | 1.678827 | down | -1.3455 | -0.42814 | 1.345496 | down | -1.3455 | -0.42814 | 1.345496 | down | 0 | -0.42814 | -0.74745 | -0.42814 | -0.42814 |  |
| 234106_s_at | 1.08423 | 0.11667 | 1.08423 | up | 1.754372 | 0.810955 | 1.754372 | up | 1.468307 | 0.554153 | 1.468307 | up | 1.813784 | 0.859003 | 1.813784 | up | 0 | 0.11667 | 0.810955 | 0.554153 | 0.859003 | FLYWCH1 |
| 234153_at | -1.45482 | -0.54084 | 1.454816 | down | -1.36219 | -0.44592 | 1.362187 | down | -1.52742 | -0.6111 | 1.527422 | down | -1.36014 | -0.44376 | 1.360143 | down | 0 | -0.54084 | -0.44592 | -0.6111 | -0.44376 |  |
| 234171_at | -1.1808 | -0.23976 | 1.180796 | down | -1.43045 | -0.51647 | 1.430454 | down | -1.18768 | -0.24815 | 1.18768 | down | -1.55227 | -0.63438 | 1.552272 | down | 0 | -0.23976 | -0.51647 | -0.24815 | -0.63438 | LINC01267 |
| 234231_at | -1.53373 | -0.61705 | 1.533732 | down | -1.11518 | -0.15727 | 1.115176 | down | -1.09552 | -0.13162 | 1.095522 | down | -1.11518 | -0.15727 | 1.115176 | down | 0 | -0.61705 | -0.15727 | -0.13162 | -0.15727 | CASP16P |
| 234295_at | -1.30577 | -0.3849 | 1.305773 | down | -1.02003 | -0.02861 | 1.02003 | down | -1.02804 | -0.03989 | 1.028035 | down | -1.51761 | -0.6018 | 1.517611 | down | 0 | -0.3849 | -0.02861 | -0.03989 | -0.6018 | DBR1 |
| 234422_at | -1.02235 | -0.03189 | 1.022348 | down | -1.30757 | -0.38689 | 1.307568 | down | -1.71108 | -0.77491 | 1.71108 | down | -1.07435 | -0.10346 | 1.074349 | down | 0 | -0.03189 | -0.38689 | -0.77491 | -0.10346 |  |
| 234542_at | -1.55255 | -0.63464 | 1.55255 | down | -1.36722 | -0.45125 | 1.367221 | down | -1.31046 | -0.39007 | 1.310455 | down | -1.31046 | -0.39007 | 1.310455 | down | 0 | -0.63464 | -0.45125 | -0.39007 | -0.39007 | ARHGEF12 |
| 234574_at | -1.71258 | -0.77617 | 1.71258 | down | -1.27985 | -0.35597 | 1.279845 | down | -1.28208 | -0.35849 | 1.282082 | down | -1.27985 | -0.35597 | 1.279845 | down | 0 | -0.77617 | -0.35597 | -0.35849 | -0.35597 |  |
| 234657_at | -1.30151 | -0.38018 | 1.301507 | down | -1.50733 | -0.592 | 1.507334 | down | -1.32153 | -0.4022 | 1.321525 | down | -1.38722 | -0.4722 | 1.387224 | down | 0 | -0.38018 | -0.592 | -0.4022 | -0.4722 | FAM126A |
| 234753_x_at | -1.31457 | -0.39459 | 1.314566 | down | -1.57749 | -0.65763 | 1.57749 | down | -1.30036 | -0.37891 | 1.300361 | down | -1.1368 | -0.18498 | 1.136805 | down | 0 | -0.39459 | -0.65763 | -0.37891 | -0.18498 |  |
| 234914_at | 1.268878 | 0.343553 | 1.268878 | up | 1.19524 | 0.257301 | 1.19524 | up | 1.268878 | 0.343553 | 1.268878 | up | 1.525359 | 0.609149 | 1.525359 | up | 0 | 0.343553 | 0.257301 | 0.343553 | 0.609149 | ZNF7 |
| 234996_at | -1.27032 | -0.34519 | 1.27032 | down | -1.29288 | -0.37059 | 1.292885 | down | -1.7216 | -0.78375 | 1.7216 | down | -1.66357 | -0.73428 | 1.663573 | down | 0 | -0.34519 | -0.37059 | -0.78375 | -0.73428 | CALCRL |
| 235177_at | -1.13405 | -0.18148 | 1.134049 | down | -1.08281 | -0.11478 | 1.082807 | down | -1.11448 | -0.15637 | 1.114478 | down | -1.53423 | -0.61752 | 1.534231 | down | 0 | -0.18148 | -0.11478 | -0.15637 | -0.61752 | METTL21A |
| 235210_s_at | 1.62419 | 0.699721 | 1.62419 | up | -1 | 0 | 1 | down | 1.168775 | 0.224997 | 1.168775 | up | -1.00662 | -0.00952 | 1.006622 | down | 0 | 0.699721 | 0 | 0.224997 | -0.00952 | SBSPON |
| 235267_at | 1.06391 | 0.089376 | 1.06391 | up | 1.21934 | 0.286101 | 1.21934 | up | 1.136091 | 0.184079 | 1.136091 | up | -1.50216 | -0.58704 | 1.502157 | down | 0 | 0.089376 | 0.286101 | 0.184079 | -0.58704 | MAGI2-AS3 |
| 235293_at | 1.270507 | 0.345405 | 1.270507 | up | 1.211937 | 0.277315 | 1.211937 | up | 1.211937 | 0.277315 | 1.211937 | up | 1.527264 | 0.610949 | 1.527264 | up | 0 | 0.345405 | 0.277315 | 0.277315 | 0.610949 |  |
| 235393_at | 1.258947 | 0.332218 | 1.258947 | up | -1.60322 | -0.68097 | 1.603216 | down | 1.038244 | 0.054145 | 1.038244 | up | -1 | 0 | 1 | down | 0 | 0.332218 | -0.68097 | 0.054145 | 0 |  |
| 235590_at | 1.039652 | 0.0561 | 1.039652 | up | 1.039652 | 0.0561 | 1.039652 | up | 1.159269 | 0.213215 | 1.159269 | up | -1.51628 | -0.60054 | 1.516281 | down | 0 | 0.0561 | 0.0561 | 0.213215 | -0.60054 | SLF2 |
| 235720_at | 1.415846 | 0.501664 | 1.415846 | up | 1.392504 | 0.477682 | 1.392504 | up | 1.710653 | 0.774547 | 1.710653 | up | 1.392504 | 0.477682 | 1.392504 | up | 0 | 0.501664 | 0.477682 | 0.774547 | 0.477682 | CRIP3 |
| 235787_at | 1.133588 | 0.180896 | 1.133588 | up | -1 | 0 | 1 | down | 1.093305 | 0.128696 | 1.093305 | up | -1.53096 | -0.61444 | 1.530965 | down | 0 | 0.180896 | 0 | 0.128696 | -0.61444 | CDC37L1 |
| 235807_at | 1.368162 | 0.452239 | 1.368162 | up | 1.551324 | 0.6335 | 1.551324 | up | 1.501634 | 0.586534 | 1.501634 | up | 1.321089 | 0.401727 | 1.321089 | up | 0 | 0.452239 | 0.6335 | 0.586534 | 0.401727 |  |
| 235859_at | -1.59224 | -0.67106 | 1.592241 | down | -1.18576 | -0.24581 | 1.185757 | down | -1.50284 | -0.58769 | 1.502841 | down | -1.36873 | -0.45284 | 1.368731 | down | 0 | -0.67106 | -0.24581 | -0.58769 | -0.45284 | KMT2C |
| 236023_at | 1.12568 | 0.170797 | 1.12568 | up | 1.970239 | 0.97837 | 1.970239 | up | 1.68578 | 0.753416 | 1.68578 | up | 1.232011 | 0.301015 | 1.232011 | up | 0 | 0.170797 | 0.97837 | 0.753416 | 0.301015 | CDK9 |
| 236302_at | 1.182092 | 0.241342 | 1.182092 | up | 1.543483 | 0.62619 | 1.543483 | up | 1.206549 | 0.270886 | 1.206549 | up | 1.328865 | 0.410194 | 1.328865 | up | 0 | 0.241342 | 0.62619 | 0.270886 | 0.410194 | PPM1E |
| 236333_at | 1.064875 | 0.090683 | 1.064875 | up | 1.225995 | 0.293953 | 1.225995 | up | 1.225995 | 0.293953 | 1.225995 | up | 1.56352 | 0.644798 | 1.56352 | up | 0 | 0.090683 | 0.293953 | 0.293953 | 0.644798 |  |
| 236503_at | 1.022268 | 0.031774 | 1.022268 | up | -1.03722 | -0.05273 | 1.037224 | down | 1.00758 | 0.010895 | 1.00758 | up | -1.50898 | -0.59357 | 1.50898 | down | 0 | 0.031774 | -0.05273 | 0.010895 | -0.59357 |  |
| 236552_at | 1.901778 | 0.927349 | 1.901778 | up | -1.06192 | -0.08667 | 1.06192 | down | -1 | 0 | 1 | down | -1.06215 | -0.08699 | 1.062154 | down | 0 | 0.927349 | -0.08667 | 0 | -0.08699 |  |
| 236560_at | 1.644003 | 0.717213 | 1.644003 | up | 1.183821 | 0.243451 | 1.183821 | up | 1.357825 | 0.441298 | 1.357825 | up | 1.357825 | 0.441298 | 1.357825 | up | 0 | 0.717213 | 0.243451 | 0.441298 | 0.441298 | DIS3 |
| 236598_at | 1.022555 | 0.032178 | 1.022555 | up | 1.520598 | 0.604638 | 1.520598 | up | 1.270218 | 0.345076 | 1.270218 | up | 1.344359 | 0.426919 | 1.344359 | up | 0 | 0.032178 | 0.604638 | 0.345076 | 0.426919 | LOC100996579 |
| 236606_at | 1.029275 | 0.041628 | 1.029275 | up | 1.009315 | 0.013376 | 1.009315 | up | 1.009315 | 0.013376 | 1.009315 | up | -1.56474 | -0.64593 | 1.564742 | down | 0 | 0.041628 | 0.013376 | 0.013376 | -0.64593 |  |
| 236652_at | 1.235681 | 0.305306 | 1.235681 | up | 1.615999 | 0.692426 | 1.615999 | up | 1.235681 | 0.305306 | 1.235681 | up | 1.197965 | 0.260586 | 1.197965 | up | 0 | 0.305306 | 0.692426 | 0.305306 | 0.260586 | LOC149703 |
| 236672_at | -1.3351 | -0.41694 | 1.335096 | down | -1.29309 | -0.37082 | 1.293088 | down | -1.57343 | -0.65391 | 1.573426 | down | -1.25123 | -0.32334 | 1.251228 | down | 0 | -0.41694 | -0.37082 | -0.65391 | -0.32334 | ZNF681 |
| 236808_at | 1.563496 | 0.644775 | 1.563496 | up | 1.075614 | 0.10516 | 1.075614 | up | 1.328505 | 0.409803 | 1.328505 | up | 1.08982 | 0.124089 | 1.08982 | up | 0 | 0.644775 | 0.10516 | 0.409803 | 0.124089 | FGFR1OP2 |
| 236841_at | 1.143697 | 0.193705 | 1.143697 | up | 1.143697 | 0.193705 | 1.143697 | up | 1.589516 | 0.668588 | 1.589516 | up | 1.143697 | 0.193705 | 1.143697 | up | 0 | 0.193705 | 0.193705 | 0.668588 | 0.193705 | WASH1 |
| 236931_at | 1.198432 | 0.261148 | 1.198432 | up | 1.198432 | 0.261148 | 1.198432 | up | 1.503232 | 0.588068 | 1.503232 | up | 1.143775 | 0.193803 | 1.143775 | up | 0 | 0.261148 | 0.261148 | 0.588068 | 0.193803 |  |
| 236937_at | -1.30103 | -0.37965 | 1.301028 | down | -1.29631 | -0.37442 | 1.296315 | down | -1.30103 | -0.37965 | 1.301028 | down | -1.57936 | -0.65934 | 1.579364 | down | 0 | -0.37965 | -0.37442 | -0.37965 | -0.65934 | VPS8 |
| 237073_at | -1.27019 | -0.34505 | 1.270194 | down | -1.50932 | -0.5939 | 1.50932 | down | -1.52253 | -0.60647 | 1.522526 | down | -1.09271 | -0.12791 | 1.092711 | down | 0 | -0.34505 | -0.5939 | -0.60647 | -0.12791 |  |
| 237090_at | 1.236087 | 0.305781 | 1.236087 | up | 1.236087 | 0.305781 | 1.236087 | up | 1.236087 | 0.305781 | 1.236087 | up | 1.535611 | 0.618813 | 1.535611 | up | 0 | 0.305781 | 0.305781 | 0.305781 | 0.618813 | LOC100506113 |
| 237218_at | 1.633175 | 0.70768 | 1.633175 | up | -1 | 0 | 1 | down | -1 | 0 | 1 | down | -1.1288 | -0.17479 | 1.128803 | down | 0 | 0.70768 | 0 | 0 | -0.17479 |  |
| 237571_at | 1.032867 | 0.046654 | 1.032867 | up | -1.17385 | -0.23125 | 1.173853 | down | -1.15026 | -0.20196 | 1.150264 | down | -1.52042 | -0.60447 | 1.520419 | down | 0 | 0.046654 | -0.23125 | -0.20196 | -0.60447 |  |
| 237764_at | -1.29095 | -0.36843 | 1.290951 | down | -1.28676 | -0.36374 | 1.28676 | down | -1.54381 | -0.6265 | 1.54381 | down | -1.28676 | -0.36374 | 1.28676 | down | 0 | -0.36843 | -0.36374 | -0.6265 | -0.36374 | LOC101928111 |
| 237792_at | -1.13051 | -0.17698 | 1.130512 | down | -1.13051 | -0.17698 | 1.130512 | down | -1.50721 | -0.59188 | 1.507213 | down | -1.13051 | -0.17698 | 1.130512 | down | 0 | -0.17698 | -0.17698 | -0.59188 | -0.17698 |  |
| 237807_at | -1.39686 | -0.48219 | 1.396858 | down | -1.42209 | -0.50801 | 1.422085 | down | -1.53766 | -0.62074 | 1.537659 | down | -1.39686 | -0.48219 | 1.396858 | down | 0 | -0.48219 | -0.50801 | -0.62074 | -0.48219 | SPATA12 |
| 237914_s_at | 1.083123 | 0.115198 | 1.083123 | up | 1.187134 | 0.247483 | 1.187134 | up | 1.187134 | 0.247483 | 1.187134 | up | 1.506883 | 0.591568 | 1.506883 | up | 0 | 0.115198 | 0.247483 | 0.247483 | 0.591568 |  |
| 238199_x_at | 1.385968 | 0.470894 | 1.385968 | up | 1.280537 | 0.356749 | 1.280537 | up | 1.570368 | 0.651103 | 1.570368 | up | -1.1281 | -0.1739 | 1.128102 | down | 0 | 0.470894 | 0.356749 | 0.651103 | -0.1739 | COX3 |
| 238369_s_at | -1.49675 | -0.58183 | 1.49675 | down | -1.50576 | -0.59049 | 1.505757 | down | -1.20774 | -0.27231 | 1.207741 | down | -1.20774 | -0.27231 | 1.207741 | down | 0 | -0.58183 | -0.59049 | -0.27231 | -0.27231 | LINC01607 |
| 238458_at | -1.16428 | -0.21944 | 1.164283 | down | 1.02602 | 0.037059 | 1.02602 | up | -1.03065 | -0.04356 | 1.030654 | down | -1.53598 | -0.61916 | 1.535976 | down | 0 | -0.21944 | 0.037059 | -0.04356 | -0.61916 | MICU3 |
| 238459_x_at | 1.412839 | 0.498597 | 1.412839 | up | 1.70619 | 0.770778 | 1.70619 | up | 1.397308 | 0.48265 | 1.397308 | up | -1.24642 | -0.31779 | 1.246416 | down | 0 | 0.498597 | 0.770778 | 0.48265 | -0.31779 | SPATA6 |
| 238468_at | 1.081081 | 0.112475 | 1.081081 | up | 1.066767 | 0.093246 | 1.066767 | up | 1.081081 | 0.112475 | 1.081081 | up | 1.659078 | 0.730382 | 1.659078 | up | 0 | 0.112475 | 0.093246 | 0.112475 | 0.730382 | TNRC6B |
| 238476_at | 1.18895 | 0.249689 | 1.18895 | up | 1.236631 | 0.306415 | 1.236631 | up | 1.609256 | 0.686394 | 1.609256 | up | 1.101107 | 0.138954 | 1.101107 | up | 0 | 0.249689 | 0.306415 | 0.686394 | 0.138954 | CREBRF |
| 238501_at | -1.00225 | -0.00324 | 1.002251 | down | 1.073007 | 0.101659 | 1.073007 | up | -1.00225 | -0.00324 | 1.002251 | down | -1.51952 | -0.60361 | 1.519518 | down | 0 | -0.00324 | 0.101659 | -0.00324 | -0.60361 |  |
| 238522_at | 1.183856 | 0.243494 | 1.183856 | up | 1.544272 | 0.626927 | 1.544272 | up | 1.592986 | 0.671733 | 1.592986 | up | 1.038629 | 0.05468 | 1.038629 | up | 0 | 0.243494 | 0.626927 | 0.671733 | 0.05468 | PPP3CB-AS1 |
| 238532_at | -1.12689 | -0.17234 | 1.126886 | down | -1.17035 | -0.22693 | 1.170345 | down | -1.14665 | -0.19743 | 1.146651 | down | -1.60305 | -0.68082 | 1.603047 | down | 0 | -0.17234 | -0.22693 | -0.19743 | -0.68082 | DPF3 |
| 238594_x_at | -1.04073 | -0.0576 | 1.040732 | down | -1.53443 | -0.6177 | 1.534427 | down | -1.49681 | -0.58189 | 1.49681 | down | 1.062681 | 0.087709 | 1.062681 | up | 0 | -0.0576 | -0.6177 | -0.58189 | 0.087709 | DUSP8 |
| 238610_s_at | 1.581975 | 0.661727 | 1.581975 | up | 1.15598 | 0.209117 | 1.15598 | up | 1.127907 | 0.173648 | 1.127907 | up | 1.127907 | 0.173648 | 1.127907 | up | 0 | 0.661727 | 0.209117 | 0.173648 | 0.173648 |  |
| 238692_at | -1.25291 | -0.32529 | 1.252914 | down | -1.17895 | -0.23751 | 1.178953 | down | -1.23083 | -0.29963 | 1.230831 | down | -1.50028 | -0.58523 | 1.500278 | down | 0 | -0.32529 | -0.23751 | -0.29963 | -0.58523 | BTBD11 |
| 238743_at | 1.167062 | 0.222881 | 1.167062 | up | 1.302082 | 0.38082 | 1.302082 | up | 1.302082 | 0.38082 | 1.302082 | up | 1.567171 | 0.648163 | 1.567171 | up | 0 | 0.222881 | 0.38082 | 0.38082 | 0.648163 |  |
| 238811_at | 1.36838 | 0.452468 | 1.36838 | up | 1.36838 | 0.452468 | 1.36838 | up | 1.536749 | 0.619882 | 1.536749 | up | 1.296194 | 0.374282 | 1.296194 | up | 0 | 0.452468 | 0.452468 | 0.619882 | 0.374282 | ATP11B |
| 238852_at | -1.209 | -0.27382 | 1.209003 | down | 1.053159 | 0.074724 | 1.053159 | up | -1.10986 | -0.15038 | 1.109861 | down | -1.51038 | -0.59491 | 1.510383 | down | 0 | -0.27382 | 0.074724 | -0.15038 | -0.59491 | PRRX1 |
| 238866_at | 1.681547 | 0.749789 | 1.681547 | up | 1.020143 | 0.028771 | 1.020143 | up | 1.35553 | 0.438857 | 1.35553 | up | 1.345168 | 0.427786 | 1.345168 | up | 0 | 0.749789 | 0.028771 | 0.438857 | 0.427786 | C19orf68 |
| 238953_at | 1.531666 | 0.615102 | 1.531666 | up | -1.08343 | -0.1156 | 1.083427 | down | -1.00488 | -0.00703 | 1.004884 | down | -1.00488 | -0.00703 | 1.004884 | down | 0 | 0.615102 | -0.1156 | -0.00703 | -0.00703 | SP2-AS1 |
| 238982_at | 1.560085 | 0.641624 | 1.560085 | up | 1.133662 | 0.18099 | 1.133662 | up | 1.112819 | 0.154219 | 1.112819 | up | 1.235614 | 0.305228 | 1.235614 | up | 0 | 0.641624 | 0.18099 | 0.154219 | 0.305228 | DENR |
| 239046_at | 1.591965 | 0.670808 | 1.591965 | up | 1.375977 | 0.460456 | 1.375977 | up | 1.456542 | 0.542548 | 1.456542 | up | 1.456542 | 0.542548 | 1.456542 | up | 0 | 0.670808 | 0.460456 | 0.542548 | 0.542548 |  |
| 239156_at | -1.67776 | -0.74654 | 1.677762 | down | 1.076171 | 0.105907 | 1.076171 | up | -1 | 0 | 1 | down | -1 | 0 | 1 | down | 0 | -0.74654 | 0.105907 | 0 | 0 |  |
| 239220_at | -1.10026 | -0.13785 | 1.100263 | down | -1.28035 | -0.35653 | 1.280346 | down | -1.67079 | -0.74053 | 1.670791 | down | -1.16902 | -0.2253 | 1.169017 | down | 0 | -0.13785 | -0.35653 | -0.74053 | -0.2253 |  |
| 239232_at | -1.51437 | -0.59872 | 1.514374 | down | -1.17592 | -0.23379 | 1.17592 | down | -1.16994 | -0.22644 | 1.169941 | down | -1.17592 | -0.23379 | 1.17592 | down | 0 | -0.59872 | -0.23379 | -0.22644 | -0.23379 | MSI2 |
| 239295_at | 1.868047 | 0.901531 | 1.868047 | up | -1 | 0 | 1 | down | -1 | 0 | 1 | down | 1.076453 | 0.106286 | 1.076453 | up | 0 | 0.901531 | 0 | 0 | 0.106286 | SRSF12 |
| 239331_at | 1.625434 | 0.700825 | 1.625434 | up | 1.086012 | 0.11904 | 1.086012 | up | 1.39786 | 0.48322 | 1.39786 | up | 1.179238 | 0.237854 | 1.179238 | up | 0 | 0.700825 | 0.11904 | 0.48322 | 0.237854 |  |
| 239383_at | 1.139209 | 0.188033 | 1.139209 | up | -1.52564 | -0.60942 | 1.525642 | down | -1 | 0 | 1 | down | 1.094739 | 0.130588 | 1.094739 | up | 0 | 0.188033 | -0.60942 | 0 | 0.130588 |  |
| 239450_at | 1.232019 | 0.301025 | 1.232019 | up | 1.232019 | 0.301025 | 1.232019 | up | 1.511157 | 0.595653 | 1.511157 | up | 1.560341 | 0.641861 | 1.560341 | up | 0 | 0.301025 | 0.301025 | 0.595653 | 0.641861 | NDUFV2-AS1 |
| 239458_at | -1.19788 | -0.26049 | 1.197883 | down | 1.006522 | 0.009379 | 1.006522 | up | 1.052612 | 0.073973 | 1.052612 | up | -1.51035 | -0.59488 | 1.510345 | down | 0 | -0.26049 | 0.009379 | 0.073973 | -0.59488 | ALS2CR12 |
| 239465_at | 1.570435 | 0.651164 | 1.570435 | up | 1.168407 | 0.224543 | 1.168407 | up | 1.168407 | 0.224543 | 1.168407 | up | 1.168407 | 0.224543 | 1.168407 | up | 0 | 0.651164 | 0.224543 | 0.224543 | 0.224543 |  |
| 239485_at | -1.50135 | -0.58626 | 1.501354 | down | -1.24022 | -0.3106 | 1.240224 | down | -1.09459 | -0.13039 | 1.09459 | down | -1.17565 | -0.23346 | 1.175651 | down | 0 | -0.58626 | -0.3106 | -0.13039 | -0.23346 | CDH4 |
| 239497_at | -1.19333 | -0.25499 | 1.193327 | down | -1.28109 | -0.35737 | 1.281087 | down | -1.53939 | -0.62236 | 1.539388 | down | 1.005012 | 0.007212 | 1.005012 | up | 0 | -0.25499 | -0.35737 | -0.62236 | 0.007212 |  |
| 239579_at | -1 | 0 | 1 | down | 1.098069 | 0.134968 | 1.098069 | up | -1.08546 | -0.11831 | 1.085462 | down | -1.65725 | -0.72879 | 1.657249 | down | 0 | 0 | 0.134968 | -0.11831 | -0.72879 | EPHX4 |
| 239736_at | -1.14396 | -0.19403 | 1.143956 | down | -1.08496 | -0.11764 | 1.084962 | down | 1.539444 | 0.622409 | 1.539444 | up | 1.075261 | 0.104686 | 1.075261 | up | 0 | -0.19403 | -0.11764 | 0.622409 | 0.104686 | LINC01214 |
| 240003_at | 1.23523 | 0.304779 | 1.23523 | up | 1.065523 | 0.091562 | 1.065523 | up | 1.015599 | 0.02233 | 1.015599 | up | 1.500916 | 0.585843 | 1.500916 | up | 0 | 0.304779 | 0.091562 | 0.02233 | 0.585843 |  |
| 240199_x_at | 1.709877 | 0.773892 | 1.709877 | up | 1.18181 | 0.240998 | 1.18181 | up | 1.18181 | 0.240998 | 1.18181 | up | 1.18181 | 0.240998 | 1.18181 | up | 0 | 0.773892 | 0.240998 | 0.240998 | 0.240998 | ZNF345 |
| 240256_at | 1.502122 | 0.587002 | 1.502122 | up | -1 | 0 | 1 | down | -1.12299 | -0.16734 | 1.122986 | down | -1 | 0 | 1 | down | 0 | 0.587002 | 0 | -0.16734 | 0 |  |
| 240300_at | -1.37666 | -0.46117 | 1.376655 | down | -1.27766 | -0.3535 | 1.277661 | down | -1.27766 | -0.3535 | 1.277661 | down | -1.57359 | -0.65406 | 1.573592 | down | 0 | -0.46117 | -0.3535 | -0.3535 | -0.65406 | TK2 |
| 240528_s_at | 1.274872 | 0.350352 | 1.274872 | up | 1.752029 | 0.809026 | 1.752029 | up | 1.569783 | 0.650565 | 1.569783 | up | 1.390883 | 0.476001 | 1.390883 | up | 0 | 0.350352 | 0.809026 | 0.650565 | 0.476001 | EXOC4 |
| 240529_at | -1.47452 | -0.56025 | 1.474521 | down | -1.47452 | -0.56025 | 1.474521 | down | -1.47452 | -0.56025 | 1.474521 | down | -1.66617 | -0.73654 | 1.666169 | down | 0 | -0.56025 | -0.56025 | -0.56025 | -0.73654 |  |
| 240555_at | -1.20088 | -0.26409 | 1.200875 | down | -1.20088 | -0.26409 | 1.200875 | down | -1.50922 | -0.59381 | 1.509223 | down | -1.20088 | -0.26409 | 1.200875 | down | 0 | -0.26409 | -0.26409 | -0.59381 | -0.26409 |  |
| 240594_at | 1.521909 | 0.605882 | 1.521909 | up | 1.069503 | 0.096941 | 1.069503 | up | 1.159441 | 0.213429 | 1.159441 | up | 1.143573 | 0.193548 | 1.143573 | up | 0 | 0.605882 | 0.096941 | 0.213429 | 0.193548 |  |
| 240909_at | -1.53701 | -0.62013 | 1.537013 | down | -1.38909 | -0.47414 | 1.389086 | down | -1.26627 | -0.34058 | 1.266266 | down | -1.19643 | -0.25874 | 1.196431 | down | 0 | -0.62013 | -0.47414 | -0.34058 | -0.25874 |  |
| 241288_at | -1.20674 | -0.27111 | 1.206737 | down | -1.20674 | -0.27111 | 1.206737 | down | -1.50207 | -0.58695 | 1.502073 | down | -1.22218 | -0.28946 | 1.222179 | down | 0 | -0.27111 | -0.27111 | -0.58695 | -0.28946 |  |
| 241314_at | 1.238148 | 0.308184 | 1.238148 | up | -1.0758 | -0.1054 | 1.075796 | down | 1.143684 | 0.193688 | 1.143684 | up | 1.588299 | 0.667482 | 1.588299 | up | 0 | 0.308184 | -0.1054 | 0.193688 | 0.667482 |  |
| 241448_at | 1.693474 | 0.759986 | 1.693474 | up | 1.110193 | 0.150811 | 1.110193 | up | 1.289586 | 0.366908 | 1.289586 | up | 1.359841 | 0.443438 | 1.359841 | up | 0 | 0.759986 | 0.150811 | 0.366908 | 0.443438 |  |
| 241844_x_at | -1 | 0 | 1 | down | -1 | 0 | 1 | down | 1.176136 | 0.234055 | 1.176136 | up | -1.80423 | -0.85138 | 1.804227 | down | 0 | 0 | 0 | 0.234055 | -0.85138 | TMEM156 |
| 241879_at | 1.910015 | 0.933584 | 1.910015 | up | -1.11085 | -0.15166 | 1.110847 | down | -1 | 0 | 1 | down | -1 | 0 | 1 | down | 0 | 0.933584 | -0.15166 | 0 | 0 | LPP |
| 242005_at | -1.18659 | -0.24682 | 1.186589 | down | 1.109764 | 0.150253 | 1.109764 | up | -1.11945 | -0.16279 | 1.119454 | down | -1.72629 | -0.78767 | 1.72629 | down | 0 | -0.24682 | 0.150253 | -0.16279 | -0.78767 |  |
| 242265_at | 1.502001 | 0.586886 | 1.502001 | up | 1.204609 | 0.268565 | 1.204609 | up | 1.111861 | 0.152977 | 1.111861 | up | 1.111861 | 0.152977 | 1.111861 | up | 0 | 0.586886 | 0.268565 | 0.152977 | 0.152977 | BRD8 |
| 242308_at | 1.336333 | 0.41828 | 1.336333 | up | 1.193049 | 0.254653 | 1.193049 | up | 1.569915 | 0.650687 | 1.569915 | up | 1.482379 | 0.567915 | 1.482379 | up | 0 | 0.41828 | 0.254653 | 0.650687 | 0.567915 | MCOLN3 |
| 242352_at | 1.419683 | 0.505569 | 1.419683 | up | 1.173976 | 0.231403 | 1.173976 | up | 1.533398 | 0.616733 | 1.533398 | up | -1.00776 | -0.01116 | 1.007762 | down | 0 | 0.505569 | 0.231403 | 0.616733 | -0.01116 | NIPBL |
| 242402_x_at | -1.54145 | -0.62429 | 1.541447 | down | -1.24022 | -0.3106 | 1.240224 | down | -1.14909 | -0.20049 | 1.149088 | down | -1.4921 | -0.57735 | 1.492102 | down | 0 | -0.62429 | -0.3106 | -0.20049 | -0.57735 | ARAP2 |
| 242422_at | 1.617257 | 0.693549 | 1.617257 | up | 1.304451 | 0.383442 | 1.304451 | up | 1.407302 | 0.492932 | 1.407302 | up | 1.407302 | 0.492932 | 1.407302 | up | 0 | 0.693549 | 0.383442 | 0.492932 | 0.492932 | G3BP1 |
| 242550_at | 1.248105 | 0.319739 | 1.248105 | up | 1.035222 | 0.04994 | 1.035222 | up | 1.537196 | 0.620301 | 1.537196 | up | -1.09171 | -0.12659 | 1.09171 | down | 0 | 0.319739 | 0.04994 | 0.620301 | -0.12659 | EIF3B |
| 242587_at | 1.223026 | 0.290455 | 1.223026 | up | 1.64114 | 0.714698 | 1.64114 | up | 1.382221 | 0.466988 | 1.382221 | up | 1.312247 | 0.392039 | 1.312247 | up | 0 | 0.290455 | 0.714698 | 0.466988 | 0.392039 | SLC9A9 |
| 242613_at | -1.46589 | -0.55177 | 1.465885 | down | -1.34578 | -0.42844 | 1.345778 | down | -1.35099 | -0.43402 | 1.35099 | down | -1.59244 | -0.67124 | 1.592436 | down | 0 | -0.55177 | -0.42844 | -0.43402 | -0.67124 |  |
| 242684_at | 1.461327 | 0.547279 | 1.461327 | up | 1.376265 | 0.460758 | 1.376265 | up | 1.376265 | 0.460758 | 1.376265 | up | 1.740946 | 0.799871 | 1.740946 | up | 0 | 0.547279 | 0.460758 | 0.460758 | 0.799871 | ZNF425 |
| 242691_at | 1.739315 | 0.798519 | 1.739315 | up | 1.350148 | 0.433117 | 1.350148 | up | 1.494046 | 0.579224 | 1.494046 | up | 1.229344 | 0.297888 | 1.229344 | up | 0 | 0.798519 | 0.433117 | 0.579224 | 0.297888 |  |
| 242785_at | 1.037549 | 0.05318 | 1.037549 | up | 1.037549 | 0.05318 | 1.037549 | up | 1.082273 | 0.114064 | 1.082273 | up | 1.520421 | 0.604471 | 1.520421 | up | 0 | 0.05318 | 0.05318 | 0.114064 | 0.604471 | EML6 |
| 242833_at | -1.22671 | -0.29479 | 1.226705 | down | -1.74286 | -0.80145 | 1.742857 | down | -1.22671 | -0.29479 | 1.226705 | down | -1.22671 | -0.29479 | 1.226705 | down | 0 | -0.29479 | -0.80145 | -0.29479 | -0.29479 | LOC105376230 |
| 242856_at | -1.01758 | -0.02514 | 1.017577 | down | 1.133223 | 0.180432 | 1.133223 | up | -1.01758 | -0.02514 | 1.017577 | down | -1.58828 | -0.66747 | 1.588282 | down | 0 | -0.02514 | 0.180432 | -0.02514 | -0.66747 |  |
| 242996_at | -1.07657 | -0.10644 | 1.076571 | down | -1.0166 | -0.02375 | 1.016596 | down | -1.13329 | -0.18052 | 1.133294 | down | -1.56499 | -0.64615 | 1.564987 | down | 0 | -0.10644 | -0.02375 | -0.18052 | -0.64615 | MTRF1 |
| 243000_at | 1.037194 | 0.052686 | 1.037194 | up | 1.00123 | 0.001774 | 1.00123 | up | 1.128915 | 0.174937 | 1.128915 | up | -1.63941 | -0.71318 | 1.639415 | down | 0 | 0.052686 | 0.001774 | 0.174937 | -0.71318 | CDK6 |
| 243059_at | 1.34557 | 0.428217 | 1.34557 | up | 1.349996 | 0.432955 | 1.349996 | up | 1.739072 | 0.798317 | 1.739072 | up | 1.41758 | 0.50343 | 1.41758 | up | 0 | 0.428217 | 0.432955 | 0.798317 | 0.50343 | FENDRR |
| 243110_x_at | 1.314496 | 0.394509 | 1.314496 | up | 1.413069 | 0.498832 | 1.413069 | up | 1.268838 | 0.343508 | 1.268838 | up | 1.77914 | 0.83118 | 1.77914 | up | 0 | 0.394509 | 0.498832 | 0.343508 | 0.83118 | NPW |
| 243155_at | 1.591895 | 0.670745 | 1.591895 | up | 1.059996 | 0.084059 | 1.059996 | up | -1.07254 | -0.10104 | 1.072545 | down | 1.059996 | 0.084059 | 1.059996 | up | 0 | 0.670745 | 0.084059 | -0.10104 | 0.084059 | LOC105378074 |
| 243166_at | -1.38182 | -0.46656 | 1.381815 | down | -1.11359 | -0.15522 | 1.113588 | down | -1.07454 | -0.10372 | 1.07454 | down | -1.52637 | -0.6101 | 1.526366 | down | 0 | -0.46656 | -0.15522 | -0.10372 | -0.6101 | SLC30A5 |
| 243198_at | -1.2093 | -0.27417 | 1.209297 | down | -1.05068 | -0.07133 | 1.050685 | down | -1.17259 | -0.2297 | 1.172594 | down | -1.53939 | -0.62235 | 1.539385 | down | 0 | -0.27417 | -0.07133 | -0.2297 | -0.62235 | TEX9 |
| 243267_x_at | 1.171443 | 0.228286 | 1.171443 | up | 1.042997 | 0.060735 | 1.042997 | up | 1.124858 | 0.169743 | 1.124858 | up | 1.505675 | 0.59041 | 1.505675 | up | 0 | 0.228286 | 0.060735 | 0.169743 | 0.59041 |  |
| 243303_at | 1.167296 | 0.223171 | 1.167296 | up | 1.167296 | 0.223171 | 1.167296 | up | 1.539972 | 0.622904 | 1.539972 | up | 1.439296 | 0.525364 | 1.439296 | up | 0 | 0.223171 | 0.223171 | 0.622904 | 0.525364 |  |
| 243367_at | -1.13505 | -0.18276 | 1.135051 | down | -1.21562 | -0.28169 | 1.215615 | down | -1.14432 | -0.19449 | 1.144317 | down | -1.5072 | -0.59187 | 1.507203 | down | 0 | -0.18276 | -0.28169 | -0.19449 | -0.59187 |  |
| 243371_at | -2.03464 | -1.02478 | 2.034644 | down | -1.35688 | -0.44029 | 1.356881 | down | -1.35688 | -0.44029 | 1.356881 | down | -1.35688 | -0.44029 | 1.356881 | down | 0 | -1.02478 | -0.44029 | -0.44029 | -0.44029 |  |
| 243481_at | -1.26521 | -0.33937 | 1.265207 | down | -1.26521 | -0.33937 | 1.265207 | down | -1.56353 | -0.6448 | 1.563526 | down | -1.28631 | -0.36324 | 1.286312 | down | 0 | -0.33937 | -0.33937 | -0.6448 | -0.36324 | RHOJ |
| 243614_s_at | -1 | 0 | 1 | down | -1.65999 | -0.73118 | 1.659994 | down | -1 | 0 | 1 | down | 1.32187 | 0.402581 | 1.32187 | up | 0 | 0 | -0.73118 | 0 | 0.402581 | PRODH2 |
| 244079_at | -1.29903 | -0.37743 | 1.299026 | down | -1.50848 | -0.5931 | 1.508484 | down | -1.13813 | -0.18667 | 1.138132 | down | -1.06427 | -0.08987 | 1.064272 | down | 0 | -0.37743 | -0.5931 | -0.18667 | -0.08987 | LRRC72 |
| 244184_at | -1.28443 | -0.36113 | 1.284429 | down | -1.05166 | -0.07267 | 1.051662 | down | -1.02989 | -0.04249 | 1.029893 | down | 1.635805 | 0.710001 | 1.635805 | up | 0 | -0.36113 | -0.07267 | -0.04249 | 0.710001 |  |
| 244349_at | -1.55514 | -0.63704 | 1.555136 | down | -1.42583 | -0.5118 | 1.425833 | down | -1.41721 | -0.50306 | 1.417212 | down | -1.33347 | -0.41519 | 1.333472 | down | 0 | -0.63704 | -0.5118 | -0.50306 | -0.41519 |  |
| 244506_at | 1.701553 | 0.766852 | 1.701553 | up | 1.469388 | 0.555215 | 1.469388 | up | 1.469388 | 0.555215 | 1.469388 | up | 1.469388 | 0.555215 | 1.469388 | up | 0 | 0.766852 | 0.555215 | 0.555215 | 0.555215 | TMTC1 |
| 244787_at | -1.28706 | -0.36408 | 1.287064 | down | -1.28706 | -0.36408 | 1.287064 | down | -1.17262 | -0.22973 | 1.172616 | down | -1.52412 | -0.60798 | 1.524124 | down | 0 | -0.36408 | -0.36408 | -0.22973 | -0.60798 |  |
| 1552630_a_at | 1.214917 | 0.280857 | 1.214917 | up | 1.214917 | 0.280857 | 1.214917 | up | 1.55841 | 0.640075 | 1.55841 | up | 1.563967 | 0.64521 | 1.563967 | up | 0 | 0.280857 | 0.280857 | 0.640075 | 0.64521 | SRCAP |
| 1552641_s_at | -1.02086 | -0.02978 | 1.020856 | down | 1.319858 | 0.400383 | 1.319858 | up | 1.377269 | 0.461811 | 1.377269 | up | 1.649978 | 0.722447 | 1.649978 | up | 0 | -0.02978 | 0.400383 | 0.461811 | 0.722447 | ATAD3A///ATAD3B |
| 1552656_s_at | 1.340925 | 0.423228 | 1.340925 | up | 1.571736 | 0.652359 | 1.571736 | up | 1.542402 | 0.625179 | 1.542402 | up | 1.193406 | 0.255085 | 1.193406 | up | 0 | 0.423228 | 0.652359 | 0.625179 | 0.255085 | UHMK1 |
| 1552667_a_at | -1.50214 | -0.58702 | 1.502136 | down | -1.0716 | -0.09976 | 1.071598 | down | -1.05366 | -0.0754 | 1.053656 | down | 1.231592 | 0.300524 | 1.231592 | up | 0 | -0.58702 | -0.09976 | -0.0754 | 0.300524 | SH2D3C |
| 1552687_a_at | -1.73705 | -0.79664 | 1.737049 | down | -1.31631 | -0.3965 | 1.316307 | down | -1.29296 | -0.37067 | 1.292957 | down | -1.28176 | -0.35813 | 1.281762 | down | 0 | -0.79664 | -0.3965 | -0.37067 | -0.35813 | CNBD2 |
| 1552737_s_at | 1.314761 | 0.3948 | 1.314761 | up | 2.120729 | 1.08456 | 2.120729 | up | 1.665743 | 0.736166 | 1.665743 | up | 1.565152 | 0.646302 | 1.565152 | up | 0 | 0.3948 | 1.08456 | 0.736166 | 0.646302 | WWP2 |
| 1553141_at | 1.482353 | 0.567889 | 1.482353 | up | 1.506318 | 0.591026 | 1.506318 | up | 1.856095 | 0.892271 | 1.856095 | up | 1.360869 | 0.444529 | 1.360869 | up | 0 | 0.567889 | 0.591026 | 0.892271 | 0.444529 | LACC1 |
| 1553142_at | 1.163878 | 0.21894 | 1.163878 | up | 1.10523 | 0.144346 | 1.10523 | up | 1.512665 | 0.597092 | 1.512665 | up | 1.019094 | 0.027287 | 1.019094 | up | 0 | 0.21894 | 0.144346 | 0.597092 | 0.027287 | LACC1 |
| 1553304_at | 1.417185 | 0.503028 | 1.417185 | up | 1.516817 | 0.601047 | 1.516817 | up | 1.507371 | 0.592034 | 1.507371 | up | 1.437666 | 0.523729 | 1.437666 | up | 0 | 0.503028 | 0.601047 | 0.592034 | 0.523729 | LSM14B |
| 1553535_a_at | 1.075708 | 0.105287 | 1.075708 | up | 1.73297 | 0.793246 | 1.73297 | up | 1.478 | 0.563646 | 1.478 | up | 1.991642 | 0.993959 | 1.991642 | up | 0 | 0.105287 | 0.793246 | 0.563646 | 0.993959 | RANGAP1 |
| 1553538_s_at | 1.509205 | 0.593789 | 1.509205 | up | 1.009085 | 0.013047 | 1.009085 | up | 1.429198 | 0.515205 | 1.429198 | up | -1.17676 | -0.23482 | 1.176762 | down | 0 | 0.593789 | 0.013047 | 0.515205 | -0.23482 | COX1 |
| 1553542_at | 1.366761 | 0.450761 | 1.366761 | up | 1.366761 | 0.450761 | 1.366761 | up | 1.307344 | 0.386639 | 1.307344 | up | 1.501115 | 0.586034 | 1.501115 | up | 0 | 0.450761 | 0.450761 | 0.386639 | 0.586034 | CCDC125 |
| 1553551_s_at | 1.821171 | 0.864866 | 1.821171 | up | 1.384746 | 0.469622 | 1.384746 | up | 1.574529 | 0.654921 | 1.574529 | up | -1.08609 | -0.11914 | 1.086088 | down | 0 | 0.864866 | 0.469622 | 0.654921 | -0.11914 | ND2 |
| 1553567_s_at | 1.625804 | 0.701154 | 1.625804 | up | 1.288492 | 0.365684 | 1.288492 | up | 1.621173 | 0.697038 | 1.621173 | up | -1.25871 | -0.33195 | 1.25871 | down | 0 | 0.701154 | 0.365684 | 0.697038 | -0.33195 | ATP6 |
| 1553569_at | 1.608304 | 0.68554 | 1.608304 | up | 1.391842 | 0.476995 | 1.391842 | up | 1.555922 | 0.63777 | 1.555922 | up | -1.12684 | -0.17229 | 1.126843 | down | 0 | 0.68554 | 0.476995 | 0.63777 | -0.17229 | COX2 |
| 1553570_x_at | 1.566125 | 0.6472 | 1.566125 | up | 1.247226 | 0.318723 | 1.247226 | up | 1.462472 | 0.548409 | 1.462472 | up | -1.18575 | -0.2458 | 1.185749 | down | 0 | 0.6472 | 0.318723 | 0.548409 | -0.2458 | COX2 |
| 1553575_at | 1.737417 | 0.796944 | 1.737417 | up | 1.319199 | 0.399663 | 1.319199 | up | 1.319199 | 0.399663 | 1.319199 | up | -1.18351 | -0.24308 | 1.183513 | down | 0 | 0.796944 | 0.399663 | 0.399663 | -0.24308 | ND6 |
| 1553584_at | -1.2245 | -0.29219 | 1.224498 | down | -1.13896 | -0.18771 | 1.138956 | down | -1.13896 | -0.18771 | 1.138956 | down | -1.50441 | -0.5892 | 1.504414 | down | 0 | -0.29219 | -0.18771 | -0.18771 | -0.5892 | CXorf36 |
| 1554027_a_at | -1.09352 | -0.12898 | 1.093518 | down | 1.024276 | 0.034605 | 1.024276 | up | -1.0286 | -0.04068 | 1.028596 | down | -1.62822 | -0.7033 | 1.62822 | down | 0 | -0.12898 | 0.034605 | -0.04068 | -0.7033 | SLC4A4 |
| 1554201_at | -1.09131 | -0.12606 | 1.091308 | down | 1.503502 | 0.588326 | 1.503502 | up | 1.024489 | 0.034905 | 1.024489 | up | -1.04963 | -0.06988 | 1.049633 | down | 0 | -0.12606 | 0.588326 | 0.034905 | -0.06988 | CABP4 |
| 1554314_at | -1.26344 | -0.33736 | 1.263445 | down | -1.29739 | -0.37561 | 1.297391 | down | -1.29739 | -0.37561 | 1.297391 | down | -1.81752 | -0.86197 | 1.817525 | down | 0 | -0.33736 | -0.37561 | -0.37561 | -0.86197 | C6orf141 |
| 1554410_a_at | 1.217227 | 0.283598 | 1.217227 | up | 1.400311 | 0.485747 | 1.400311 | up | 1.312313 | 0.392112 | 1.312313 | up | 1.661958 | 0.732883 | 1.661958 | up | 0 | 0.283598 | 0.485747 | 0.392112 | 0.732883 | WBSCR16 |
| 1554980_a_at | 1.167738 | 0.223717 | 1.167738 | up | 1.555925 | 0.637773 | 1.555925 | up | 1.647656 | 0.720415 | 1.647656 | up | 1.660721 | 0.73181 | 1.660721 | up | 0 | 0.223717 | 0.637773 | 0.720415 | 0.73181 | ATF3 |
| 1554491_a_at | -1.58087 | -0.66072 | 1.58087 | down | -1.36004 | -0.44365 | 1.360041 | down | -1.43273 | -0.51877 | 1.432733 | down | -1.36004 | -0.44365 | 1.360041 | down | 0 | -0.66072 | -0.44365 | -0.51877 | -0.44365 | SERPINC1 |
| 1554574_a_at | 1.095005 | 0.130937 | 1.095005 | up | 1.539511 | 0.622472 | 1.539511 | up | 1.396545 | 0.481862 | 1.396545 | up | 1.819462 | 0.863512 | 1.819462 | up | 0 | 0.130937 | 0.622472 | 0.481862 | 0.863512 | CYB5R3 |
| 1555294_a_at | 1.014195 | 0.020336 | 1.014195 | up | 1.306282 | 0.385466 | 1.306282 | up | 1.197503 | 0.260029 | 1.197503 | up | 1.563206 | 0.644508 | 1.563206 | up | 0 | 0.020336 | 0.385466 | 0.260029 | 0.644508 | ERC1 |
| 1554613_a_at | 1.526492 | 0.61022 | 1.526492 | up | 1.433417 | 0.519458 | 1.433417 | up | 1.461077 | 0.547032 | 1.461077 | up | 1.417551 | 0.503401 | 1.417551 | up | 0 | 0.61022 | 0.519458 | 0.547032 | 0.503401 | RUBCN |
| 1554672_at | -1.58727 | -0.66655 | 1.587269 | down | -1.10765 | -0.14751 | 1.107653 | down | -1.10765 | -0.14751 | 1.107653 | down | -1.28137 | -0.35769 | 1.281375 | down | 0 | -0.66655 | -0.14751 | -0.14751 | -0.35769 | TTC26 |
| 1554703_at | 1.536733 | 0.619867 | 1.536733 | up | -1 | 0 | 1 | down | -1.04252 | -0.06008 | 1.042524 | down | -1.11454 | -0.15645 | 1.114537 | down | 0 | 0.619867 | 0 | -0.06008 | -0.15645 | ARHGEF10 |
| 1554769_at | 1.44414 | 0.530211 | 1.44414 | up | 1.10191 | 0.140006 | 1.10191 | up | 1.510553 | 0.595077 | 1.510553 | up | 1.313553 | 0.393475 | 1.313553 | up | 0 | 0.530211 | 0.140006 | 0.595077 | 0.393475 | ZNF785 |
| 1554885_a_at | 1.058291 | 0.081736 | 1.058291 | up | 1.549063 | 0.631395 | 1.549063 | up | 1.371058 | 0.45529 | 1.371058 | up | 1.238785 | 0.308926 | 1.238785 | up | 0 | 0.081736 | 0.631395 | 0.45529 | 0.308926 | PRIM2 |
| 1554966_a_at | -1.05425 | -0.07622 | 1.054252 | down | -1.11518 | -0.15728 | 1.115179 | down | -1.09444 | -0.1302 | 1.094444 | down | -1.68054 | -0.74893 | 1.680541 | down | 0 | -0.07622 | -0.15728 | -0.1302 | -0.74893 | FILIP1L |
| 1555019_at | -1.59178 | -0.67065 | 1.591785 | down | -1.1866 | -0.24683 | 1.186598 | down | -1.29404 | -0.37188 | 1.294039 | down | -1.59707 | -0.67543 | 1.59707 | down | 0 | -0.67065 | -0.24683 | -0.37188 | -0.67543 | CDHR1 |
| 1555039_a_at | 1.413157 | 0.498922 | 1.413157 | up | 1.511976 | 0.596436 | 1.511976 | up | 1.401596 | 0.48707 | 1.401596 | up | 1.182691 | 0.242074 | 1.182691 | up | 0 | 0.498922 | 0.596436 | 0.48707 | 0.242074 | ABCC4 |
| 1555229_a_at | 1.128618 | 0.174558 | 1.128618 | up | 1.631897 | 0.70655 | 1.631897 | up | 1.26811 | 0.34268 | 1.26811 | up | 1.510317 | 0.594851 | 1.510317 | up | 0 | 0.174558 | 0.70655 | 0.34268 | 0.594851 | C1S |
| 1555266_a_at | -1.45597 | -0.54198 | 1.455967 | down | -1.30643 | -0.38563 | 1.306427 | down | -1.62329 | -0.69892 | 1.623287 | down | -1.14432 | -0.19449 | 1.144321 | down | 0 | -0.54198 | -0.38563 | -0.69892 | -0.19449 | ASXL2 |
| 1555349_a_at | -1.86886 | -0.90215 | 1.868855 | down | -1.27984 | -0.35597 | 1.279845 | down | -1.30372 | -0.38264 | 1.303724 | down | -1.42852 | -0.51453 | 1.428525 | down | 0 | -0.90215 | -0.35597 | -0.38264 | -0.51453 | ITGB2 |
| 1555467_a_at | 1.211425 | 0.276705 | 1.211425 | up | 1.644639 | 0.717771 | 1.644639 | up | 1.552436 | 0.634533 | 1.552436 | up | 1.458838 | 0.54482 | 1.458838 | up | 0 | 0.276705 | 0.717771 | 0.634533 | 0.54482 | CELF1 |
| 1555554_at | -1.11836 | -0.16139 | 1.118365 | down | 1.542431 | 0.625206 | 1.542431 | up | 1.193862 | 0.255636 | 1.193862 | up | 1.072765 | 0.101334 | 1.072765 | up | 0 | -0.16139 | 0.625206 | 0.255636 | 0.101334 | BPIFA4P |
| 1555561_a_at | -1 | 0 | 1 | down | 1.02635 | 0.037523 | 1.02635 | up | -1 | 0 | 1 | down | -1.59778 | -0.67607 | 1.59778 | down | 0 | 0 | 0.037523 | 0 | -0.67607 | UGGT2 |
| 1555609_a_at | 1.47025 | 0.556062 | 1.47025 | up | 1.324481 | 0.405427 | 1.324481 | up | 1.628304 | 0.70337 | 1.628304 | up | 1.498026 | 0.583062 | 1.498026 | up | 0 | 0.556062 | 0.405427 | 0.70337 | 0.583062 | ZMAT3 |
| 1555611_s_at | 1.262944 | 0.336791 | 1.262944 | up | 1.601247 | 0.679195 | 1.601247 | up | 1.296945 | 0.375117 | 1.296945 | up | 1.770952 | 0.824525 | 1.770952 | up | 0 | 0.336791 | 0.679195 | 0.375117 | 0.824525 | MBD1 |
| 1555653_at | 1.676618 | 0.745554 | 1.676618 | up | 1.173651 | 0.231003 | 1.173651 | up | 1.398346 | 0.483722 | 1.398346 | up | -1.13208 | -0.17898 | 1.13208 | down | 0 | 0.745554 | 0.231003 | 0.483722 | -0.17898 |  |
| 1555704_at | -1.19244 | -0.25391 | 1.192437 | down | -1.58411 | -0.66367 | 1.584108 | down | -1.21973 | -0.28656 | 1.219727 | down | -1.11025 | -0.15088 | 1.110246 | down | 0 | -0.25391 | -0.66367 | -0.28656 | -0.15088 | CMTM3 |
| 1555730_a_at | 1.045942 | 0.064802 | 1.045942 | up | 1.445824 | 0.531892 | 1.445824 | up | 1.296829 | 0.374989 | 1.296829 | up | 1.612852 | 0.689614 | 1.612852 | up | 0 | 0.064802 | 0.531892 | 0.374989 | 0.689614 | CFL1 |
| 1555736_a_at | 1.023966 | 0.034167 | 1.023966 | up | 1.422602 | 0.508532 | 1.422602 | up | 1.211887 | 0.277255 | 1.211887 | up | 1.550868 | 0.633076 | 1.550868 | up | 0 | 0.034167 | 0.508532 | 0.277255 | 0.633076 | AGTRAP |
| 1555784_s_at | -1.10713 | -0.14682 | 1.107128 | down | 1.34812 | 0.430949 | 1.34812 | up | 1.216608 | 0.282864 | 1.216608 | up | 1.587451 | 0.666712 | 1.587451 | up | 0 | -0.14682 | 0.430949 | 0.282864 | 0.666712 | IRAK1 |
| 1555788_a_at | 1.118461 | 0.161514 | 1.118461 | up | 1.608439 | 0.685661 | 1.608439 | up | 1.373417 | 0.457769 | 1.373417 | up | 1.445766 | 0.531834 | 1.445766 | up | 0 | 0.161514 | 0.685661 | 0.457769 | 0.531834 | TRIB3 |
| 1555789_s_at | 1.200182 | 0.263254 | 1.200182 | up | 1.481997 | 0.567542 | 1.481997 | up | 1.292516 | 0.370182 | 1.292516 | up | 1.54414 | 0.626804 | 1.54414 | up | 0 | 0.263254 | 0.567542 | 0.370182 | 0.626804 | PHF23 |
| 1555878_at | 1.174316 | 0.231821 | 1.174316 | up | -1.00346 | -0.00498 | 1.003459 | down | 1.230543 | 0.299295 | 1.230543 | up | -1.67379 | -0.74312 | 1.673793 | down | 0 | 0.231821 | -0.00498 | 0.299295 | -0.74312 | RPS24 |
| 1555978_s_at | -1.0177 | -0.02531 | 1.017701 | down | 1.161255 | 0.215684 | 1.161255 | up | 1.155789 | 0.208878 | 1.155789 | up | -1.71286 | -0.77641 | 1.712865 | down | 0 | -0.02531 | 0.215684 | 0.208878 | -0.77641 | MYL12A |
| 1556187_at | -1.35772 | -0.44119 | 1.357719 | down | -1.35772 | -0.44119 | 1.357719 | down | -1.59729 | -0.67562 | 1.597288 | down | -1.35772 | -0.44119 | 1.357719 | down | 0 | -0.44119 | -0.44119 | -0.67562 | -0.44119 | ZNF555 |
| 1556249_a_at | -1.33629 | -0.41823 | 1.33629 | down | -1.13281 | -0.17991 | 1.132811 | down | -1.59562 | -0.67411 | 1.595617 | down | -1.11618 | -0.15857 | 1.116183 | down | 0 | -0.41823 | -0.17991 | -0.67411 | -0.15857 | EIF1B-AS1 |
| 1556266_a_at | 1.201438 | 0.264762 | 1.201438 | up | -1.18814 | -0.2487 | 1.188137 | down | 1.291604 | 0.369164 | 1.291604 | up | 1.52662 | 0.610341 | 1.52662 | up | 0 | 0.264762 | -0.2487 | 0.369164 | 0.610341 | C20orf202 |
| 1556332_at | 1.029685 | 0.042203 | 1.029685 | up | -1.16794 | -0.22397 | 1.16794 | down | -1.08839 | -0.1222 | 1.088393 | down | -1.53433 | -0.61761 | 1.53433 | down | 0 | 0.042203 | -0.22397 | -0.1222 | -0.61761 |  |
| 1556368_at | 1.535816 | 0.619006 | 1.535816 | up | 1.409628 | 0.495315 | 1.409628 | up | 1.121182 | 0.165021 | 1.121182 | up | 1.19706 | 0.259495 | 1.19706 | up | 0 | 0.619006 | 0.495315 | 0.165021 | 0.259495 | PHKG2 |
| 1556413_a_at | 1.167586 | 0.223529 | 1.167586 | up | 1.167586 | 0.223529 | 1.167586 | up | 1.241602 | 0.312203 | 1.241602 | up | 1.578491 | 0.658546 | 1.578491 | up | 0 | 0.223529 | 0.223529 | 0.312203 | 0.658546 |  |
| 1556453_at | 1.643451 | 0.716728 | 1.643451 | up | 1.2692 | 0.343919 | 1.2692 | up | -1.09025 | -0.12466 | 1.090252 | down | 1.486276 | 0.571703 | 1.486276 | up | 0 | 0.716728 | 0.343919 | -0.12466 | 0.571703 | LOC100506274 |
| 1556608_a_at | 1.522107 | 0.606069 | 1.522107 | up | 1.155329 | 0.208304 | 1.155329 | up | 1.105562 | 0.144779 | 1.105562 | up | 1.105562 | 0.144779 | 1.105562 | up | 0 | 0.606069 | 0.208304 | 0.144779 | 0.144779 | EHD4 |
| 1556698_a_at | -1.57547 | -0.65578 | 1.57547 | down | -1.28417 | -0.36083 | 1.284167 | down | -1.53787 | -0.62093 | 1.537871 | down | -1.19532 | -0.2574 | 1.195319 | down | 0 | -0.65578 | -0.36083 | -0.62093 | -0.2574 | GPRIN3 |
| 1556744_a_at | 1.871364 | 0.90409 | 1.871364 | up | 1.380462 | 0.465152 | 1.380462 | up | 1.380462 | 0.465152 | 1.380462 | up | 1.380462 | 0.465152 | 1.380462 | up | 0 | 0.90409 | 0.465152 | 0.465152 | 0.465152 | LOC105377200 |
| 1556823_s_at | -1.72354 | -0.78538 | 1.723543 | down | -1.04528 | -0.06388 | 1.045275 | down | -1.32851 | -0.40981 | 1.328514 | down | -1.24172 | -0.31234 | 1.241722 | down | 0 | -0.78538 | -0.06388 | -0.40981 | -0.31234 |  |
| 1556984_at | -1.24528 | -0.31647 | 1.24528 | down | -1.50814 | -0.59277 | 1.508145 | down | -1.24528 | -0.31647 | 1.24528 | down | -1.40347 | -0.489 | 1.403474 | down | 0 | -0.31647 | -0.59277 | -0.31647 | -0.489 |  |
| 1557179_s_at | 1.647965 | 0.720686 | 1.647965 | up | -1 | 0 | 1 | down | -1 | 0 | 1 | down | -1.13703 | -0.18527 | 1.137032 | down | 0 | 0.720686 | 0 | 0 | -0.18527 | LOC105376684 |
| 1557312_at | -1.7185 | -0.78115 | 1.718498 | down | -1.05578 | -0.07831 | 1.05578 | down | -1.1337 | -0.18103 | 1.133696 | down | -1.34612 | -0.42881 | 1.346122 | down | 0 | -0.78115 | -0.07831 | -0.18103 | -0.42881 | LINC01465 |
| 1557452_at | -1.73961 | -0.79877 | 1.739613 | down | -1.26754 | -0.34204 | 1.267545 | down | -1.42382 | -0.50977 | 1.423822 | down | -1.57211 | -0.6527 | 1.572111 | down | 0 | -0.79877 | -0.34204 | -0.50977 | -0.6527 |  |
| 1557661_at | -1.64608 | -0.71903 | 1.646078 | down | -1.59428 | -0.67291 | 1.594285 | down | -1.59428 | -0.67291 | 1.594285 | down | -1.59428 | -0.67291 | 1.594285 | down | 0 | -0.71903 | -0.67291 | -0.67291 | -0.67291 |  |
| 1557754_at | -1.06933 | -0.0967 | 1.069326 | down | -1.41578 | -0.5016 | 1.415782 | down | -1.45873 | -0.54472 | 1.458734 | down | -1.55557 | -0.63744 | 1.555571 | down | 0 | -0.0967 | -0.5016 | -0.54472 | -0.63744 | LOC105377135///LOC401068 |
| 1557789_at | -1.68623 | -0.7538 | 1.686229 | down | -1 | 0 | 1 | down | -1 | 0 | 1 | down | 1.091076 | 0.125752 | 1.091076 | up | 0 | -0.7538 | 0 | 0 | 0.125752 |  |
| 1558028_x_at | 1.142903 | 0.192702 | 1.142903 | up | 1.309422 | 0.38893 | 1.309422 | up | 1.790336 | 0.84023 | 1.790336 | up | 1.208621 | 0.273362 | 1.208621 | up | 0 | 0.192702 | 0.38893 | 0.84023 | 0.273362 | LINC00657 |
| 1558214_s_at | 1.168949 | 0.225212 | 1.168949 | up | 1.458339 | 0.544326 | 1.458339 | up | 1.392748 | 0.477934 | 1.392748 | up | 1.749832 | 0.807217 | 1.749832 | up | 0 | 0.225212 | 0.544326 | 0.477934 | 0.807217 | CTNNA1 |
| 1558308_at | 1.201296 | 0.264591 | 1.201296 | up | 1.201296 | 0.264591 | 1.201296 | up | 1.215396 | 0.281426 | 1.215396 | up | 1.542221 | 0.625009 | 1.542221 | up | 0 | 0.264591 | 0.264591 | 0.281426 | 0.625009 | LINC00942 |
| 1558378_a_at | 1.331115 | 0.412635 | 1.331115 | up | 1.331115 | 0.412635 | 1.331115 | up | 1.331115 | 0.412635 | 1.331115 | up | 1.634486 | 0.708837 | 1.634486 | up | 0 | 0.412635 | 0.412635 | 0.412635 | 0.708837 | AHNAK2 |
| 1558678_s_at | 1.733029 | 0.793295 | 1.733029 | up | 1.18022 | 0.239056 | 1.18022 | up | 1.18022 | 0.239056 | 1.18022 | up | 1.141894 | 0.191429 | 1.141894 | up | 0 | 0.793295 | 0.239056 | 0.239056 | 0.191429 | MALAT1 |
| 1559025_at | -1.42136 | -0.50727 | 1.421361 | down | -1.62282 | -0.6985 | 1.622816 | down | -1.78796 | -0.83831 | 1.78796 | down | -1.6964 | -0.76248 | 1.696403 | down | 0 | -0.50727 | -0.6985 | -0.83831 | -0.76248 | 9-Sep |
| 1559714_at | -1.63276 | -0.70731 | 1.632762 | down | -1.09798 | -0.13485 | 1.097981 | down | -1.09798 | -0.13485 | 1.097981 | down | -1.47536 | -0.56107 | 1.475359 | down | 0 | -0.70731 | -0.13485 | -0.13485 | -0.56107 | RGR |
| 1559965_at | -1.25331 | -0.32575 | 1.253312 | down | -1.57396 | -0.6544 | 1.573961 | down | -1.07801 | -0.10837 | 1.078008 | down | -1.24256 | -0.31332 | 1.242563 | down | 0 | -0.32575 | -0.6544 | -0.10837 | -0.31332 | ZFHX4-AS1 |
| 1560418_at | -1.35696 | -0.44038 | 1.356959 | down | -1.37831 | -0.4629 | 1.378308 | down | -1.51088 | -0.59539 | 1.51088 | down | -1.37831 | -0.4629 | 1.378308 | down | 0 | -0.44038 | -0.4629 | -0.59539 | -0.4629 | CEP57L1 |
| 1560550_at | 1.5051 | 0.58986 | 1.5051 | up | 1.291257 | 0.368776 | 1.291257 | up | 1.42132 | 0.507231 | 1.42132 | up | 1.42132 | 0.507231 | 1.42132 | up | 0 | 0.58986 | 0.368776 | 0.507231 | 0.507231 | LL22NC03-75H12.2 |
| 1560727_at | -1.52833 | -0.61196 | 1.52833 | down | -1 | 0 | 1 | down | -1 | 0 | 1 | down | -1 | 0 | 1 | down | 0 | -0.61196 | 0 | 0 | 0 | HEATR4 |
| 1560851_at | -1.10306 | -0.14152 | 1.103065 | down | -1.07088 | -0.0988 | 1.070882 | down | -1.144 | -0.19408 | 1.143996 | down | -1.50195 | -0.58683 | 1.501947 | down | 0 | -0.14152 | -0.0988 | -0.19408 | -0.58683 | LINC00619 |
| 1560973_a_at | 1.528647 | 0.612255 | 1.528647 | up | -1 | 0 | 1 | down | -1.0694 | -0.0968 | 1.069399 | down | -1 | 0 | 1 | down | 0 | 0.612255 | 0 | -0.0968 | 0 |  |
| 1561004_at | -1.04304 | -0.06079 | 1.04304 | down | -1.50568 | -0.59042 | 1.505684 | down | -1.2465 | -0.31788 | 1.246497 | down | -1.15637 | -0.20961 | 1.156375 | down | 0 | -0.06079 | -0.59042 | -0.31788 | -0.20961 | PRKCQ-AS1 |
| 1561088_at | 1.212787 | 0.278326 | 1.212787 | up | 1.065955 | 0.092146 | 1.065955 | up | 1.458334 | 0.544321 | 1.458334 | up | 1.526787 | 0.610498 | 1.526787 | up | 0 | 0.278326 | 0.092146 | 0.544321 | 0.610498 | EYA4 |
| 1569452_at | 1.398135 | 0.483504 | 1.398135 | up | 1.398135 | 0.483504 | 1.398135 | up | 1.590156 | 0.669169 | 1.590156 | up | 1.510643 | 0.595163 | 1.510643 | up | 0 | 0.483504 | 0.483504 | 0.669169 | 0.595163 | LOC692247 |
| 1561642_at | 1.236522 | 0.306288 | 1.236522 | up | 1.236522 | 0.306288 | 1.236522 | up | 1.642907 | 0.716251 | 1.642907 | up | 1.236522 | 0.306288 | 1.236522 | up | 0 | 0.306288 | 0.306288 | 0.716251 | 0.306288 |  |
| 1561928_s_at | -1.00689 | -0.00991 | 1.00689 | down | -1.07034 | -0.09807 | 1.070338 | down | -1.00689 | -0.00991 | 1.00689 | down | 1.539448 | 0.622413 | 1.539448 | up | 0 | -0.00991 | -0.09807 | -0.00991 | 0.622413 | ANKUB1 |
| 1561995_at | -1.34582 | -0.42848 | 1.345815 | down | -1.55395 | -0.63594 | 1.553953 | down | -1.37152 | -0.45578 | 1.37152 | down | -1.37152 | -0.45578 | 1.37152 | down | 0 | -0.42848 | -0.63594 | -0.45578 | -0.45578 |  |
| 1562573_at | 1.506898 | 0.591581 | 1.506898 | up | -1.14202 | -0.19159 | 1.142025 | down | 1.019022 | 0.027185 | 1.019022 | up | 1.166538 | 0.222233 | 1.166538 | up | 0 | 0.591581 | -0.19159 | 0.027185 | 0.222233 | CYP17A1 |
| 1562722_at | -1.64313 | -0.71645 | 1.643132 | down | -1.40204 | -0.48753 | 1.402038 | down | -1.35275 | -0.43589 | 1.352748 | down | -1.46525 | -0.55115 | 1.465251 | down | 0 | -0.71645 | -0.48753 | -0.43589 | -0.55115 | PRR20A///PRR20B///PRR20C///PRR20D///PRR20E |
| 1562836_at | 1.689816 | 0.756866 | 1.689816 | up | 1.329435 | 0.410813 | 1.329435 | up | 1.454887 | 0.540907 | 1.454887 | up | 1.399343 | 0.48475 | 1.399343 | up | 0 | 0.756866 | 0.410813 | 0.540907 | 0.48475 | DDX6 |
| 1562899_at | -1.31942 | -0.3999 | 1.31942 | down | -1.35 | -0.43296 | 1.349998 | down | -1.6377 | -0.71167 | 1.637703 | down | -1.47698 | -0.56265 | 1.476981 | down | 0 | -0.3999 | -0.43296 | -0.71167 | -0.56265 |  |
| 1563032_at | 1.662669 | 0.733501 | 1.662669 | up | 1.153525 | 0.206049 | 1.153525 | up | 1.153525 | 0.206049 | 1.153525 | up | 1.18544 | 0.245423 | 1.18544 | up | 0 | 0.733501 | 0.206049 | 0.206049 | 0.245423 |  |
| 1569106_s_at | 1.624803 | 0.700265 | 1.624803 | up | 1.827437 | 0.869822 | 1.827437 | up | 1.624803 | 0.700265 | 1.624803 | up | 2.129777 | 1.090703 | 2.129777 | up | 0 | 0.700265 | 0.869822 | 0.700265 | 1.090703 | SETD5 |
| 1564131_a_at | -1.00931 | -0.01337 | 1.00931 | down | -1.11593 | -0.15825 | 1.115933 | down | -1 | 0 | 1 | down | 1.508688 | 0.593295 | 1.508688 | up | 0 | -0.01337 | -0.15825 | 0 | 0.593295 | BSN-AS2 |
| 1563874_at | -1.53122 | -0.61468 | 1.53122 | down | -1.50986 | -0.59442 | 1.509862 | down | -1.50423 | -0.58903 | 1.504233 | down | -1.50423 | -0.58903 | 1.504233 | down | 0 | -0.61468 | -0.59442 | -0.58903 | -0.58903 | WDR72 |
| 1564004_at | 1.675268 | 0.744392 | 1.675268 | up | 1.15027 | 0.201972 | 1.15027 | up | 1.15027 | 0.201972 | 1.15027 | up | 1.15027 | 0.201972 | 1.15027 | up | 0 | 0.744392 | 0.201972 | 0.201972 | 0.201972 |  |
| 1564251_at | -1.51227 | -0.59671 | 1.512265 | down | -1.1775 | -0.23573 | 1.177499 | down | -1.11214 | -0.15334 | 1.112145 | down | -1.24699 | -0.31845 | 1.246987 | down | 0 | -0.59671 | -0.23573 | -0.15334 | -0.31845 | EMID1 |
| 1564494_s_at | 1.03313 | 0.047022 | 1.03313 | up | 1.571119 | 0.651793 | 1.571119 | up | 1.367629 | 0.451677 | 1.367629 | up | 1.860485 | 0.895679 | 1.860485 | up | 0 | 0.047022 | 0.651793 | 0.451677 | 0.895679 | P4HB |
| 1564601_at | 1.633366 | 0.707848 | 1.633366 | up | 1.353528 | 0.436725 | 1.353528 | up | 1.353528 | 0.436725 | 1.353528 | up | 1.353528 | 0.436725 | 1.353528 | up | 0 | 0.707848 | 0.436725 | 0.436725 | 0.436725 | LINC00221 |
| 1564733_at | -1.16324 | -0.21814 | 1.163235 | down | -1.1348 | -0.18244 | 1.1348 | down | -1.1348 | -0.18244 | 1.1348 | down | -1.6203 | -0.69626 | 1.620295 | down | 0 | -0.21814 | -0.18244 | -0.18244 | -0.69626 |  |
| 1564841_at | -1.16784 | -0.22384 | 1.167839 | down | 1.13456 | 0.182133 | 1.13456 | up | 1.333737 | 0.415474 | 1.333737 | up | 1.574134 | 0.654558 | 1.574134 | up | 0 | -0.22384 | 0.182133 | 0.415474 | 0.654558 | GABRA2 |
| 1565717_s_at | 1.211199 | 0.276436 | 1.211199 | up | 1.445376 | 0.531445 | 1.445376 | up | 1.347932 | 0.430748 | 1.347932 | up | 1.721107 | 0.783337 | 1.721107 | up | 0 | 0.276436 | 0.531445 | 0.430748 | 0.783337 | FUS |
| 1565887_at | 1.292444 | 0.370102 | 1.292444 | up | 1.223405 | 0.290902 | 1.223405 | up | 1.402804 | 0.488313 | 1.402804 | up | 1.669757 | 0.739638 | 1.669757 | up | 0 | 0.370102 | 0.290902 | 0.488313 | 0.739638 |  |
| 1565906_at | 1.337737 | 0.419794 | 1.337737 | up | 1.337737 | 0.419794 | 1.337737 | up | 1.337737 | 0.419794 | 1.337737 | up | 1.596804 | 0.675187 | 1.596804 | up | 0 | 0.419794 | 0.419794 | 0.419794 | 0.675187 | NADSYN1 |
| 1566471_at | -1.65876 | -0.73011 | 1.658763 | down | -1 | 0 | 1 | down | -1 | 0 | 1 | down | 1.053479 | 0.075162 | 1.053479 | up | 0 | -0.73011 | 0 | 0 | 0.075162 | LOC100996506 |
| 1567080_s_at | 1.082849 | 0.114831 | 1.082849 | up | 1.221903 | 0.289129 | 1.221903 | up | 1.221903 | 0.289129 | 1.221903 | up | 1.51623 | 0.600488 | 1.51623 | up | 0 | 0.114831 | 0.289129 | 0.289129 | 0.600488 | CLN6 |
| 1567334_at | -1.53716 | -0.62027 | 1.537165 | down | -1.23988 | -0.31021 | 1.239884 | down | -1.19223 | -0.25366 | 1.192229 | down | -1.19223 | -0.25366 | 1.192229 | down | 0 | -0.62027 | -0.31021 | -0.25366 | -0.25366 |  |
| 1567527_at | -1.15204 | -0.20419 | 1.152036 | down | -1.10338 | -0.14193 | 1.10338 | down | -1.11647 | -0.15894 | 1.116466 | down | -1.52564 | -0.60941 | 1.525636 | down | 0 | -0.20419 | -0.14193 | -0.15894 | -0.60941 |  |
| 1568889_at | -1.07392 | -0.10289 | 1.073923 | down | -1.15027 | -0.20197 | 1.15027 | down | -1.51759 | -0.60178 | 1.517588 | down | -1.05869 | -0.08229 | 1.058695 | down | 0 | -0.10289 | -0.20197 | -0.60178 | -0.08229 | FANCD2 |
| 1569061_at | -1.07679 | -0.10674 | 1.07679 | down | 1.559461 | 0.641048 | 1.559461 | up | 1.309635 | 0.389164 | 1.309635 | up | 1.241241 | 0.311783 | 1.241241 | up | 0 | -0.10674 | 0.641048 | 0.389164 | 0.311783 | IQGAP3 |
| 1569107_s_at | -1.13676 | -0.18493 | 1.136761 | down | -1.15898 | -0.21285 | 1.158978 | down | -1.13676 | -0.18493 | 1.136761 | down | -1.52324 | -0.60714 | 1.523242 | down | 0 | -0.18493 | -0.21285 | -0.18493 | -0.60714 | ZFP69 |
| 1569142_at | 1.516231 | 0.60049 | 1.516231 | up | 1.167296 | 0.223171 | 1.167296 | up | 1.42132 | 0.507231 | 1.42132 | up | 1.167296 | 0.223171 | 1.167296 | up | 0 | 0.60049 | 0.223171 | 0.507231 | 0.223171 | TRIM13 |
| 1569238_a_at | 1.504327 | 0.589118 | 1.504327 | up | 1.061717 | 0.086399 | 1.061717 | up | 1.113096 | 0.154578 | 1.113096 | up | 1.21768 | 0.284135 | 1.21768 | up | 0 | 0.589118 | 0.086399 | 0.154578 | 0.284135 |  |
| 1569253_at | -1.48104 | -0.56661 | 1.481039 | down | -1.5016 | -0.5865 | 1.501604 | down | -1.63862 | -0.71249 | 1.638625 | down | -2.03945 | -1.02818 | 2.039446 | down | 0 | -0.56661 | -0.5865 | -0.71249 | -1.02818 | INTS4 |
| 1569516_at | 1.545802 | 0.628356 | 1.545802 | up | 1.157885 | 0.211492 | 1.157885 | up | 1.277785 | 0.353645 | 1.277785 | up | 1.17338 | 0.23067 | 1.17338 | up | 0 | 0.628356 | 0.211492 | 0.353645 | 0.23067 |  |
| 1569569_x_at | -1.44679 | -0.53285 | 1.446789 | down | -1.26837 | -0.34298 | 1.268374 | down | -1.87876 | -0.90978 | 1.87876 | down | -1.44679 | -0.53285 | 1.446789 | down | 0 | -0.53285 | -0.34298 | -0.90978 | -0.53285 |  |
| 1570044_at | -1.21362 | -0.27932 | 1.21362 | down | -1.56435 | -0.64556 | 1.564346 | down | -1.21362 | -0.27932 | 1.21362 | down | -1.23051 | -0.29926 | 1.230513 | down | 0 | -0.27932 | -0.64556 | -0.27932 | -0.29926 | LOC101927126 |
| 1570131_at | -1.61152 | -0.68842 | 1.611518 | down | -1.13997 | -0.189 | 1.139973 | down | -1.13997 | -0.189 | 1.139973 | down | -1.094 | -0.12961 | 1.094 | down | 0 | -0.68842 | -0.189 | -0.189 | -0.12961 | LOC285847 |
